# TM2D3, a mammalian homologue of *Drosophila* neurogenic gene product Almondex, regulates surface presentation of Notch receptors

**DOI:** 10.1038/s41598-023-46866-7

**Published:** 2023-11-27

**Authors:** Wataru Masuda, Tomoko Yamakawa, Rieko Ajima, Katsuya Miyake, Toshifumi Umemiya, Kazuhiko Azuma, Jun-ichi Tamaru, Makoto Kiso, Puspa Das, Yumiko Saga, Kenji Matsuno, Motoo Kitagawa

**Affiliations:** 1 Department of Molecular and Tumor Pathology, Chiba University Graduate School of Medicine, 1-8-1 Inohana, Chuo-Ku, Chiba, 260-8670 Japan; 2 Department of Pathology, Saitama Medical Center, Saitama Medical University, 1981 Kamoda, Kawagoe, Saitama 350-8550 Japan; 3 Present Address: Department of Pathology, The Fraternity Memorial Hospital, Tokyo, Japan; 4 Department of Biological Sciences, Osaka University, 1-1 Machikaneyama, Toyonaka, Osaka 560-0043 Japan; 5 Present Address: Department of Chemistry, Bioengineering and Environmental Science, National Institute of Technology, Ibaraki College, Ibaraki, Japan; 6 Mammalian Development Laboratory, Department of Gene Function and Phenomics, National Institute of Genetics, 1111 Yata, Mishima, 411-8540 Japan; 7 Present Address: Division of Embryology, National Institute for Basic Biology, Okazaki, Japan; 8 Center for Basic Medical Research, International University of Health and Welfare, 4-3 Kozunomori, Narita, Chiba 286-8686 Japan; 9 International University of Health and Welfare Graduate School of Health and Welfare Sciences, 4-3 Kozunomori, Narita, Chiba 286-8686 Japan; 10 Department of Biochemistry, International University of Health and Welfare School of Medicine, 4-3 Kozunomori, Narita, Chiba 286-8686 Japan; 11 Department of Basic Medical Sciences, International University of Health and Welfare Graduate School of Medicine, 4-3 Kozunomori, Narita, Chiba 286-8686 Japan

**Keywords:** Cell signalling, Development

## Abstract

Notch signaling is an evolutionarily conserved mechanism required for numerous types of cell fate decisions in metazoans. It mediates short-range communication between cells with receptors and ligands, both of which are expressed on the cell surfaces. In response to the ligand-receptor interaction, the ligand and the extracellular domain of the Notch receptor (NECD) in the complex are internalized into ligand-expressing cells by endocytosis, a prerequisite process for the conformational change of the membrane proximal region of Notch to induce critical proteolytic cleavages for its activation. Here we report that overexpression of transmembrane 2 (TM2) domain containing 3 (TM2D3), a mammalian homologue of *Drosophila melanogaster* Almondex (Amx), activates Notch1. This activation requires the ligand-binding domain in Notch1 and the C-terminal region containing TM2 domain in TM2D3. TM2D3 physically associates with Notch1 at the region distinct from the ligand-binding domain and enhances expression of Notch1 on the cell surface. Furthermore, cell surface expression of Notch1 and Notch2 is reduced in *Tm2d3*-deficient cells. Finally, *amx*-deficient *Drosophila* early embryos exhibit impaired endocytosis of NECD and Delta ligand, for which surface presentation of Notch is required. These results indicate that TM2D3 is an element involved in Notch signaling through the surface presentation.

## Introduction

The Notch genes encode single-pass transmembrane receptor molecules. During maturation, Notch proteins are cleaved once at the extracellular region (S1) by furin-like protease in the trans-Golgi network (TGN)^1–3^. This modification has been shown to cause little change in the higher-order structure, yet it is required for efficient transport to the plasma membrane for certain types of Notch including Notch1^4^. Through a defined portion of its epidermal growth factor (EGF) repeat domain, Notch binds to its ligands (Delta or Serrate/Jagged)^5,6^ that are also expressed on the surfaces of neighboring cells^1–3,7^. In response to this engagement with Notch, ligands are endocytosed in complex with Notch extracellular domain (ECD) by the signal-sending cells, generating force on the receptor that is co-endocytosed^3,7–10^. This force leads to conformational change on the negative regulatory region (NRR) of Notch^3,7–10^, which consists of a heterodimerization domain (HD) and three Lin12/Notch repeats (LNR) that wrap around the HD at the luminal juxtamembrane portion^11,12^, rendering it sensitive to cleavage by A disintegrin and metalloproteinase (ADAM) 10 protease at a site (S2) in the HD^1,13–15^. This cleavage is followed by another, elicited by a tetrameric γ-secretase complex in the transmembrane domain (S3 site). This final processing releases the intracellular domain (NICD), the activated Notch, from the membrane; the NICD is then transported to the nucleus where it forms a ternary transcriptional activation complex with CSL (CBF1, Su(H), Lag-1) DNA-binding proteins and Mastermind co-activators^1–3,7,16^. This process is known as the canonical Notch signaling pathway^1^.

A unique characteristic of Notch signaling is that it lacks enzymatic amplification of the number for signal molecules and instead depends on stoichiometric interactions among elements of the pathway^2,7^. One molecule of Notch presented at the plasma membrane can generate at most only one NICD and one transcriptional activation complex. Perhaps for this reason, a modest alteration of gene dosage or expression of the components can substantially affect the phenotypes. In humans, various genetic and sporadic diseases including many types of cancers are linked to such changes^3^.

*almondex* (*amx*) is a member of the original group of neurogenic loci of *Drosophila*^17^. Its loss-of-function neurogenic phenotype (hyperplasia of the developing embryonic nervous system at the expense of developing ventral epidermis, an indication of loss of Notch signaling in flies) and its rescue by ectopic expression of NICD implicated *amx* as a positive regulatory element of the Notch signaling pathway in *Drosophila*^18,19^. The protein encoded by *amx* possesses an N-terminal signal peptide and two potential transmembrane domains at its C-terminal region (Supplementary Fig. S1 online)^19^. Recently, it was shown that *amx* is required for Notch signaling during mesectodermal specification that occurs prior to the neurogenesis and for proper subcellular distribution of Notch in the mesectodermal cells of the *Drosophila* embryo^20^.

A mammalian homologue of Amx, termed transmembrane 2 domain containing 3 (TM2D3) (also known as β-amyloid peptide binding protein-like protein 2 [BLP2]) has been identified based on the similarity in its primary structure (Supplementary Fig. S1 online)^19,21^. It has recently been shown that the human *TM2D3* gene can rescue the phenotype of the loss-of-function *amx* mutant of *Drosophila*, indicating that TM2D3 and Amx are orthologs and have conserved functions *in vivo*^22^. Interestingly, an exome-wide association analysis of a large cohort identified a variant of TM2D3 (P155L) as a strong risk factor for late-onset Alzheimer’s disease^22^. In contrast to the wild type TM2D3, the P155L variant could not rescue the *amx* mutant fly, indicating that the variant causes loss of function^22^. The mRNA of *TM2D3* has been shown to be expressed ubiquitously in all the human organs examined^21,22^. More recently, TM2D3 has been shown to be involved in the phagocytosis of a selected set of substances including amyloid-β aggregates in macrophages^23^.

In this work, we first sought to identify the functions of TM2D3 in Notch signaling using gain-of-function analyses in mammalian cultured cells to clarify its unique molecular mechanism of action. We next inactivated the *Tm2d3* gene in a mouse germline, prepared primary cultured cells from the mice, and performed in vitro loss-of-function analyses to verify the credibility of the results obtained by the gain-of-function analyses. Furthermore, we took advantage of an *amx*-deficient mutant of *Drosophila* for an in vivo loss-of-function analysis of the gene to obtain evidence consistent with the in vitro analyses. Together, these results show that TM2D3 is involved in Notch signaling, and we were able to clarify its mode of involvement.

## Results

### Characterization of TM2D3

**Figure 1:**
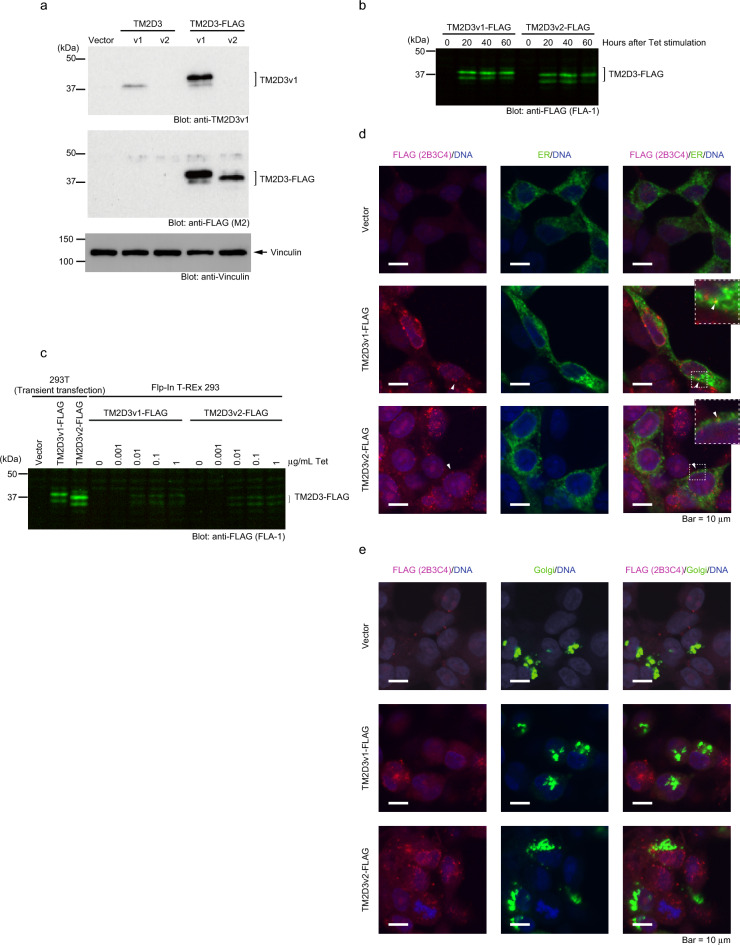
Characterization of TM2D3. (**a**) Expression of TM2D3 in transfected 293 T cells. 293 T cells were transiently transfected with expression vectors for indicated proteins or empty vector (Vector) as a control. Immunoblotting was performed with indicated antibodies. TM2D3v1 migrates slightly more slowly compared to TM2D3v2 probably due to its 26 additional amino acids. An anti-Vinculin blot serves as a loading control. (**b**) Induction of TM2D3 in Flp-In T-REx 293 cells by Tet. The cells stably transfected with vectors for indicated proteins were stimulated with Tet (1 μg/mL) for the indicated periods of time. Immunoblotting was performed with the indicated antibody. (**c**) Lower expression of TM2D3 in the stably transfected cells as compared to the transiently transfected cells. Flp-In T-REx 293 cells stably transfected with vectors for indicated proteins were stimulated with the indicated dosage of Tet for 20 h. Cell lysates used in **a** were loaded at the left-hand side as controls. Immunoblotting was performed with the indicated antibody. (**d**) Localization of TM2D3-FLAG (red) to vesicular structures in the cells and its slight overlap with the ER marker (green). Flp-In T-REx 293 cells stably transfected with either of the vectors for the indicated proteins or with their empty vector control (Vector) were transiently transfected with a vector for an ER fluorescent marker protein. At 1 day later, cells were stimulated with Tet (1 μg/mL) for 20 h and then fixed. The cells were stained with the indicated antibody and DAPI, and then confocal imaging was performed. The dotted boxes are enlarged in the top right panels. (**e**) Absence of colocalization of TM2D3-FLAG (red) and a Golgi marker (green). This experiment was performed as described in (**d**), except that the cells were transiently transfected with a vector for a fluorescent marker protein for the Golgi.

*TM2D3* has been known to produce two kinds of protein products by alternative splicing, which differ in the presence or absence of an insert of 26 residues near the signal peptide on the carboxy side. The longer and the shorter products are termed variant 1 (v1) and variant 2 (v2), respectively (Supplementary Fig. S1 online). We obtained expression vectors for both forms and transiently transfected them into 293 T cells. A commercially available antibody raised against a peptide nearly corresponding to the portion specific to TM2D3v1 (Supplementary Fig. S1 online) recognized a protein with an apparent molecular mass of 37 kDa from the extract transfected with the vector for TM2D3v1 but not with that for TM2D3v2 or a control empty vector in an immunoblotting analysis (Fig. 1a). We attached a sequence encoding a FLAG tag to those of both the variants in the expression vectors and transiently transfected them into 293 T cells. Figure 1a shows that an anti-FLAG antibody recognized proteins expressed from the vectors. The multiple bands observed in the extracts transfected with TM2D3v1-FLAG in this and other gels and also in those transfected with TM2D3v2-FLAG in other gels may be due to *N*- or *O*-glycosylation, as certain residues of the protein have been predicted by computer-assisted analyses to be the sites for these modifications (Supplementary Fig. S1 online).

In order to assess subcellular localization of the proteins, we established 293-based cell lines that allow the tetracycline (Tet)-inducible expression of each of the tagged proteins from a single recombination site in the genome. Figure 1b shows that the lines express each FLAG-tagged TM2D3 in response to stimulation by Tet. The amount of the proteins induced in each line is about tenfold smaller compared with that of the transiently transfected cells as assessed by a Western blotting analysis (Fig. 1c, Supplementary Fig. S2 online). Confocal analyses of the immunostained cell lines revealed that both tagged TM2D3 proteins localized in small vesicular structures distributed within the cytoplasm (Fig. 1d, e). Most staining did not overlap the endoplasmic reticulum (ER) or the Golgi marker signals (Fig. 1d, e), however, a small portion colocalized with the ER marker (Fig. 1d, insets). These results suggest the localization of the TM2D3 within the cells.

### Overexpression of TM2D3 activates Notch1

**Figure 2:**
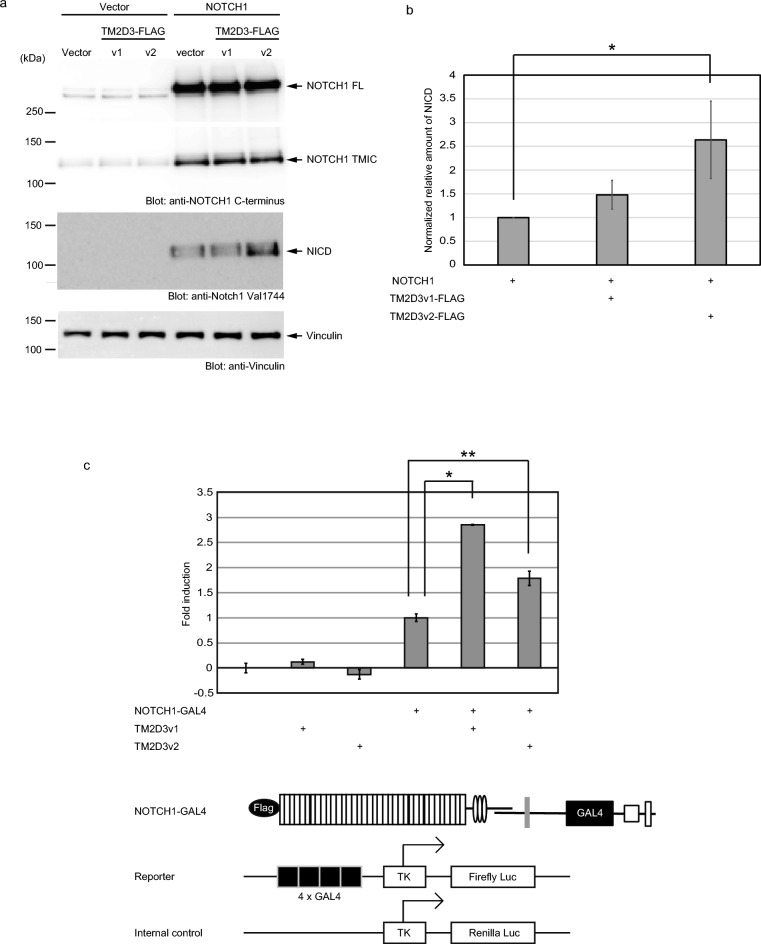
Activation of human NOTCH1 by transient co-transfection of TM2D3. (**a**) Activation of human NOTCH1 by transient co-transfection of a TM2D3v2 construct in 293 T cells. Experiments were conducted as described in Fig. 1a. (**b**) Statistical analysis of the activation of human NOTCH1. Two rounds of independent experiments were additionally conducted as is shown in **a**. The adjusted volume of the NICD band in each extract was normalized to that for vinculin in the corresponding lane. In each experiment, the values in the sample transfected with the vector for NOTCH1 and in empty vector control for TM2D3 were set as 1. The values for the other samples were calculated relative to these amounts. The error bars indicate the standard deviations (n = 3). *F*_(2,6)_ = 8.3; *P* = 1.9 × 10^–2^ using a one-way ANOVA. * P = 1.3 × 10^–2^ by Dunnett’s multiple comparison test. (**c**) Activation of a NOTCH1 construct by co-transfection of TM2D3 in U2OS cells. Cells were transiently transfected with expression vectors for the indicated proteins, a plasmid containing a reporter, and a plasmid containing an internal control. Activation of NOTCH1 receptor was assessed by a luciferase assay. The error bars indicate the standard deviations (n = 3). *P = 6.9 × 10^–5^; **P = 6.7 × 10^–3^ by Dunnett’s multiple comparison test. The NOTCH1 construct, the reporter, and the internal control are indicated schematically below the graph.

To assess the involvement of TM2D3 in the mammalian Notch signaling pathway, we co-transfected the expression vectors for the tagged TM2D3 with that of full-length human NOTCH1 into 293 T cells. We found that expression of the tagged TM2D3v2 enhanced production of the activated form of NOTCH1 (NICD) as assessed by immunoblotting with an antibody specific against Notch1 cleaved by γ-secretase (Fig. 2a). We found no evidence of changes in the expression of the full-length NOTCH1 (NOTCH1 FL) or the form cleaved by furin-like protease at S1 (transmembrane/intracellular fragment; TMIC) by the expression of TM2D3 (Fig. 2a). These results were reproducible in repeated rounds of experiments and the increased production of NICD following co-transfection with the TM2D3v2 construct was statistically significant (Fig. 2b).

We next assessed the activation by a luciferase reporter assay with different cells using a construct of human NOTCH1 of which the intracellular portions required for the association with CSL and Mastermind proteins were swapped with the DNA-binding domain of Gal4^4^ and a reporter with four copies of the yeast UAS enhancer. Figure 2c shows that transient co-transfection of both the forms of untagged TM2D3 with the NOTCH1 construct enhanced the activation of the reporter in U2OS cells. These results are consistent with the notion that overexpression of TM2D3 activates Notch1. Interestingly, for a companion NOTCH1 construct that lacks the cleavage loop of S1 to render it resistant to the processing (a loopout or LO receptor)^4^, the augmentation of activation by the untagged TM2D3 was marginal (Supplementary Fig. S4 online), indicating that the cleavage itself or a surface expression of NOTCH1 that is dependent on the cleavage^4^ is necessary for this activation.

### Overexpression of TM2D3 increases Notch1 expression at the cell surface

To obtain insight into the mechanism(s) of the activation of NOTCH1 by expression of TM2D3, we employed a series of murine Notch1 constructs whose C-terminal domain had been replaced with iterated Myc tags (Supplementary Fig. S5 online)^24^. Consistent with the results shown in Fig. 2a, the transfected Notch1 construct with a full-length extracellular domain (termed Notch1 FL-Myc) was activated by co-transfection of TM2D3v2 in 293 T cells (Supplementary Fig. S6 online). We then examined the effect of ligand stimulation on this activation. Supplementary Fig. S6 online shows that co-culture with control cells had little effect on the activation irrespective of whether TM2D3v2 was transfected. However, Supplementary Fig. S6 online also shows that co-culture with cells overexpressing JAGGED1 ligand upregulated activation of the Notch1 construct either in the absence or presence of co-transfected TM2D3v2.

We next examined responses to known inhibitors of Notch signaling on this activation in the presence of the ligand stimulation. Supplementary Fig. S6 online shows that the activation by TM2D3v2 and the ligand was diminished by a γ-secretase inhibitor (GSI). Supplementary Fig. S6 online also shows that an ADAM inhibitor augmented the activation, a result consistent with a previous report that showed that the activation of transfected but not endogenous Notch was resistant to ADAM inhibitors, probably due to irregular S2 cleavage by an unknown surrogate protease^13^. In this enhanced condition, TM2D3v1 exhibited a weak but noticeable activating effect. Again consistent with the previous report^13^, the GSI inhibits the activation of the Notch construct even in the presence of the ADAM inhibitor. These results are also consistent with the notion that overexpression of TM2D3 activates Notch1 through the canonical pathway.

**Figure 3:**
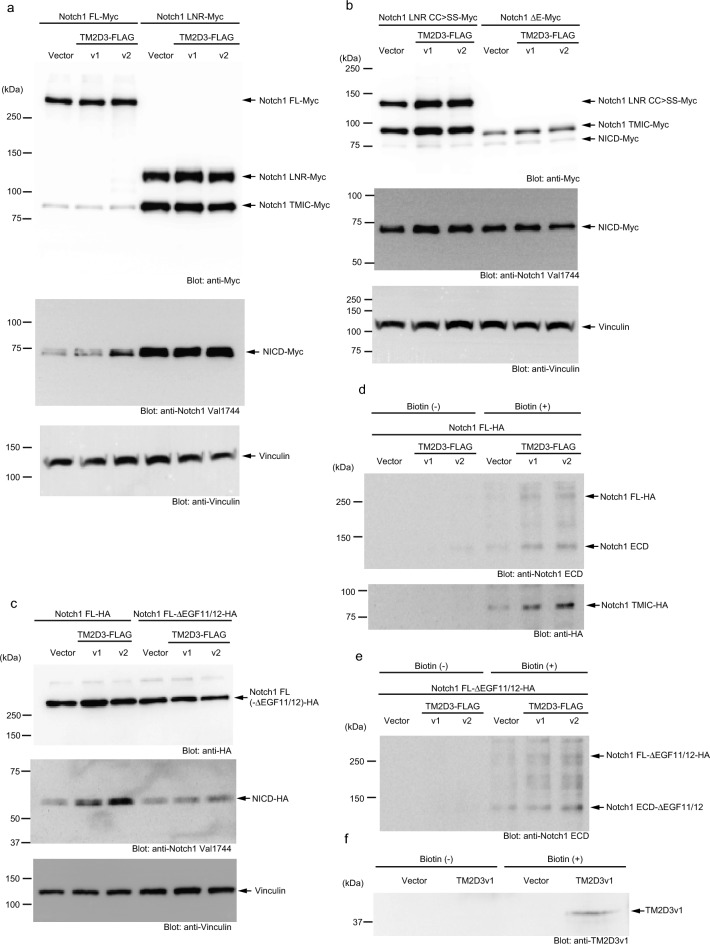
Requirement of the ligand-binding domain of Notch1 for the activation and increased cell surface expression of Notch1 by transient co-transfection of TM2D3. (**a, b**) Activation of Notch1 with a full-length ectodomain but not those that lack EGF repeats (Notch1 LNR, LNR CC > SS, and ΔE) (Supplementary Fig. S5 online) by co-transfection of TM2D3. Cells were transfected with vectors for the indicated proteins or empty vector (Vector) as a control. Immunoblotting was performed with the indicated antibodies. Notch1 LNR CC > SS and ΔE are constitutively active. (**c**) Inability of TM2D3 to activate Notch1 that lacks EGF repeats 11 and 12. Experiments were conducted as in **a** and **b**. (**d, e**) Increased Notch1 expression at cell surface by co-transfection of TM2D3 and its independence on EGF repeats 11 and 12. Cells transfected with vectors for the indicated proteins or empty vector (Vector) as a control were incubated with or without a non-membrane permeable biotinylation reagent as indicated. After fractionation with an avidin agarose, immunoblotting was performed with the indicated antibodies. (**f**) Cell surface expression of TM2D3. Experiments were conducted as in (**d**) and (**e**).

We then utilized deletion mutants of the extracellular domain depicted in Supplementary Fig. S5 online^24^. Figure 3a shows that while the construct with a full-length ectodomain was activated by TM2D3v2, the LNR form that lacks all the EGF repeats and exhibits a higher level of basal activation was not apparently activated further by either variant of TM2D3. Additionally, neither the LNR CC > SS form nor the ΔE form, both of which are constitutively active depending on cleavage(s) by ADAM/γ-secretase or γ-secretase only, respectively^24–26^, were further activated by the transfection of TM2D3 (Fig. 3b). These results indicate that the EGF repeat that contains the ligand-binding domain is necessary for the activation of Notch1 by TM2D3. They also indicate that the proteases are not the apparent targets of TM2D3 during this activation.

We thus wanted to test whether ligand-binding to Notch1 is required for the activation. To this end, we deleted the EGF repeats 11 and 12, which are at the center of the ligand-binding portion^5,6^, from an expression vector of Notch1 with its C-terminal domain swapped with another epitope tag. Figure 3c shows that, while the Notch1 construct with the entire set of EGF repeats was activated by co-transfection of TM2D3, the other construct lacking EGF repeats 11 and 12 was not apparently activated, indicating that this portion is essential for the activation and implying the importance of interaction with the ligands.

We then assessed the roles of maturation and transport of Notch1 by expressing a competitive furin inhibitor AT-EK1^27,28^. Supplementary Fig. S4 online shows that expression of AT-EK1 effectively inhibited the processing of Notch1 to produce the TMIC form. Moreover, the activation of Notch1 by TM2D3 was also inhibited by the expression of AT-EK1. The expression of AT-EK1 did not apparently affect the expression of E-cadherin (Supplementary Fig. S4 online) or either form of TM2D3 (Supplementary Fig. S4 online), implying that the expression of AT-EK1 does not induce a global change to the processing of surface proteins. These results are consistent with the results of the luciferase assay shown in Supplementary Fig. S4 online and again indicate that TM2D3 requires Notch1 to be processed by the convertase and/or to be transported to the plasma membrane^4,27^ for activation.

Based on the above findings, we analyzed the presentation of Notch1 on the cell surface by a surface biotinylation assay. Figure 3d shows that the expression of TM2D3 markedly increased the amount of Notch1 precipitated by streptavidin from the extract of 293 T cells treated with a non-membrane permeable biotinylation reagent. We also found that the amount of biotinylated Notch1 lacking EGF repeats 11 and 12 was also increased by the expression of TM2D3 (Fig. 3e). These results indicate that the expression of TM2D3 increases the presentation of Notch1 at the cell surface and that this presumably also increases the association of Notch1 with ligands expressed by adjacent cells to induce Notch1 activation.

We next examined the expression of TM2D3 on the cell surface by this method, partly because we could not detect TM2D3 by immunostaining without disturbing the plasma membrane with a detergent (Fig. 1d, e). Figure 3f shows that when overexpressed, untagged TM2D3v1 could be biotinylated by the same reagent described above, indicating that TM2D3 can be expressed on the cell surface.

### TM2D3 associates with Notch1 physically

**Figure 4:**
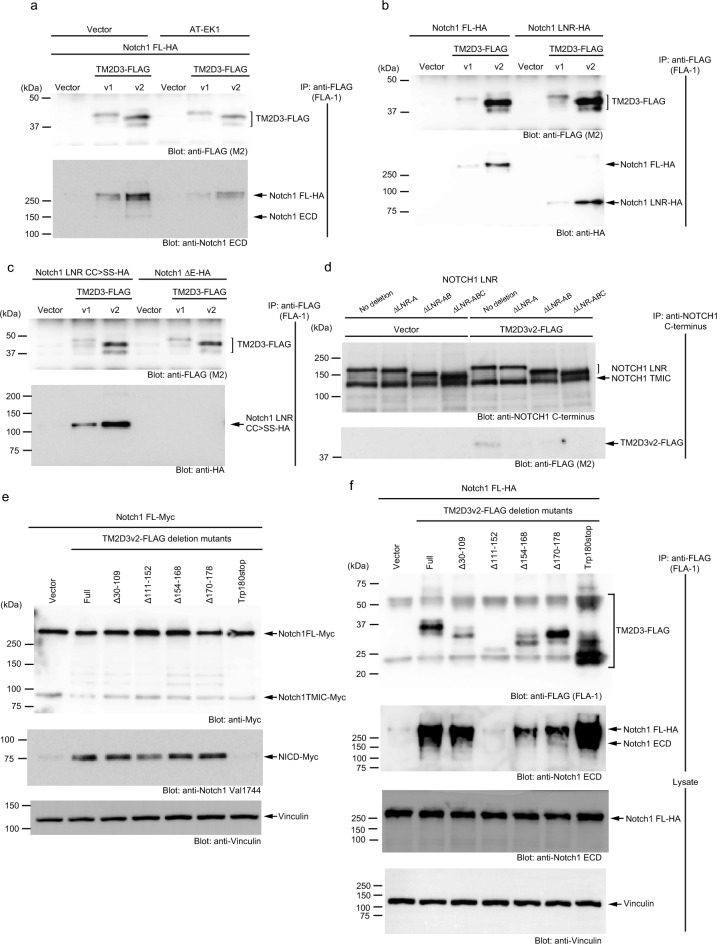
Physical association of Notch1 and TM2D3. (**a**) Physical association of TM2D3 and Notch1. 293 T cells were transfected with vectors for the indicated proteins or empty vector (Vector) as a control. Immunoprecipitation (IP) and immunoblotting were performed with the indicated antibodies. (**b, c**) Requirement of NRR of Notch1 for physical association with TM2D3. Experiments were conducted as in (**a**). (**d**) Requirement of LNR-A of NOTCH1 for physical association with TM2D3. A schematic diagram of NOTCH1 constructs used is shown in Supplementary Fig. S5 online. Experiments were conducted as in **a**. (**e**) Requirement of C-terminal portion including transmembrane 2 domain of TM2D3 for the activation of Notch1. Experiments were conducted as in (**a**). (**f**) No dependency on any one region of TM2D3 for the physical association with Notch1. Experiments were conducted as in (**b**).

These results prompted us to analyze the physical association between the two proteins. A construct containing the entire ectodomain of Notch1 (Supplementary Fig. S5 online) was co-immunoprecipitated with both the variant forms of TM2D3 from cell extracts transfected with the expression vectors (Fig. 4a). As both the nascent form and the form processed at S1 of Notch1 were precipitated, the association appears to begin before the cleavage at TGN and continues after the processing. Supporting this notion, the unprocessed form of Notch1 was also co-immunoprecipitated with TM2D3 even in the presence of AT-EK1 (Fig. 4a).

We next tried to map the portions of Notch1 required for the association with TM2D3 using the deletion mutants of the extracellular domain shown in Supplementary Fig. S5 online. Figure 4b shows that both the variants of TM2D3 were co-immunoprecipitated not only with the full-length form but also with the LNR form. Figure 4c shows that the association was not affected by the presence of CC > SS mutations in the LNR form. However, Fig. 4c also shows that the association was undetectable with the ΔE form, which is devoid of NRR but retains the transmembrane and intracellular domains. These results indicate that NRR of Notch1 is required for the physical association with TM2D3 and that TM2D3 acts in a distinct way from the canonical ligands to activate Notch1.

To further narrow down the portion of Notch1 that is necessary for the association, we employed NOTCH1 constructs with a series of NRR deletions (Supplementary Fig. S5 online)^11^. Figure 4d shows that the form with LNR, HD, TM, and the entire intracellular domain (no deletion) was co-immunoprecipitated with TM2D3v2. When the N-terminal (A) LNR was deleted (termed LNR-A; Supplementary Fig. S5 online), however, the association was greatly reduced. These results may be interpreted to indicate that LNR-A of NOTCH1 is essential for the association. However, the possibility that the absence of the portion disrupts the overall structure of the LNR domain necessary for the association cannot be ruled out.

To obtain insights into the function of TM2D3, we constructed deletion mutants of TM2D3v2 (Supplementary Fig. S9 online), and examined their functions. Supplementary Fig. S9 online shows that all the mutants except for Δ111–152 were expressed in amounts comparable to wild type TM2D3v2 in 293 T cells upon transient transfection. Thus Δ111–152 deletion may disrupt a structure important in stabilizing this protein. When we co-transfected the deletion mutants with the construct containing the entire ectodomain of Notch1, all except for the Trp180stop mutant enhanced the production of the activated form of Notch1 (Fig. 4e). As the Trp180stop mutation created a stop codon just prior to the transmembrane domains, this indicates the requirement of the C-terminal portion including the transmembrane domains for full function. These results are consistent with those of analyses conducted in *Drosophila* that indicated that the *amx*^*1*^ allele, which is indistinguishable from a null allele in terms of the Notch-related phenotype^20^, possesses an eight-nucleotide deletion in the coding region of *amx* gene that introduces a frameshift and a stop codon prior to the transmembrane domains (after amino acid residue 184; Supplementary Fig. S1 online)^19^. Furthermore, Fig. 4f shows that a construct containing the entire ectodomain of Notch1 was co-precipitated with all of the deletion mutants except the unstable Δ111–152. Interestingly, the Trp180stop mutant that was unable to activate Notch1 co-precipitated the largest amount. For this mutant, the stoichiometry of the association with Notch1 may differ from that of the full-length protein and other mutants. Figure 4f also shows that the expression of the mutants of TM2D3 does not apparently affect the expression of Notch1. These results indicate that association itself is not sufficient and the C-terminal region is indispensable for the activation. Further, multiple portions of the extracellular region of TM2D3 may physically associate with Notch1 such that the deletion of one small region is not likely to disrupt the association. Alternatively, Gln^111^—Gln^152^, which is important for protein stabilization, may also be critical for the association.

### Tm2d3 is required for expression of Notch receptors at the cell surface

We next hoped to perform a loss-of-function analysis for TM2D3. In order to accomplish this, we attempted to inactivate the *Tm2d3* gene in a mouse germline using a CRISPR-Cas9 technology. Through this method, we could delete a segment of the gene that contained the exon 5, which encodes a part of the transmembrane domains that had been shown to be required for the activation (Fig. 4e), and that consists of nucleotides whose number is not a multiple of 3. We developed a line of mice that holds this allele (Supplementary Fig. S10 online).

**Figure 5:**
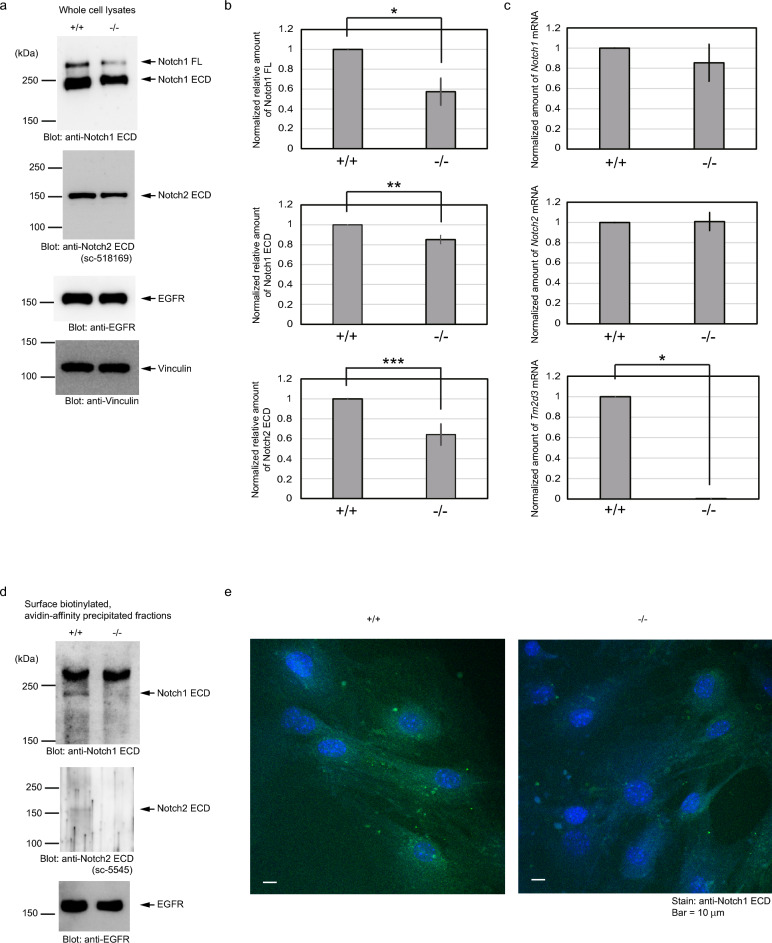
Reduced surface expression of Notch receptors in *Tm2d3*-deficient cells. (**a**) Reduced expression of Notch proteins in *Tm2d3*-deficient cells. Primary cultured embryonic fibroblasts from wild type (+ / +) or *Tm2d3*-deficient (−/−) littermate embryos were analyzed with immunoblotting with the indicated antibodies. (**b**) Reduced expression of Notch1 FL, Notch1 ECD, and Notch2 ECD in *Tm2d3*-deficient cells. Two rounds of independent experiments were additionally conducted as shown in (**a**). Adjusted volumes for the bands for Notch1 FL, Notch1 ECD, and Notch2 ECD in each extract were normalized to that of the vinculin band in the corresponding lane. In each experiment, the value in the + / + cells was set as 1, and the value for the −/− cells was calculated relative to this amount. The error bars indicate the standard deviations (n = 3). *P = 3.2 × 10^–2^ using Welch’s *t*-test. **P = 2.4 × 10^–2^ using Welch’s *t*-test. ***P = 2.8 × 10^–2^ using Welch’s *t*-test. Notch2 FL was barely detectable in all the three rounds of experiments. (**c**) mRNA expression levels for *Notch1*, *Notch2*, and *Tm2d3* in *Tm2d3*-deficient cells. Three sets of the primary cultured embryonic fibroblasts of the indicated genotypes from littermate embryos were subjected to RNA isolation and real-time PCR analysis using the indicated primers. As the calculations shown in **c**, the value in the + / + cells was set as 1, and the value of the −/− cells was calculated relative to this amount in each of the sets. The error bars indicate the standard deviations (n = 3). *P = 7.0 × 10^–8^ using Welch’s *t*-test. (**d**) Reduced surface biotinylation of Notch receptors in *Tm2d3*-deficient cells. Primary cultured embryonic fibroblasts of the either genotype ([+ / +], [−/−]) isolated from littermate embryos were incubated with a non-membrane permeable biotinylation reagent. Immunoblotting was done for proteins fractionated with an avidin agarose with the indicated antibodies. In the anti-Notch1 ECD blot, the band above 250 kDa is non-specific. Notch1 FL is not efficiently transported to the cell surface and biotinylated. As the anti-Notch2 ECD antibody (sc-518169) did not clearly react with the biotinylated antigen, another antibody against Notch2 ECD (sc-5545) was used for this analysis. The murine epitope of the sc-518169 antibody contains a lysine residue (Lys^268^) that can be biotinylated. (**e**) Reduced surface immunoreactivity of Notch1 ECD in *Tm2d3*-deficient cells. Primary cultured embryonic fibroblasts of either genotype ([+ / +], [−/−]) isolated from littermate embryos were incubated with the indicated anti-Notch1 antibody followed by fixation, visualization by secondary antibodies, and confocal microscopy. Vesicles stained represent endocytic vesicles that contain the first antibody, as the incubation with the first antibody was performed at room temperature (see Methods).

In order to analyze an effect of the inactivation of *Tm2d3* on expression of Notch receptors, we prepared primary cultures of embryonic fibroblasts from mid-gestation embryos generated by intercrossing the heterozygous mice. Western blotting analyses of the whole cell lysate showed that expression of both Notch1 and Notch2 was reduced to a certain extent in the cells that were homozygous for the mutant allele of *Tm2d3* compared with the wild type cells (Fig. 5a). In contrast, expression of both the EGF receptor (EGFR) and vinculin was not apparently different between the cells. The reduced expression was also evident in two other pairs of independently prepared primary fibroblast cultures, and the average rates of the reduction were calculated to be approximately 40% and 20% for the full-length form and the ECD of Notch1, respectively, and 40% for the ECD of Notch2 taking the expression of vinculin as an internal standard (Fig. 5b).

We then quantitated *Notch1* and *Notch2* cellular mRNAs. In contrast with the proteins, consistent difference was not observed in the mRNA expression between the two genotypes (Fig. 5c). Expression of *Tm2d3* mRNA was disrupted in accordance with the genotype (Fig. 5c).

However, when we performed the surface biotinylation assay, we found that the amount of the biotinylated ECD from both Notch1 and Notch2 was greatly reduced in homozygously deleted cells compared with the wild type cells, while biotinylated EGFR was not notably different between the two types of cells (Fig. 5d). These results indicated that Tm2d3 is required for the efficient surface presentation of both Notch1 and Notch2 receptors in addition to the effects on expression.

We also assessed the cell surface expression of Notch1 in these cells by another method. The live cells were incubated with an antibody against the ECD for a short period of time. After the incubation and wash, the cells were fixed and the antibody was visualized with a secondary antibody conjugated with a fluorescent dye. Figure 5e shows that the stain was reduced in the homozygous cells as compared to the wild type cells. These results again indicate that *Tm2d3*-deficiency reduces cell surface expression of Notch1.

### amx-deficient early Drosophila embryos exhibit impairment of the endocytosis of Notch extracellular domain and Delta in presumptive mesodermal cells.

**Figure 6:**
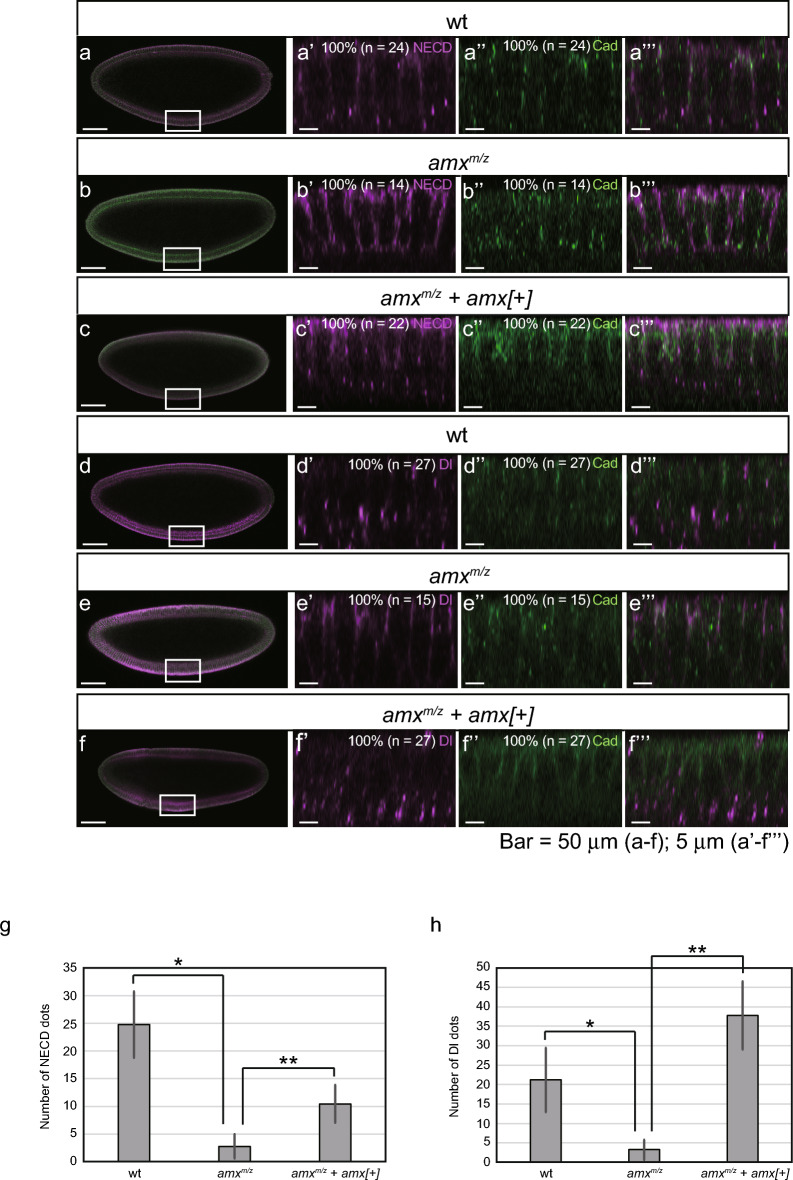
Decreased NECD- and Dl-containing endocytic vesicles in ventral presumptive mesodermal cells of *amx*-deficient early *Drosophila* embryos. (**a**–**f**) Decrease of NECD- and Dl-containing dots in the basal region of the ventral mesodermal cells and its restoration by a genetic rescue. Wild type (wt), *amx*^*m/z*^, and *amx*^*m/z*^ + *amx[* +*]* embryos at cellular blastoderm stage (stage 5) were stained with an anti-NECD antibody (NECD: magenta) or an anti-Dl antibody (Dl: magenta). Co-staining with an anti-*Drosophila* E-cadherin antibody (Cad: green) was performed to show the adherence junctions of the cells and to control the localization of a cell surface protein. (**a**–**f**) Lateral views of the embryos. Left: anterior, right: posterior, upper: dorsal, bottom: ventral. (**a’**–**f’’**) High-magnification images of ventral mesodermal cells (the regions indicated by boxes in a-f). Upper: apical, bottom: basal. n: numbers of embryos assessed. All embryos showed a constant phenotype (100%). (**a’’’**–**f’’’**) Merged images of the two staining modalities depicted in the two middle panels. (**g**) Decrease of NECD-containing dots in the basal region of *amx*-deficient ventral mesodermal cells and its restoration by a genetic rescue. Number of dots as exemplified in g-i were counted. For each category, three embryos were analyzed. For each embryo, three pictures were analyzed. The error bars indicate the standard deviations (n = 9). *F*_(2,24)_ = 64.5, *P* = 2.2 × 10^–10^ using a one-way ANOVA. *P = 8.1 × 10^–9^; **P = 1.3 × 10^–3^ by Dunnett’s multiple comparison test. (**h**) Decrease of Dl-containing dots in the basal region of *amx*-deficient ventral mesodermal cells and its restoration by a genetic rescue. Analysis was conducted as in (**j**). The error bars indicate the standard deviations (n = 9). *F*_(2,24)_ = 53.2, *P* = 1.5 × 10^–9^ using a one-way ANOVA. *P = 3.2 × 10^–5^; **P = 8.5 × 10^–9^ by Dunnett’s multiple comparison test.

In order to assess the consequences of the loss of function in additional in vivo settings, we turned to *Drosophila amx*, the homolog of *TM2D3*. For this purpose, we employed the classical *amx*^*1*^^17–19,22^ allele, which is indistinguishable from a null allele in terms of Notch-related phenotypes^20^. In wild type *Drosophila* embryos, the expression of Notch begins at the surface of cells at the cellular blastoderm stage (stage 5) and that of Delta (Dl) begins at the cortical membrane of the precellular blastoderm stage (stage 4) and within invaginating cell membranes as cellularization proceeds (stage 5)^29–33^. In simple columnar epithelial cells of the ventral presumptive mesoderm at the late stage 5, the receptor and the ligand appear to interact. These cells hold endocytic vesicles, which contain both the epitopes of Notch ECD (NECD) and Dl, in the basal half of the cells^9,30,31,34^. Consistent with these previous results, both NECD and Dl were found in intracellular dots as well as at the plasma membrane in the wild type embryos (Fig. 6a-a’’’, d-d’’’). In contrast, in the *amx*-deficient mutant embryos (*amx*^*m/z*^), the basal dots were scarcely observed, while both NECD and Dl accumulated around the plasma membrane in the apical region (Fig. 6b-b’’’, e-e’’’). Counting confirmed the paucity of the dots in the *amx*-deficient mutants for either NECD (Fig. 6g, Supplementary Fig. S12 online) or Dl (Fig. 6h, Supplementary Fig. S12 online). Quantitation of the localization of NECD and Dl in the cells also showed that the *amx*-deficiency caused a decrease in both the epitopes in the basal half of the cells and an increase in the apical region (Supplementary Fig. S13 online). In contrast, the localization of E-cadherin (Cad) clearly did not differ between the genotypes (Fig. 6a’’, b’’, d’’, e’’, and Supplementary Fig. S13 online), indicating that *amx*-defficiency did not cause global perturbation of the trafficking.

We then assessed the consequences of a genetic rescue by a genomic construct (*amx[* +*]*), which had been shown to fully rescue the neurogenic phenotype caused by the *amx*^*1*^ allele^22^. As shown in Fig. 6c-c’’’, f-f’’’, g, h, and Supplementary Fig. S12 online, both the NECD dots and Dl dots were restored by introduction of the construct into both the maternal and zygotic genome by crossing. Of note, Fig. 6c-c’’’ also shows that a considerable amount of the NECD stain remained in the apical region, while most Dl stain was found as the dots in the cells (Fig. 6f-f’’’). As a result, the quantitation presented in Supplementary Fig. S13 online shows the restoration of Dl but not of NECD to a wild type-like distribution by this rescue. These results indicate that the deficiency of *amx* is responsible for the paucity of the endocytic dots and suggest that *amx*-deficiency causes impairment of the endocytosis of NECD and Dl subsequent to the ligand-receptor interaction. Although various possibilities such as *amx*-deficiency affects endocytosis itself cannot be ruled out, these results are consistent with the notion that Amx is required for the surface presentation of Notch, which is a prerequisite for its interaction with Dl leading to endocytosis to either direction^9^.

## Discussion

In this study we have examined TM2D3 and clarified its possible functions. Overexpression of TM2D3 activates Notch1 and TM2D3 physically associates with Notch1. In Notch1, this association requires the extracellular NRR but not the EGF repeats. In TM2D3, the N-terminal extracellular domain is responsible for the association. As the C-terminal region of TM2D3 including the dual transmembrane domain is dispensable for the association but required for the activation, the association is not sufficient for the activation. In Notch1, the ligand-binding portion (EGF repeats 11 and 12) is necessary for the activation. Ligands expressed in neighboring cells are thus supposed to play a role in the activation. Indeed, it is known that such ligands, especially JAGGED1, are expressed in 293 T cells^35^. Consistent with this notion, the overexpression of TM2D3 increases the surface presentation of Notch1, making the receptor accessible to the ligands.

Two variant forms of TM2D3 protein generated by alternative splicing are known. Among them, the smaller variant 2 constantly activates Notch1 in the overexpression studies using 293 T cells. However, in some experiments, especially in the presence of enhancers (Supplementary Fig. S6 online), the larger variant 1 also exhibits a weaker but confirmed activation of Notch1 in 293 T cells. Importantly, the overexpressed variant 1 also causes an enhanced surface presentation of Notch1 in 293 T cells, although more weakly than that caused by variant 2. This correlation between the strength of activation and the enhancement of surface presentation of Notch1 by the two forms is more evidence that the activation of Notch1 by TM2D3 is caused through the augmentation of the surface presentation of Notch1.

In *Tm2d3*-deficient cells, the cell surface expression of not only Notch1 but also Notch2 is reduced. The mechanisms appear to be twofold: the reduced expression of the proteins themselves and the reduced rate of the surface-presented receptors to the expressed proteins. The reduced rate of the cell-surface presentation complements the overexpression studies mentioned above. In contrast, there is no evidence throughout the study that the overexpression of TM2D3 in 293 T cells causes the increased expression of Notch1 (Figs. 2a, 3a-c, and 4e-f). This difference indicates that the two functions are distinct and that the endogenously expressed amount of TM2D3 in the cells is large enough to saturate the first process, augmentation of the protein expression, but is small enough to leave a place for the second process, enhancement of the cell-surface presentation.

In *Tm2d3*-deficient cells, expression of both the full-length and the S1-processed forms of Notch1 is reduced. These results may be consistent with the result that both the forms of the Notch1 construct can be co-immunoprecipitated with the tagged TM2D3 (Fig. 4a), and with the idea that Notch1 protein stabilization is related to the physical association between these proteins. However, we cannot rule out the possibility that the effect of *Tm2d3* deficiency is at the translational level.

Experiments involving a surface labeling and an immunocytochemical analysis suggest that TM2D3 localizes both at the cell surface and in intracellular vesicular structures, a small portion of which colocalizes with an ER marker, but for which the major location(s) is/are currently unknown. The molecular mechanism(s) of action of TM2D3 is/are also unclear and need to be investigated.

Furthermore, in *Drosophila* embryonic presumptive mesodermal cells, *amx*-deficiency causes a decrease in intracellular dots. These results are consistent with a blockade during ligand-receptor interaction and the subsequent endocytosis. In the mutant cells, both NECD and Dl are accumulated around the apical plasma membrane. We have tried to identify their localization by co-staining with various markers. However, not an anti-GM130 (a cis-Golgi marker), an anti-Rab5 (an early endosome marker), an anti-Rab7 (a late endosome marker), or anti-Rab11 (a recycling endosome marker) antibodies or a peanut agglutinin that stains middle- and trans-Golgi and plasma membrane notably co-stained with either NECD or Dl (data not shown). When protein disulfide isomerase (an ER marker)-GFP fusion protein was expressed, an anti-GFP antibody did not co-stain with either NECD or Dl as well (data not shown). These results suggest that both NECD and Dl in the *amx*-deficient cells are accumulated not in these components of the secretory and endocytic pathways but in somewhere else that remains to be clarified.

Introduction of the genomic rescue construct, which encompasses an ~ 3.3 kb region containing the wild type *amx* gene inserted in the 2nd chromosome, results in restoration of the neurogenic phenotype^22^ and paucity of NECD- and/or Dl-containing dots but not of the localization of NECD in the plasma membrane region of the *amx* mutant (Supplementary Fig. S13 online). This partial rescue could be due to a lower expression of *amx* from the rescue construct compared to the gene in the wild type settings. A remote enhancer(s) may be lost in the construct and/or the construct may be inserted at a chromosomal position influenced by an operator(s). Nevertheless, a correlation between the presence of the dots and the absence of the neurogenic phenotype that indicates a loss of Notch signaling is consistent with the idea that the dots are the result of the endocytosis that is involved in the signaling. The introduction also causes a small aberrant distribution of the Cad epitope (Supplementary Fig. S13 online), for unknown reasons.

Although we do not know at this moment whether TM2D3 physiologically works as a regulatory element to modify the surface presentation of Notch, it would be an interesting possibility. The stabilization of a receptor at the cell surface could be a unique mechanism of action to control intercellular signaling, since such a regulatory mechanism would be most operative in a pathway in a manner such as Notch signaling, which is free from enzymatic amplification but relies on stoichiometric interactions among the elements of signaling^2,7^.

## Methods

### Plasmids

Human cDNAs for TM2D3v1 and v2 were obtained from OriGene. The cDNAs were cloned in a pCMV6-XL5 expression vector (OriGene) for transient transfection. To express N-terminally FLAG-tagged proteins, the region encoding most of the signal peptide (from Met^1^ to Cys^25^) (Supplementary Fig. S1 online) from each of the vectors was replaced with a sequence encoding a FLAG tag preceded by a signal peptide sequence of preprotrypsin from pFLAG-CMV-1 (Sigma-Aldrich). For stable transfection, each of the coding regions of the tagged TM2D3 proteins was cloned in pcDNA5⁄FRT⁄TO (Thermo Fisher Scientific). pEYFP-ER and pEYFP-Golgi were obtained from BD Biosciences. Full-length human NOTCH1 cDNA cloned in pcDNA1/Amp (Invitrogen) were as described^36^. A chimeric human NOTCH1 construct in which the RAM-ANK domain had been replaced with the DNA-binding domain of Gal4 (NOTCH1-GAL4) (Fig. 2c) was as described^4^. A firefly luciferase reporter with four copies of the yeast UAS enhancer and a thymidine kinase promoter from the herpes simplex virus (TK-MH100 × 4-LUC) (Fig. 2c) were as described^37^. An internal control vector for the luciferase assay, pRL-TK (Fig. 2c), was from Promega. A series of murine Notch1 constructs with deletions and point mutations in the ectodomain, and replacement of the PEST domain with 6 Myc tags, pCS2 + Notch1 FL-6MT, pCS2 + Notch1 LNR, pCS2 + Notch1 LNR CC > SS, and pCS2 + Notch1 ΔE (Supplementary Fig. S5 online) were as described^24^. To avoid non-specific binding to protein G-Sepharose and avidin agarose, the portion encoding the 6 Myc epitope tags of the vectors was replaced with a sequence encoding a single HA epitope tag (pCS2 + Notch1 FL-HA, pCS2 + Notch1 LNR-HA, pCS2 + Notch1 LNR-HA CC > SS, and pCS2 + Notch1 ΔE-HA) (Supplementary Fig. S5 online). An expression vector for the mutant of Notch1 with deletion of the EGF repeats 11 and 12 (from Val^413^ to Val^485^; Notch1 FL-ΔEGF11/12-HA) and vectors for deletion mutants of TM2D3 (Supplementary Fig. S9 online) were constructed by digestion with appropriate restriction endonucleases, filling in by T4 DNA polymerase, and ligation of pCS2 + Notch1 FL-HA and pCMV6-XL5 TM2D3v2, respectively. An expression vector for a variant of α_1_-antitrypsin, AT-EK1, which is a strong competitive furin inhibitor in pcDNA3, was as described^38^. NOTCH1 mutants with a series of deletions in LNR (Supplementary Fig. S5 online) were as described^11^.

### Antibodies and reagents

An antibody for TM2D3v1 was purchased from Abgent or Sigma-Aldrich. Antibodies for FLAG epitope (DDDDK or DYKDDDDK) tag from Sigma-Aldrich (M2; F3165), MBL (FLA-1; M185-3), and Proteintech (2B3C4; 66008–3-Ig) were used as indicated. An antibody against the C-terminus of human NOTCH1 (sc-6014), an antibody against the ECD of murine Notch1 (8G10; sc-32756), and antibodies against the ECD of Notch2 (sc-518169 and sc-5545) were obtained from Santa Cruz Biotechnology. An antibody against cleaved Notch1 (Val1744) (D3B8; #4147), an antibody against vinculin (E1E9V; #13901), and an antibody against EGFR (D38B1; #4267) were obtained from Cell Signaling Technology. An anti-Myc tag antibody (9E10) and an anti-HA antibody (12CA5) were prepared in-house from hybridomas.

### Cell culture and transfection

293 T cells were maintained in Dulbecco’s modified Eagle’s medium (DMEM) containing fetal bovine serum (FBS) (10%). Except for a surface biotinylation assay, the cells (6.6 × 10^5^ cells/6-cm dish) were transiently transfected with plasmid DNA (10 μg) by Lipofectamine 2000 (Thermo Fisher Scientific) (for experiments presented in Figs. 1a and 2a, b) or by the calcium phosphate method. For co-transfection involving Notch1 (NOTCH1) and TM2D3, a Notch1-related plasmid (6 μg) and a TM2D3-related plasmid (4 μg) were used. For co-transfection involving AT-EK1, a Notch1-related plasmid (5 μg), a TM2D3-related plasmid (3 μg), and an AT-EK1-related plasmid (2 μg) were used. Protein extracts were obtained as described^39^ except that the incubation time after the transfection was changed to 20 h for experiments to detect TM2D3 proteins.

Flp-In T-REx 293 cells (Thermo Fisher Scientific) that express Tet repressor and contain a single FRT site in the genome were maintained in DMEM containing FBS (10%), Blasticidin S (Thermo Fisher Scientific) (10 μg/mL), and Zeocin (Thermo Fisher Scientific) (1 mg/mL). They were transfected with a pcDNA5⁄FRT⁄TO vector containing either of the FLAG-tagged TM2D3 or their empty counterpart together with pOG44 (Thermo Fisher Scientific) that expresses Flp recombinase using Lipofectamine 2000. Stably transfected cells were selected and grown in DMEM containing FBS (10%), Blasticidin S (10 μg/mL), and Hygromycin B (Thermo Fisher Scientific) (125 μg/mL).

Targeting of *Tm2d3* gene in the mouse genome and culture of primary cultured embryonic fibroblasts were described in Supplementary Fig. S10 online and Supplementary Methods online.

### Immunoblotting and immunoprecipitation

Immunoblotting and immunoprecipitation were performed essentially as described previously^39^. To analyze TM2D3 by immunoblotting, samples were denatured at 50 °C for 5 min before loading to the gels. The first antibodies on the membrane filters were visualized with secondary antibodies conjugated with horseradish peroxidase, a Western Lightning ECL Pro (PerkinElmer) chemiluminescent substrate, and a LAS-1000plus (Fujifilm) or ChemiDoc Touch (Bio-Rad) luminescent image analyzer; or secondary antibodies conjugated with near infrared dyes and Odyssey CLx (LI-COR).

### Quantitation of the Western blot signals

To quantitate the NICD signals shown in Fig. 2a, images were accessed using ImageJ^40^ software and converted to pseudocolor images. The regions of interest (ROIs) of equal areas were selected and the mean gray values for the ROIs were measured. To quantitate the band signals observed in the additional experiments to Fig. 2a and the experiments shown in Fig. 5, images were accessed using ImageLab Touch software on the ChemiDoc Touch Imaging system (Bio-Rad).

### Immunocytochemistry

Flp-In T-REx 293 cells stably transfected with either the empty vector, the expression vector for TM2D3v1, or the expression vector for TM2D3v2 were transiently transfected with either pEYFP-ER (an expression vector for enhanced yellow fluorescent protein (EYFP) fused to the ER targeting sequence of calreticulin) or pEYFP-Golgi (an expression vector for EYFP fused to the Golgi targeting sequence of β1,4-galactosyltransferase) using Lipofectamine 2000. At 1 day later, the cells were seeded on glass coverslips. One day later, the medium was replaced with that containing Tet (1 μg/mL). Twenty hours after the replacement, cells were fixed with paraformaldehyde (4%) in phosphate buffered saline (PBS) for 10 min at room temperature. Following washing, cells were permeabilized with Saponin (0.05%) in PBS for 5 min at room temperature followed by washing and blocking with PBS containing bovine serum albumin (BSA) (10%) for overnight at 4 °C. The cells were incubated with an anti-FLAG antibody (0.625 μg/mL) diluted in PBS containing BSA (3%) for 30 min at room temperature. After washing and a 45-min incubation with a Cy3-conjugated anti-mouse Ig secondary antibody (Jackson ImmunoResearch) (1:500) diluted in PBS containing BSA (3%) at room temperature and wash with PBS, the cells were mounted with SlowFade Diamond Antifade Mountant with DAPI (4',6-diamidino-2-phenylindole) (Thermo Fisher Scientific).

Primary cultured embryonic fibroblasts were seeded on a glass cover slip. One day later, they were incubated with the growth medium containing the anti-Notch1 ECD (4 μg/mL) at room temperature for 30 min. After the staining, they were washed with PBS containing CaCl_2_ (1 mM) and MgCl_2_ (0.5 mM) and fixed with paraformaldehyde (4%) in PBS for 15 min. After a 2-h incubation with either Alexa 488-conjugated goat anti-hamster secondary antibody (Thermo Fisher Scientific) (4 μg/mL) at room temperature and wash, the cells were mounted with SlowFade Diamond Antifade Mountant with DAPI.

Confocal microscopy was performed using an LSM880 system (Zeiss). Cy3, DAPI, EYFP, and Alexa 488 signals were scanned individually. Images were analyzed with the ZEN imaging software (Zeiss). For each experiment, a control experiment was conducted in the same manner except that the primary antibody was excluded. In each case, few signals were observed, indicating that the signals were dependent on the primary antibodies.

### Luciferase assay

U2OS cells were maintained in DMEM containing 10% FBS. The cells seeded in 12-well dishes (0.9 × 10^5^ cells/well) were co-transfected with a Notch-related plasmid (0.2 μg), a TM2D3-related plasmid (0.2 μg), the firefly luciferase reporter plasmid (0.4 μg), and the *Renilla* luciferase internal control plasmid (0.4 × 10^–2^ μg). The transient transfection was performed using Lipofectamine 2000. Twenty-four hours after the transfection, firefly and *Renilla* luciferase activities were determined using a dual luciferase assay kit (Promega) and a Turner Designs TD20/20 dual luminometer. Firefly luciferase activities were normalized with the *Renilla* luciferase control activities.

### Surface biotinylation assay

Biotinylation of cell surface proteins and isolation with streptavidin beads were achieved using a Pierce Cell Surface Protein Isolation Kit (Thermo Fisher Scientific). 293 T cells (2 × 10^6^ cells/10-cm dish) were transfected with the indicated plasmid DNA (30 μg) by the calcium phosphate method. For co-transfection, a Notch1-related plasmid (18 μg) and a TM2D3-related plasmid (12 μg) were used. The transfected cells were biotinylated or left untreated. Primary cultured embryonic fibroblasts (four confluent 10-cm dishes) were also biotinylated by the kit. Biotinylated proteins in lysates of the cells were isolated with NeutrAvidin agarose. Proteins eluted from the resin were analyzed by immunoblotting (1/10 per well).

### Quantitation of mRNA

For the real-time PCR analysis, total RNA was isolated using TRIzol reagent (Invitrogen). Reverse transcription of the RNA was carried out using SuperScript IV Reverse transcriptase (Thermo Fisher Scientific). Real-time PCR was performed using the Fast SYBR Green Master Mix kit (Thermo Fisher Scientific) and the 7500 Fast Real-Time PCR System (Applied Biosystems). The mRNA expression of glyceraldehyde-3-phosphate dehydrogenase (*Gapdh*) was used as an internal control. Primer sequences were,

**Table:** 

	Forward (5’ to 3’)	Reverse (5’ to 3’)
*Gapdh*	GCACCGTCAAGGCTGAGAAC	TGGTGAAGACGCCAGTGGA
*Notch1*	GCTGCCTCTTTGATGGCTTCGA	CACATTCGGCACTGTTACAGCC
*Notch2*	CCACCTGCAATGACTTCATCGG	TCGATGCAGGTGCCTCCATTCT
*Tm2d3*	GCTATTTCGCCAACTGCACCGT	TCGACCACTTGTAGCCACCTGT

### Drosophila experiments

All stocks were maintained and all crosses were performed at 25 °C on standard *Drosophila* culture medium. Canton-S was used as wild type. A loss-of-function mutant of *amx*, namely, *amx*^*1*^* lz*^*g*^* v*^*1*^/*C(1)DX, y*^*1*^*, f*^*1*^ (Bloomington Drosophila Stock Center (BDSC) #10)^41^, and *Df (1)BSC663*/FM7h (BDSC #26,515) were obtained from BDSC. The maternal and zygotic *amx*-deficient mutant embryos (*amx*^*m/z*^) were obtained by the following genetic crosses. *amx*^*1*^* lz*^*g*^* v*^*1*^/Y males were crossed with *Df (1)BSC663*/FM7h virgin females. In the next generation, *amx*^*1*^* lz*^*g*^* v*^*1*^/*Df (1)BSC663* virgin females were collected and crossed with *amx*^*1*^* lz*^*g*^* v*^*1*^/Y males. For rescue experiments, an allele holding a genomic fragment encompassing the wild type *amx* locus in the 2nd chromosome (*amx[* +*]*)^22^ was used. Namely, *amx*^*1*^* lz*^*g*^* v*^*1*^/*Df (1)BSC663*; *amx[* +*]/* + virgin females were crossed with *amx*^*1*^* lz*^*g*^* v*^*1*^*/Y; amx[* +*]/ CyO, Kr-GFP* males. The resultant embryos that did not express GFP were analyzed. Fly crosses were allowed to lay eggs for 3.5 h on a grape agar plate. Embryos were collected, fixed, and preserved using standard procedures^42^. The egg collection was repeated two or three times a day from 10 a.m. for two days. Primary antibodies used for staining were a mouse monoclonal anti-Notch extracellular domain (C458.2H; Developmental Studies Hybridoma Bank [DSHB]) (1:500), a mouse monoclonal anti-Delta (C594.9B; DSHB) (1:500), and a rat anti-*Drosophila* E-cadherin (DCAD2; DSHB) (1:20). Fluorescent secondary antibodies used were a Cy3-conjugated anti-mouse IgG (Jackson ImmunoResearch) (1:500) and an Alexa Fluor 488-conjugated anti-rat IgG (Molecular Probes) (1:500). Confocal microscopy was performed using an LSM770 system (Zeiss) and ZEN imaging software. All the embryos judged to be at the cellular blastoderm stage (stage 5) and with an appropriate genotype (in the case of *amx*^*m/z*^ + *amx[* +*]* embryos) were analyzed. Experiments involving the wild type and *amx*^*m/z*^ embryos were conducted side-by-side for three times independently. Experiments involving the *amx*^*m/z*^ + *amx[* +*]* embryos were conducted later under the same conditions for three times independently. Flies were anesthetized with carbon dioxide followed by euthanasia with 75% ethanol.

To quantitate the fluorescent signals, z-stack images were accessed with ImageJ^40^. They were first converted to pseudocolor images with the fire lookup table. Dots that reside in the bottom half of the cells and that do not apparently look like parts of the plasma membrane were counted manually.

Blinding was not used due to the personnel constraints. The study was carried out in compliance with the ARRIVE guidelines (https://arriveguidelines.org).

### Statistical analysis

All statistics were performed using IBM SPSS statistics v. 29. Data were tested for a one-way analysis of variance (ANOVA), Dunnett’s multiple comparison test, and Welch's *t*-test.

### Supplementary Information

Supplementary Information 1.

Supplementary Information 2.

Supplementary Information 3.

Supplementary Information 4.

Supplementary Information 5.

Supplementary Information 6.

Supplementary Information 7.

Supplementary Information 8.

Supplementary Information 9.

Supplementary Information 10.

Supplementary Information 11.

Supplementary Information 12.

Supplementary Information 13.

Supplementary Information 14.

Supplementary Information 15.

Supplementary Information 16.

Supplementary Information 17.

Supplementary Information 18.

Supplementary Information 19.

Supplementary Information 20.

Supplementary Information 21.

Supplementary Information 22.

Supplementary Information 23.

Supplementary Information 24.

Supplementary Information 25.

Supplementary Information 26.

Supplementary Information 27.

Supplementary Information 28.

## Data Availability

Correspondence and requests for materials should be addressed to the corresponding author, Dr. Motoo Kitagawa (e-mail: kitagawa@iuhw.ac.jp).
