# Supplementary figures and images for: TM2D3, a mammalian homologue of Drosophila neurogenic gene product Almondex, regulates surface presentation of Notch receptors
Source: Sci Rep. 2023 Nov 27;13:20913. doi: 10.1038/s41598-023-46866-7 (PMC10684865; doi:10.1038/s41598-023-46866-7)

# Supplementary Figure S1

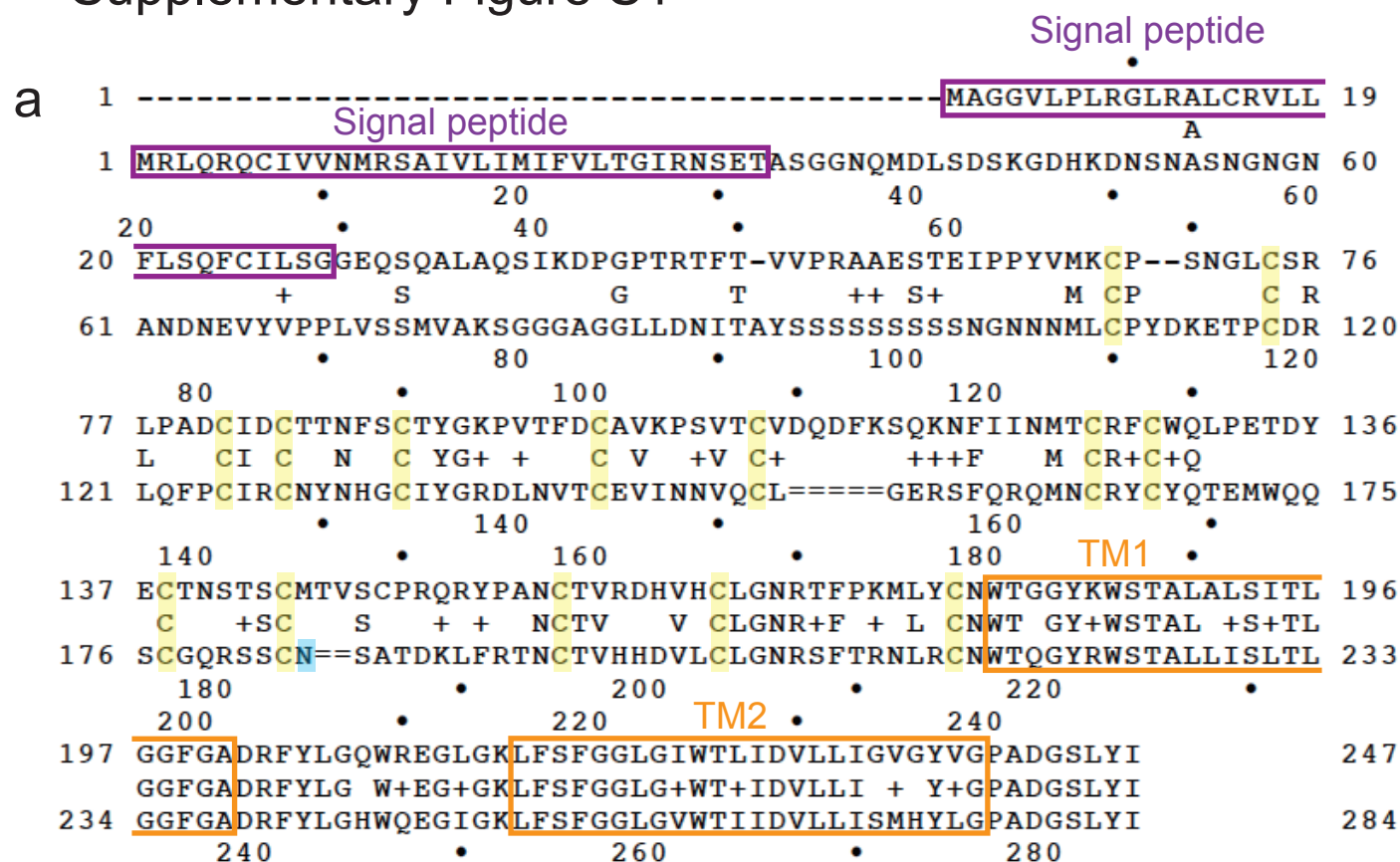

Upper : TM2D3v1

Lower : Amx

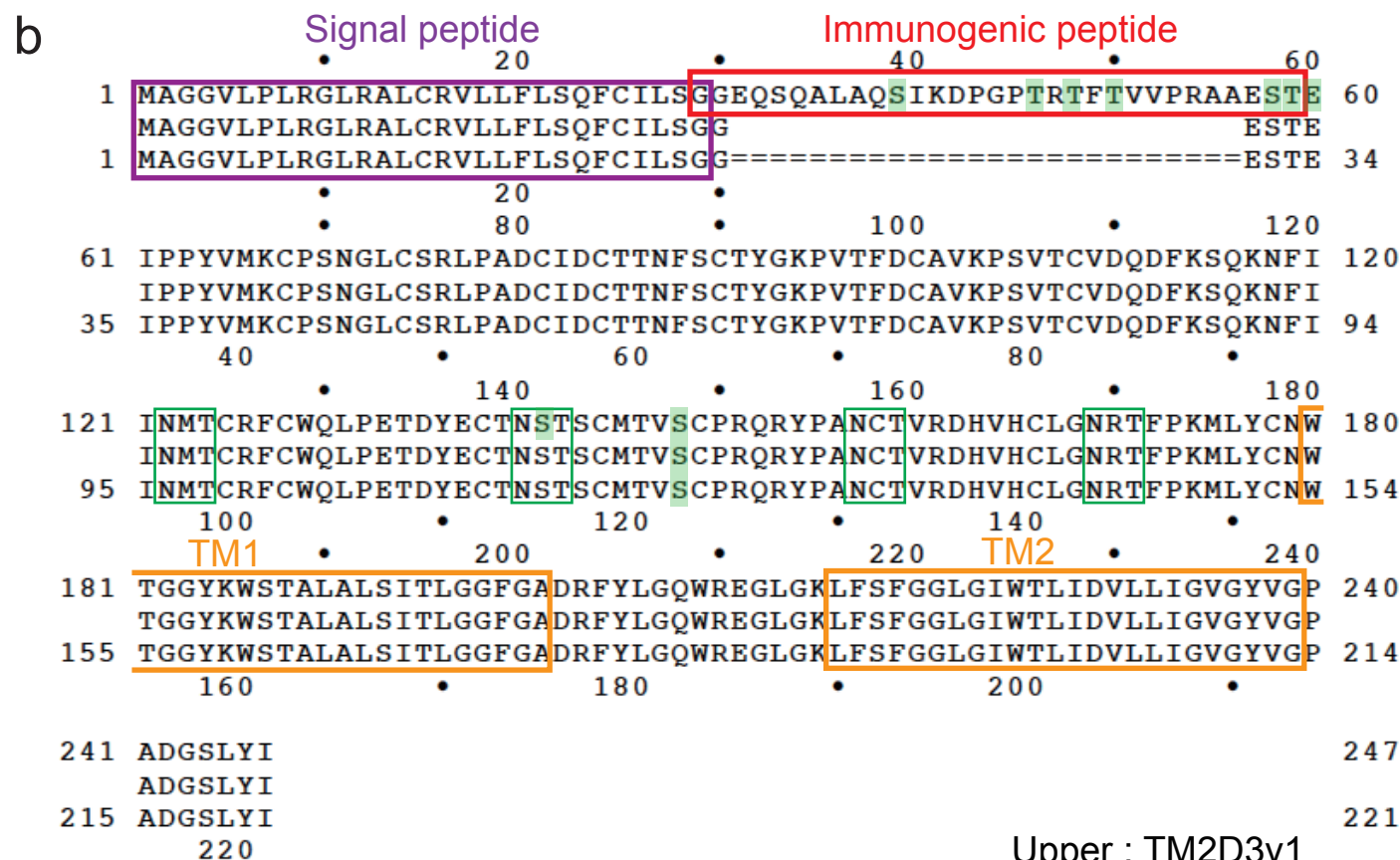

Upper : TM2D3v1

Lower : TM2D3v2

Supplement: Supplementary file 1 — Supplementary Information 1. [file 41598_2023_46866_MOESM1_ESM.pdf]

Supplementary Figure S2

Figure 1a

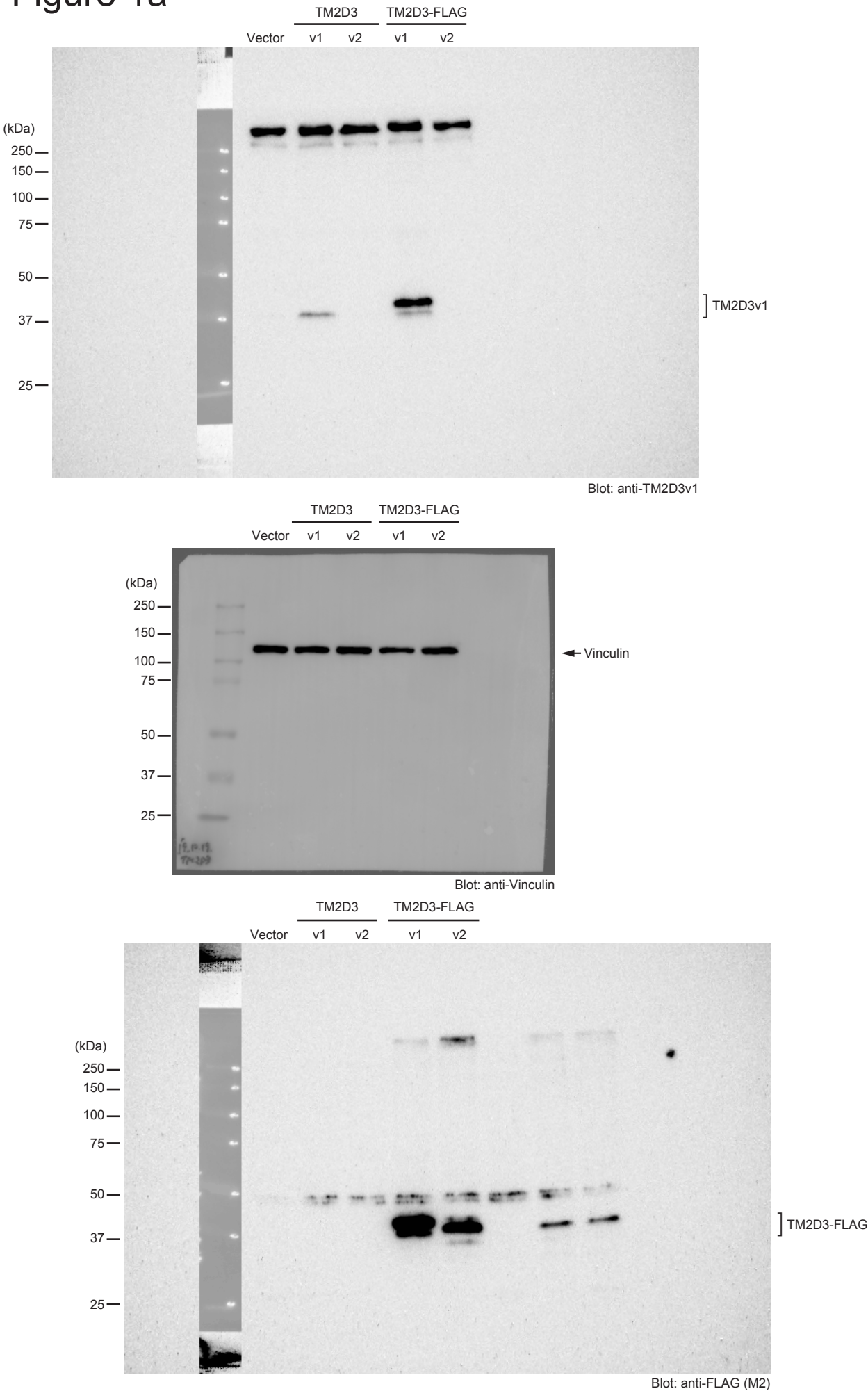

Supplement: Supplementary file 2 — Supplementary Information 2. [file 41598_2023_46866_MOESM2_ESM.pdf]

Supplementary Figure S2 (continued)

Figure 1b

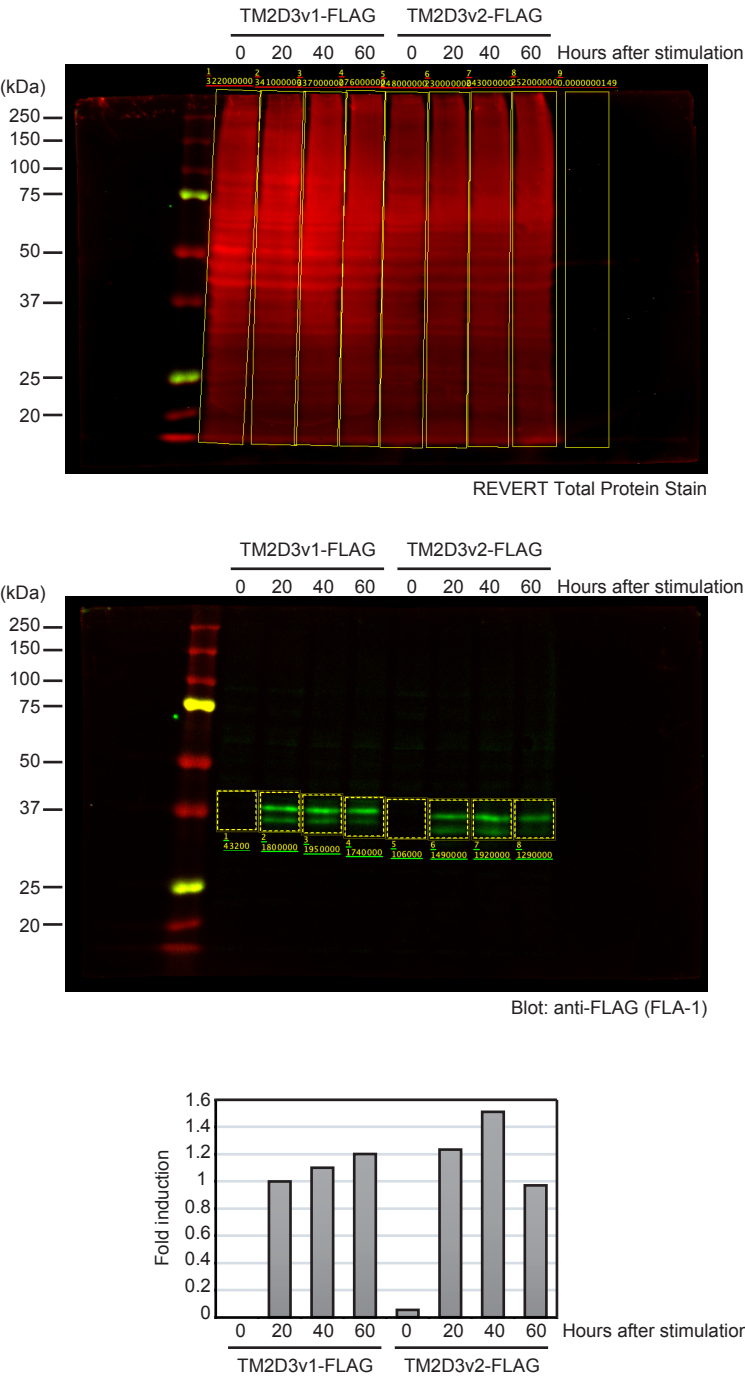

Figure 1c

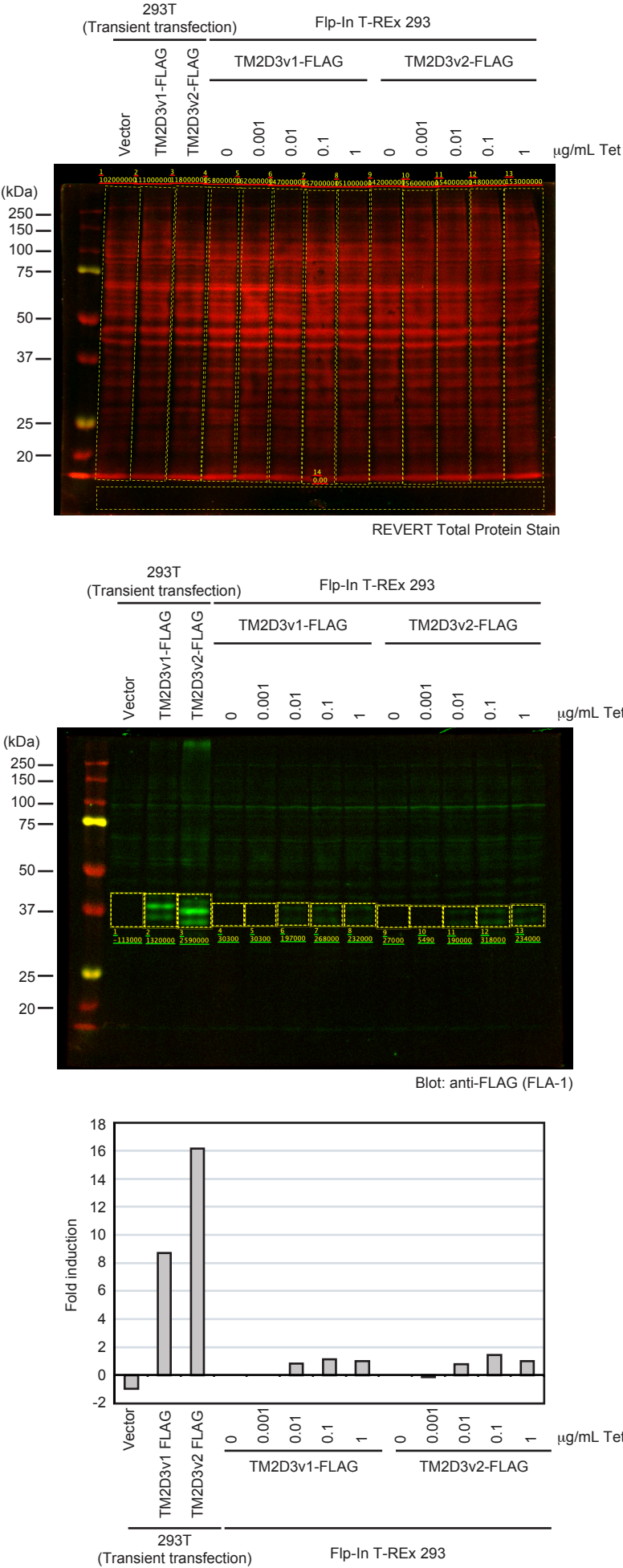

Supplement: Supplementary file 3 — Supplementary Information 3. [file 41598_2023_46866_MOESM3_ESM.pdf]

# Supplementary Figure S3

## Experiment #1

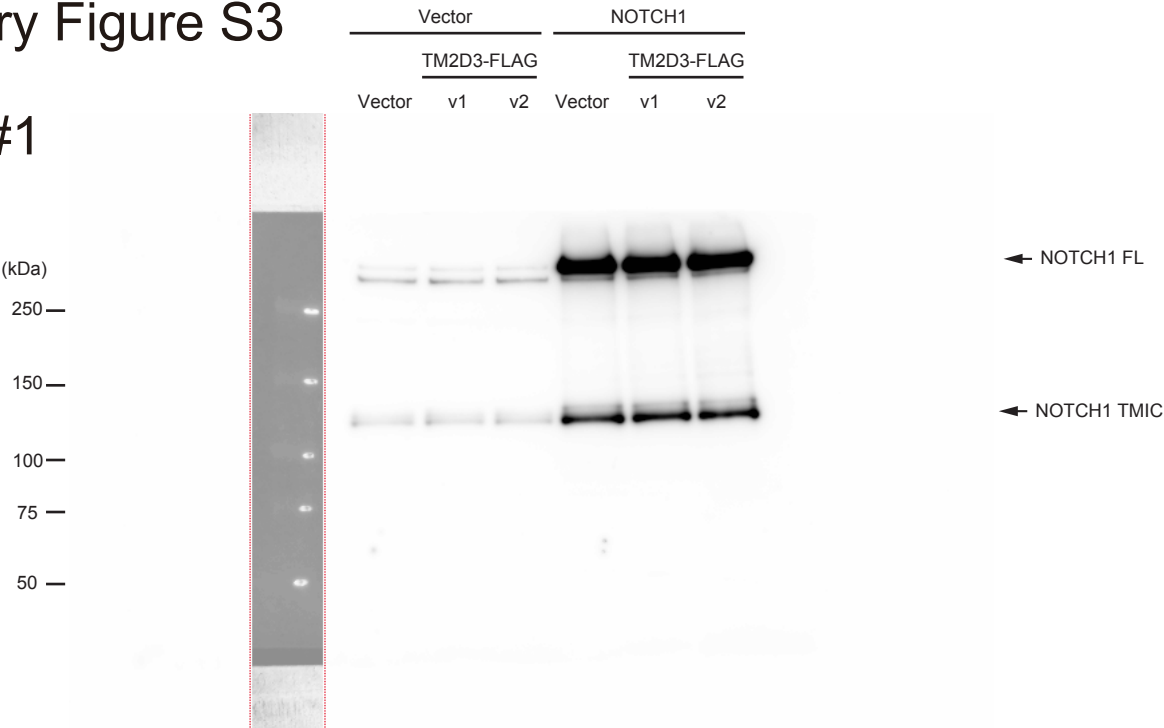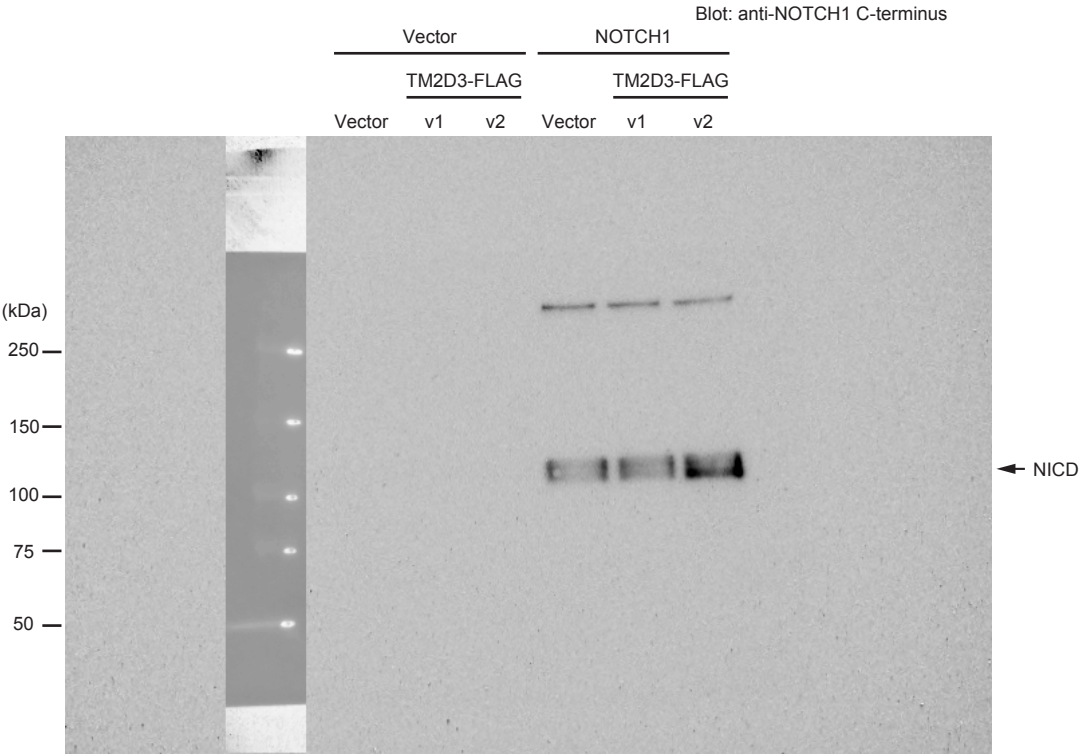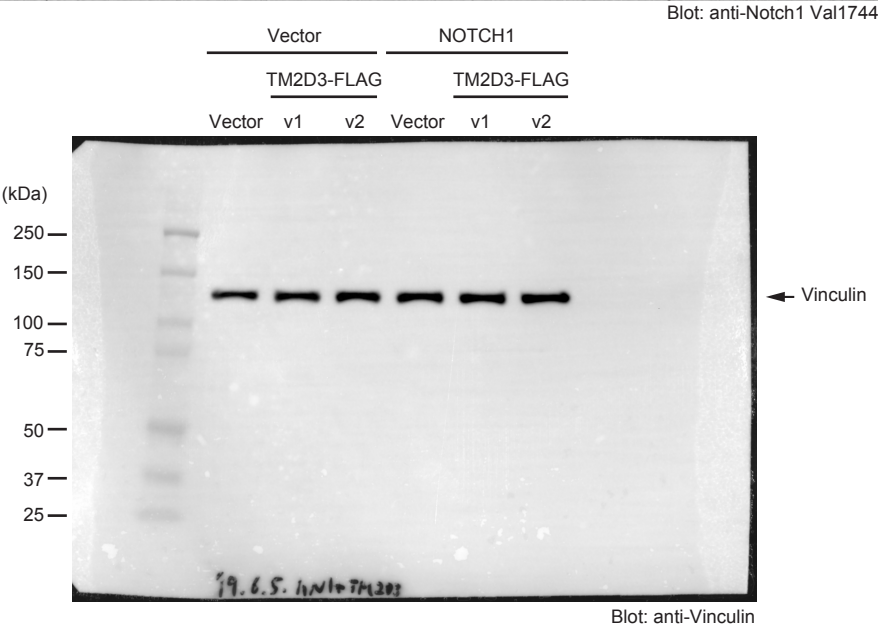

Blot: anti-Vinculin

Supplement: Supplementary file 4 — Supplementary Information 4. [file 41598_2023_46866_MOESM4_ESM.pdf]

# Supplementary Figure S3 (continued 1)

## Experiment #2

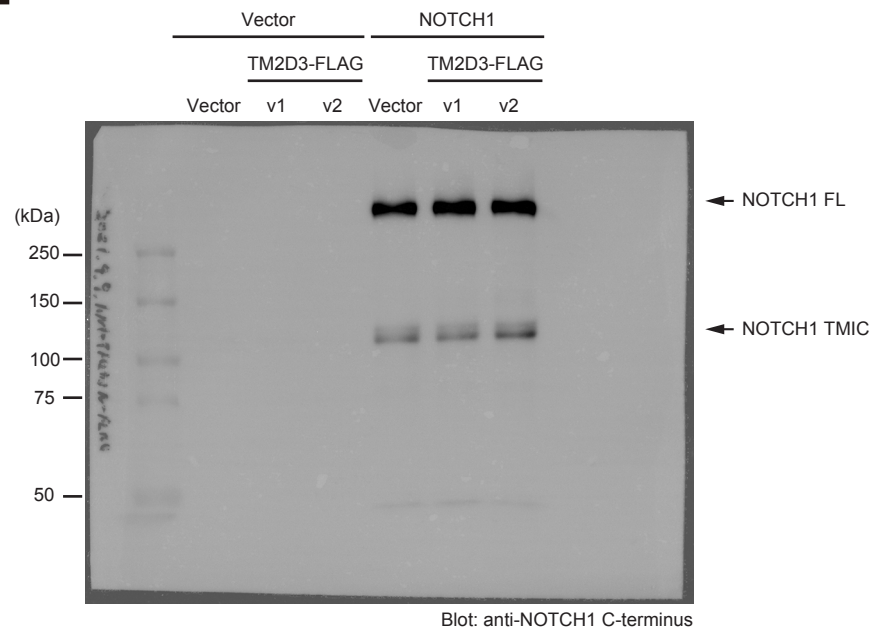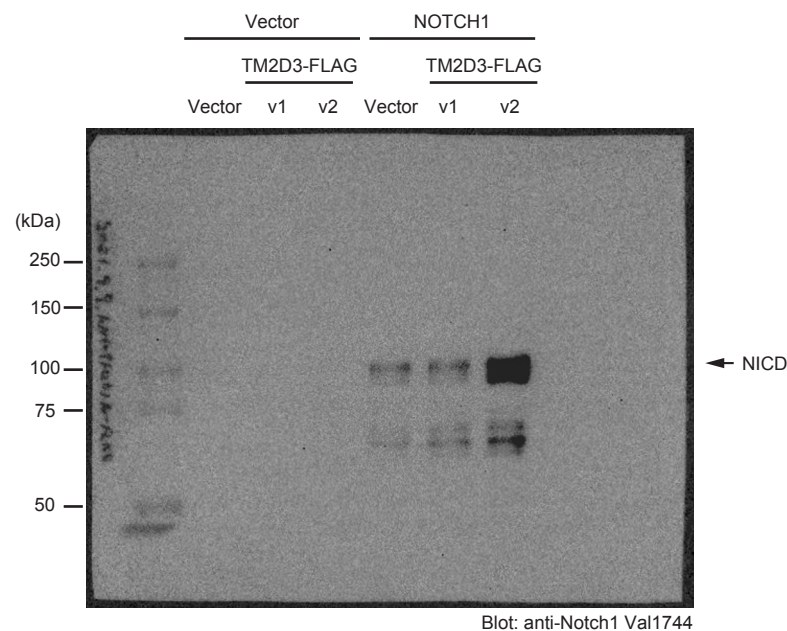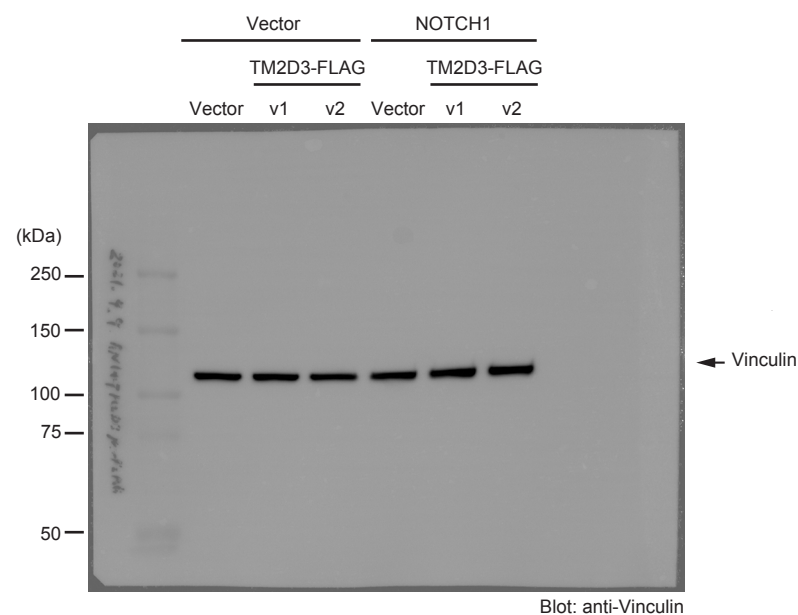

Supplement: Supplementary file 5 — Supplementary Information 5. [file 41598_2023_46866_MOESM5_ESM.pdf]

# Supplementary Figure S3 (continued 2)

## Experiment #3

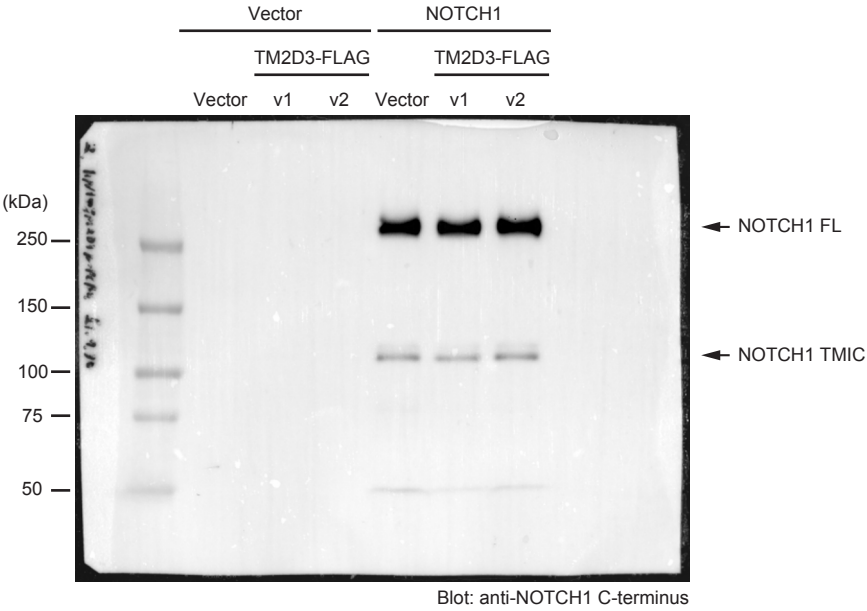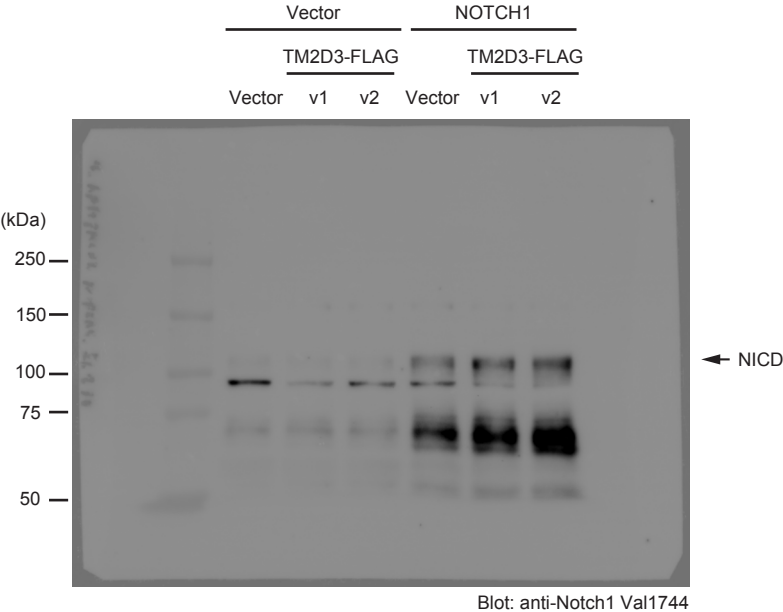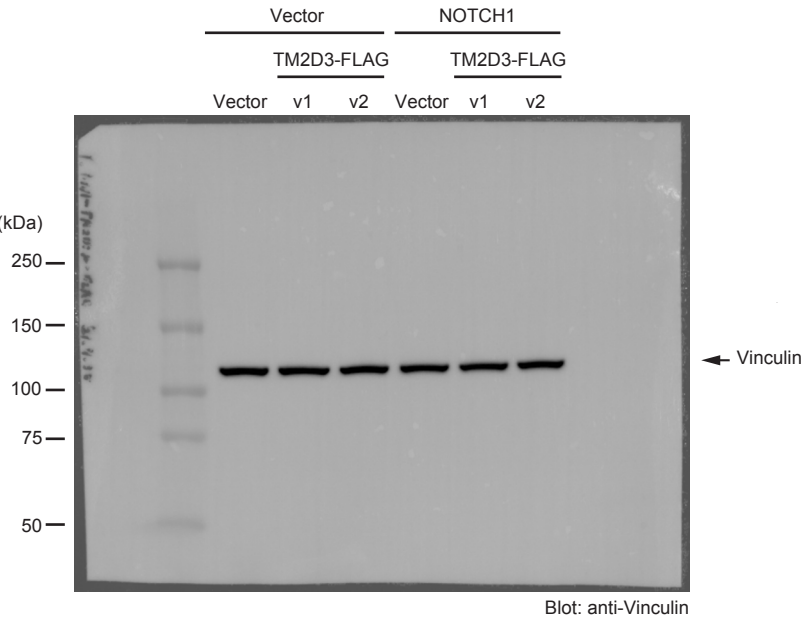

Supplement: Supplementary file 6 — Supplementary Information 6. [file 41598_2023_46866_MOESM6_ESM.pdf]

# Supplementary Figure S4

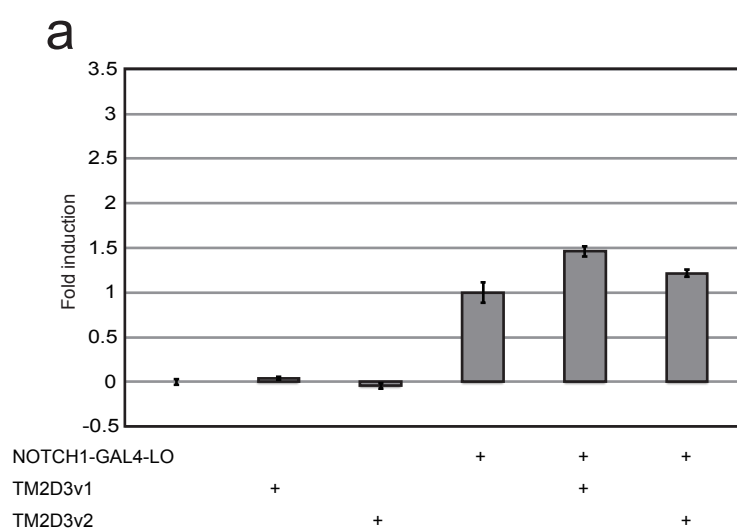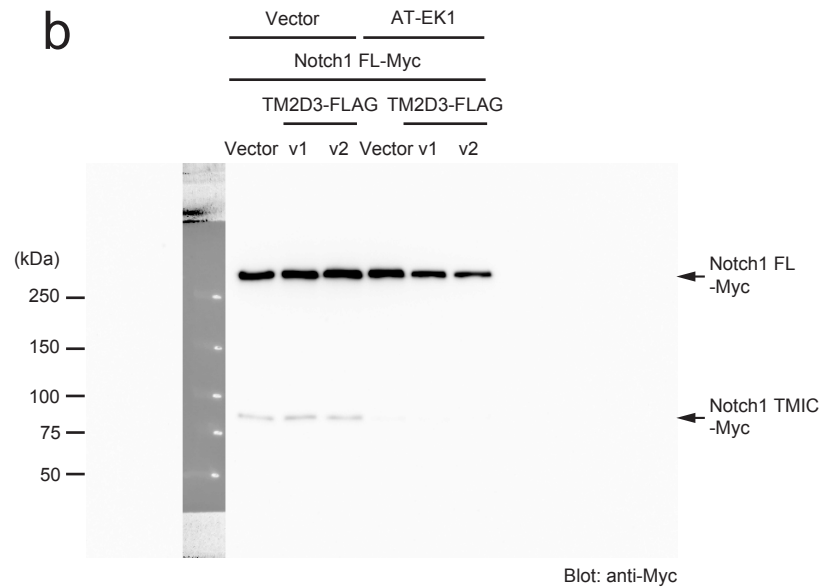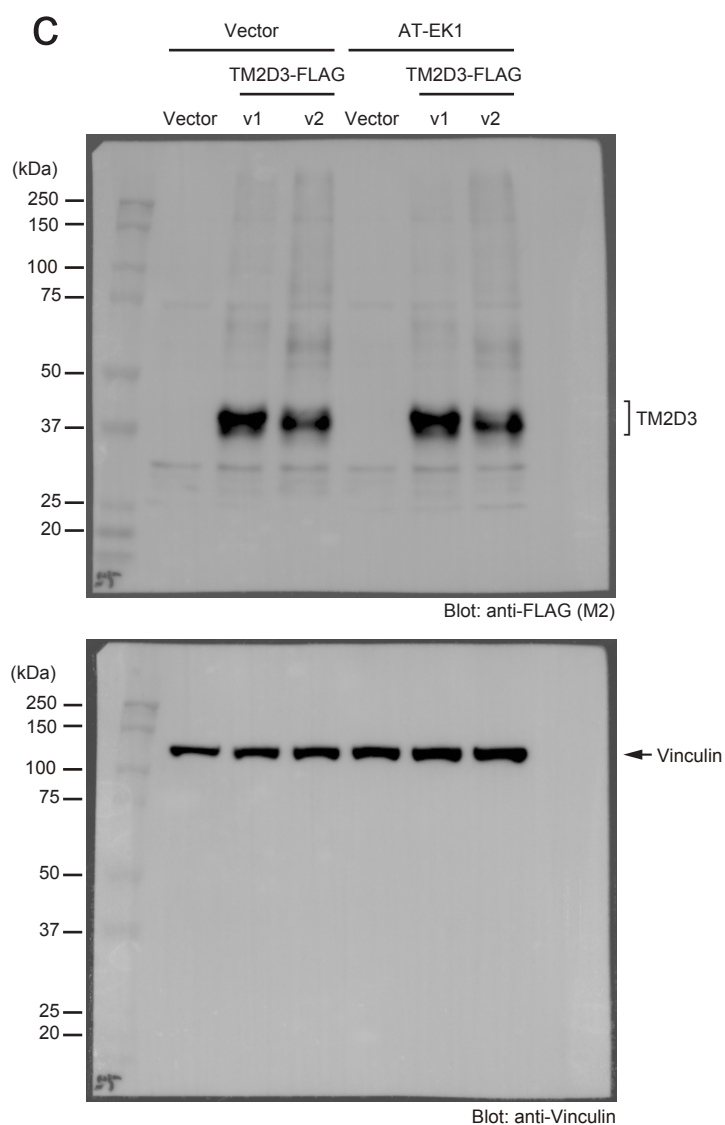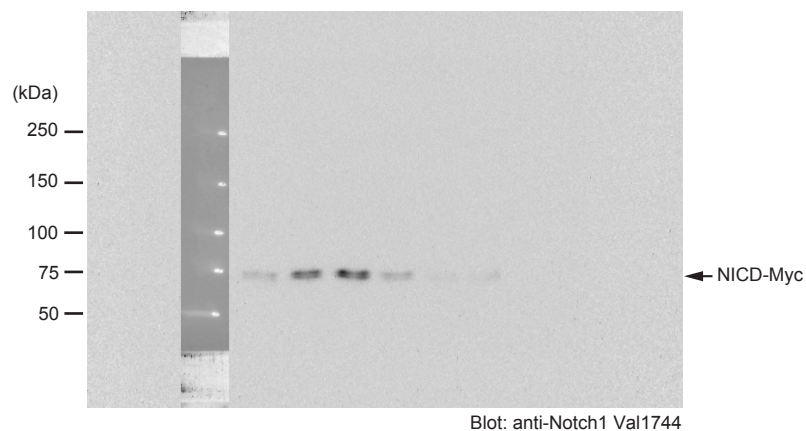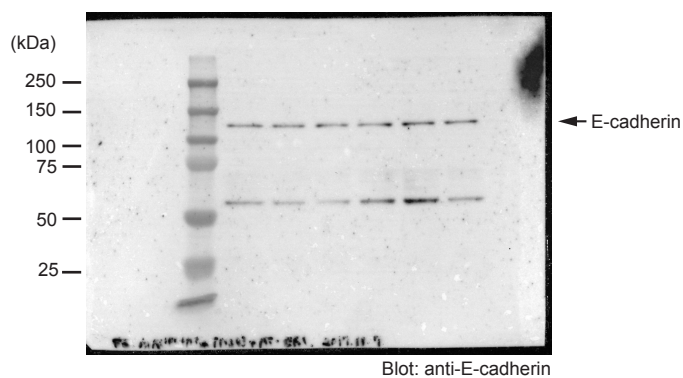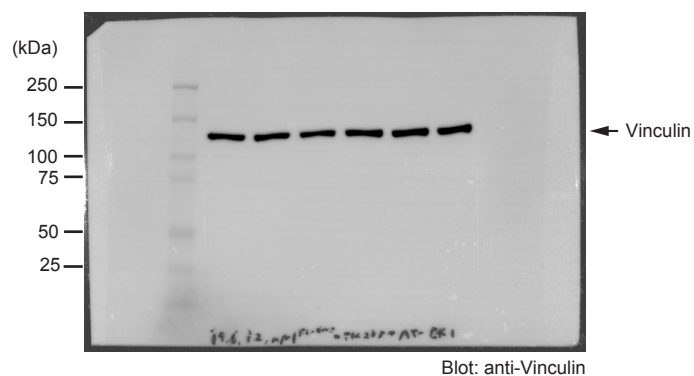

Supplement: Supplementary file 7 — Supplementary Information 7. [file 41598_2023_46866_MOESM7_ESM.pdf]

Supplementary Figure S5

a

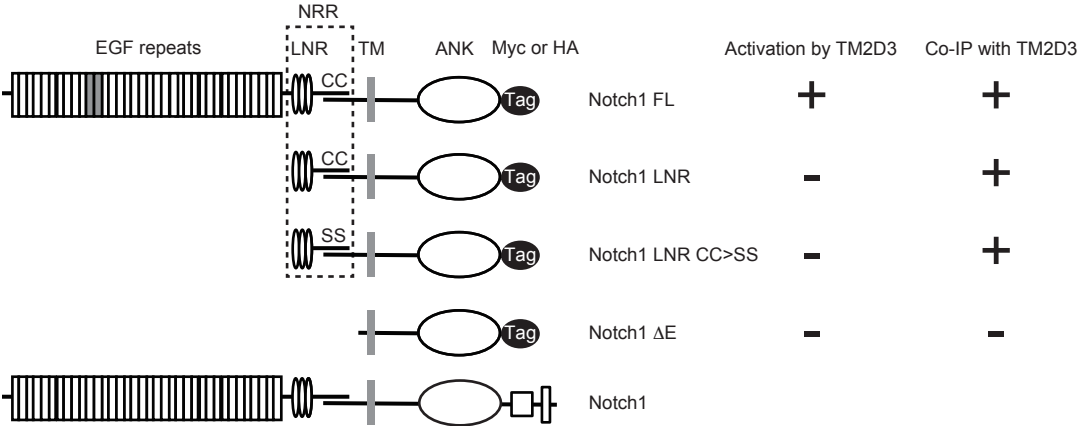

b

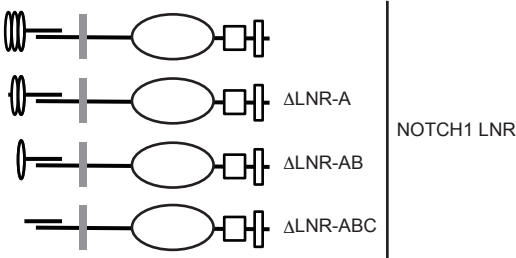

Supplement: Supplementary file 8 — Supplementary Information 8. [file 41598_2023_46866_MOESM8_ESM.pdf]

# Supplementary Figure S6

a

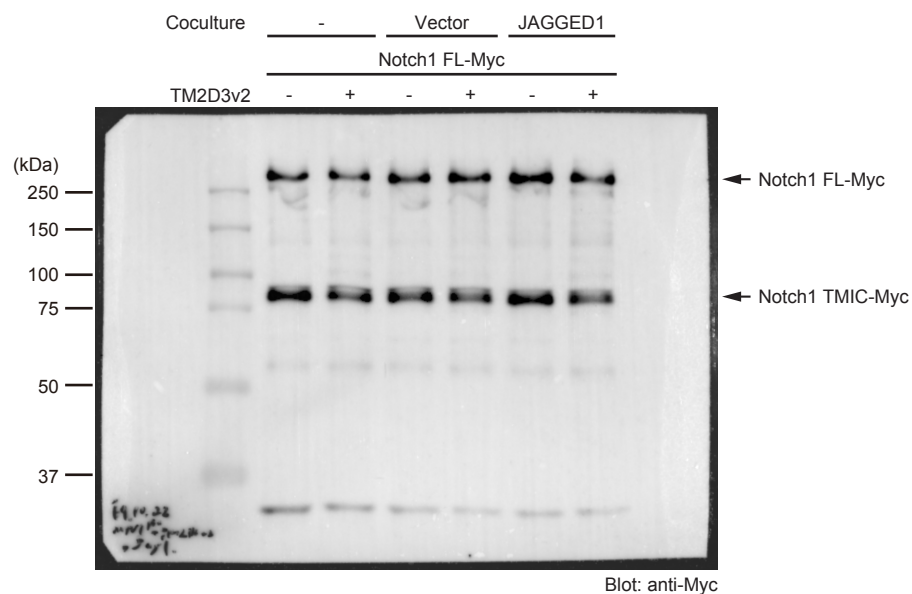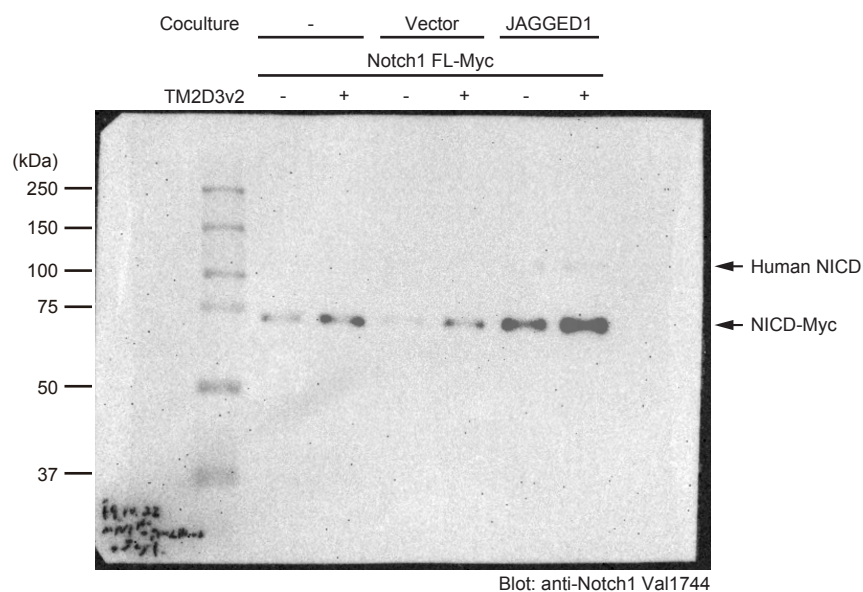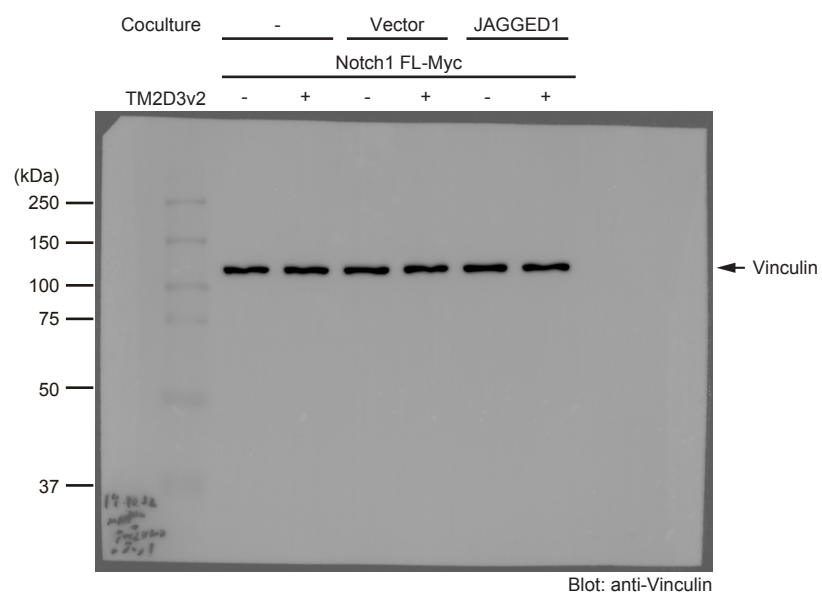

Supplement: Supplementary file 9 — Supplementary Information 9. [file 41598_2023_46866_MOESM9_ESM.pdf]

# Supplementary Figure S6 (continued)

b

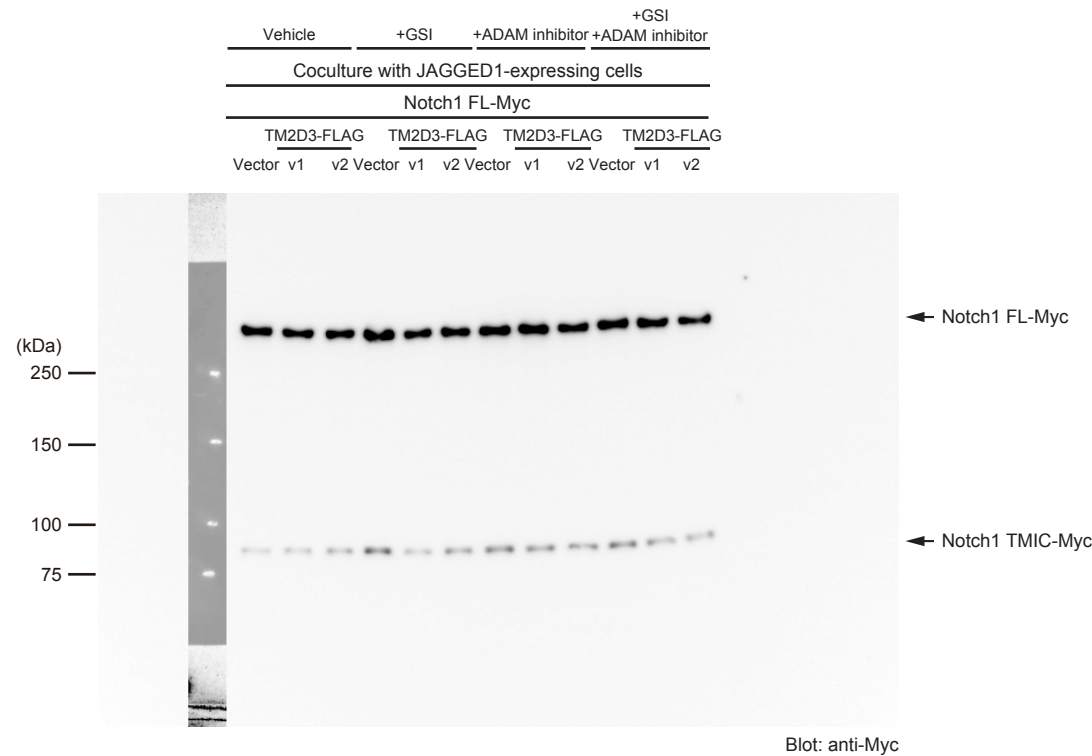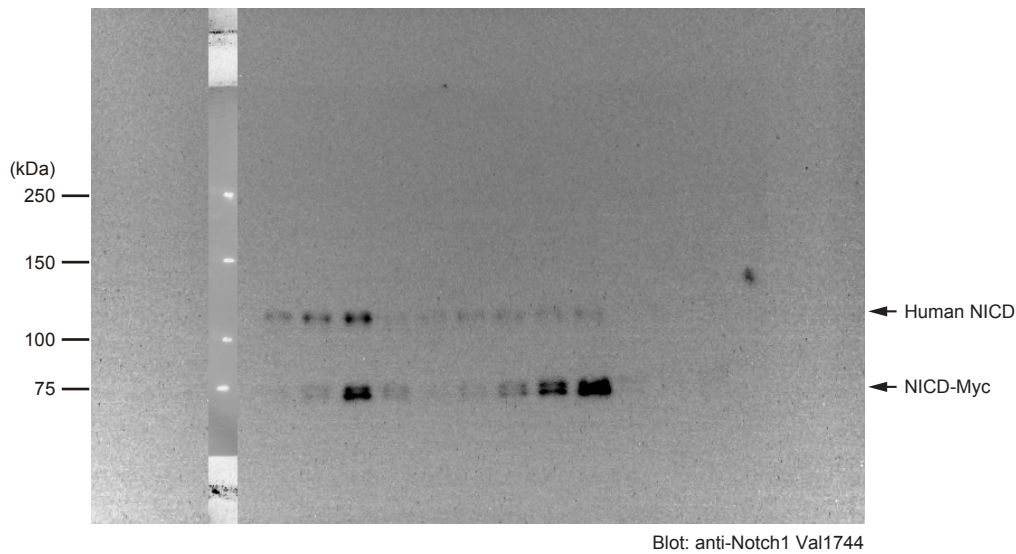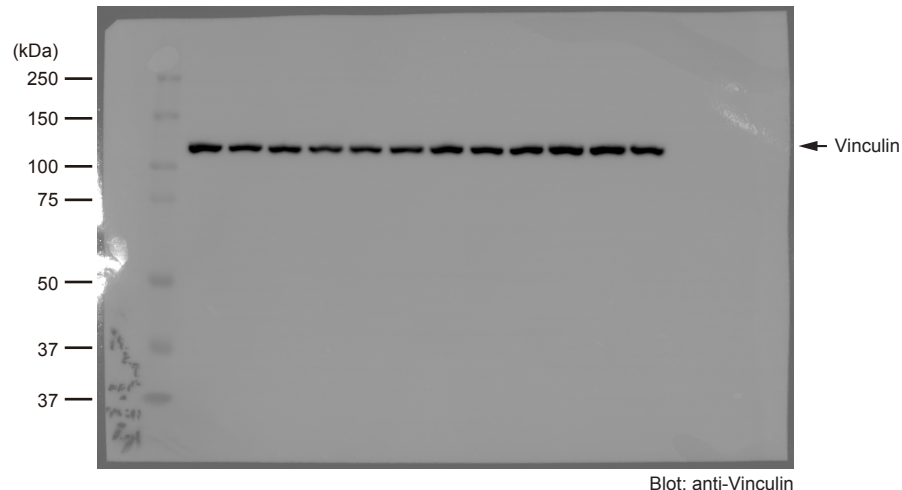

Supplement: Supplementary file 10 — Supplementary Information 10. [file 41598_2023_46866_MOESM10_ESM.pdf]

Supplementary Figure S7

Figure 3a

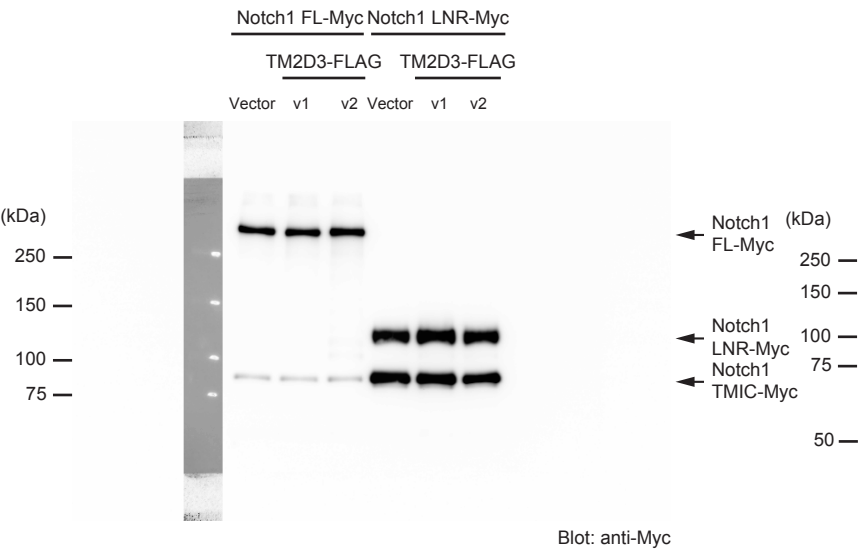

Figure 3b

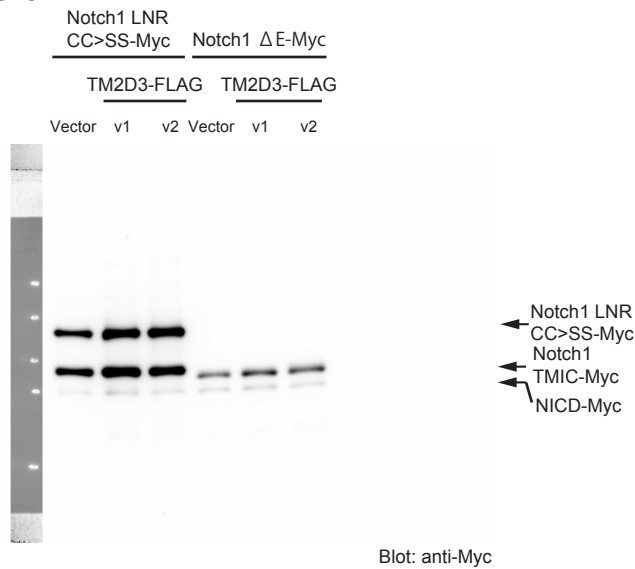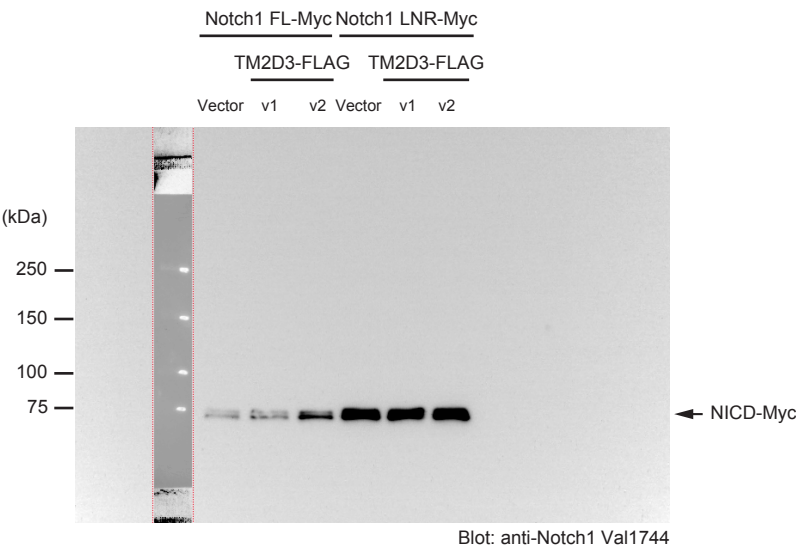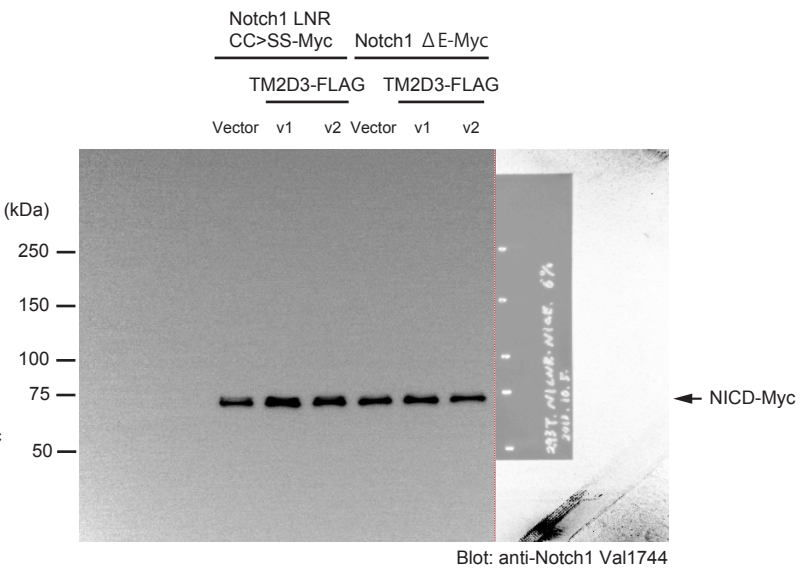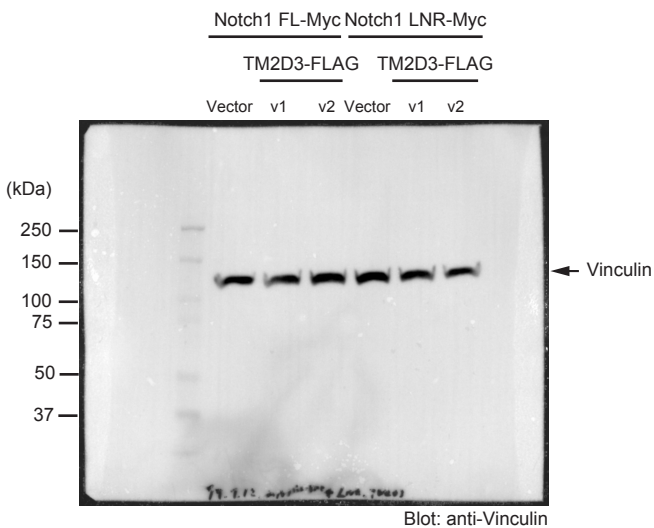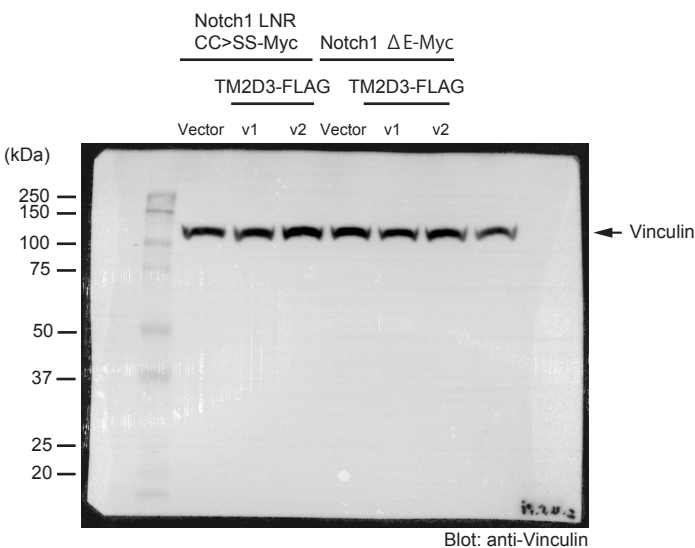

Supplement: Supplementary file 11 — Supplementary Information 11. [file 41598_2023_46866_MOESM11_ESM.pdf]

Supplementary Figure S7 (continued 1)

Figure 3c

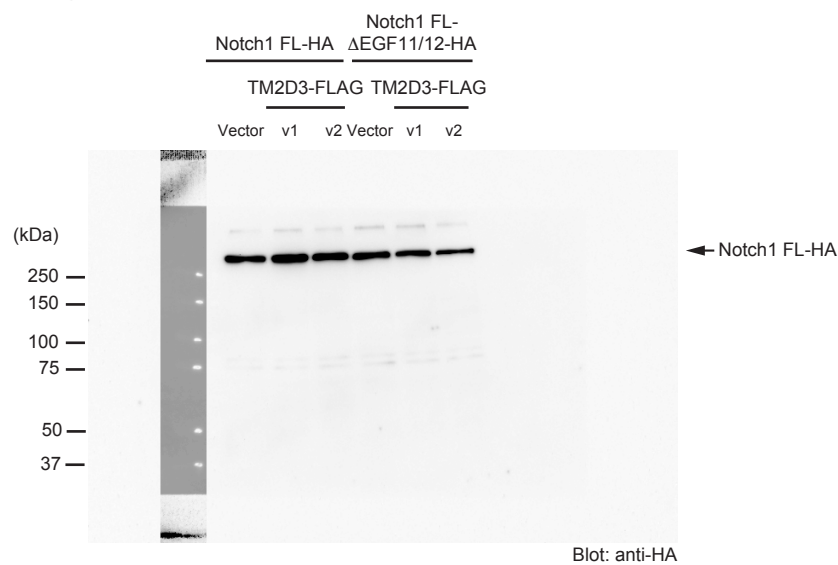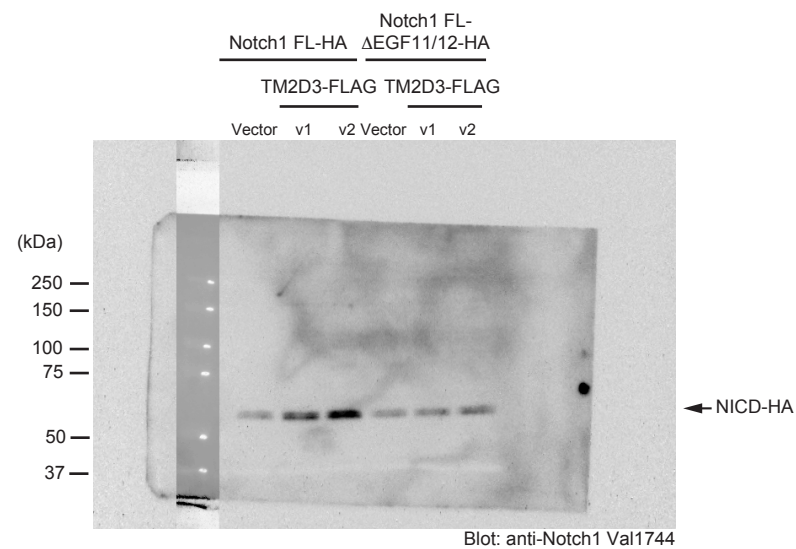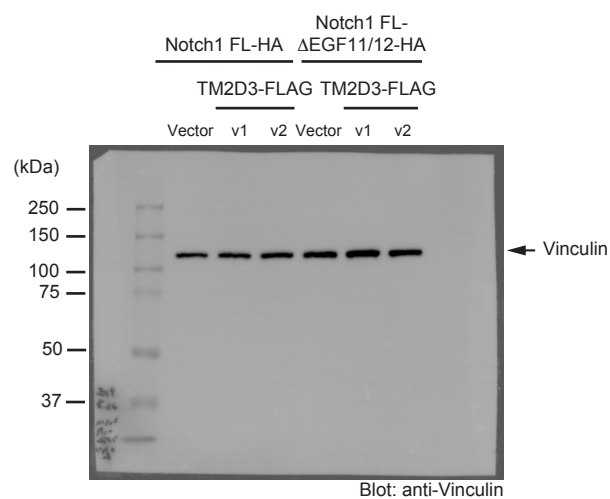

Supplement: Supplementary file 12 — Supplementary Information 12. [file 41598_2023_46866_MOESM12_ESM.pdf]

Supplementary Figure S7 (continued 2)

Figure 3d

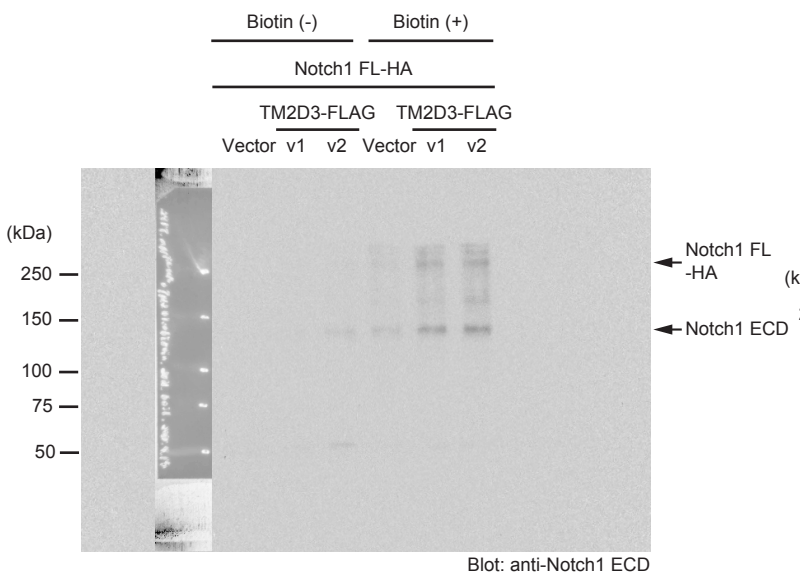

Figure 3e

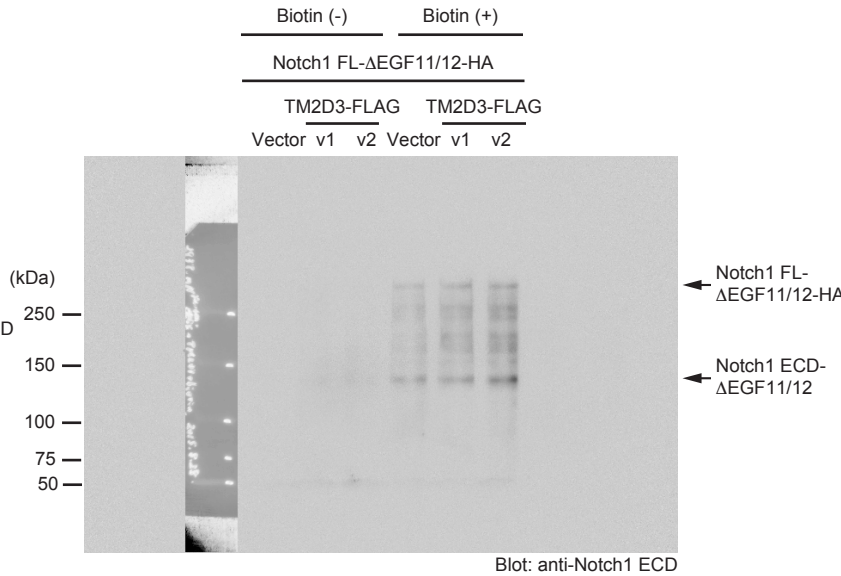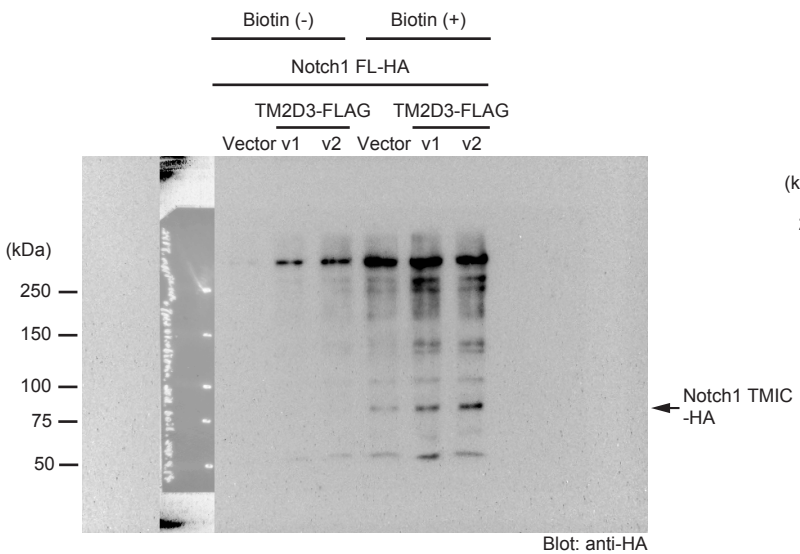

Figure 3f

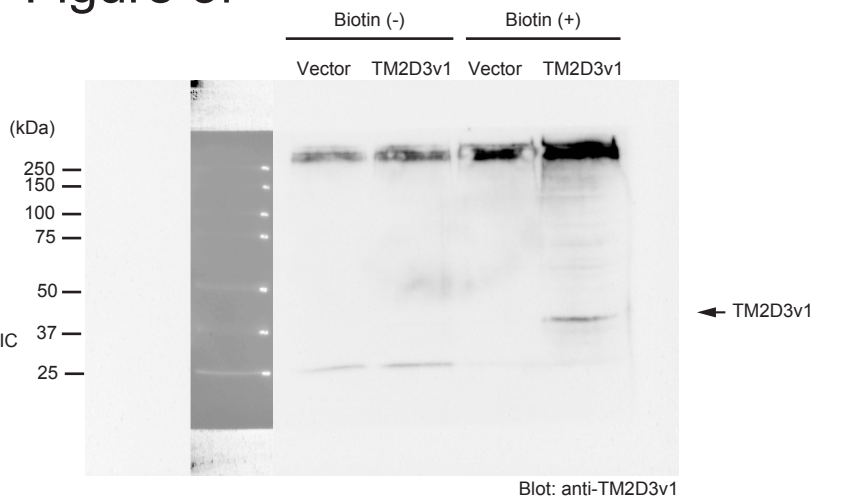

Supplement: Supplementary file 13 — Supplementary Information 13. [file 41598_2023_46866_MOESM13_ESM.pdf]

Supplementary Figure S8

Figure 4a

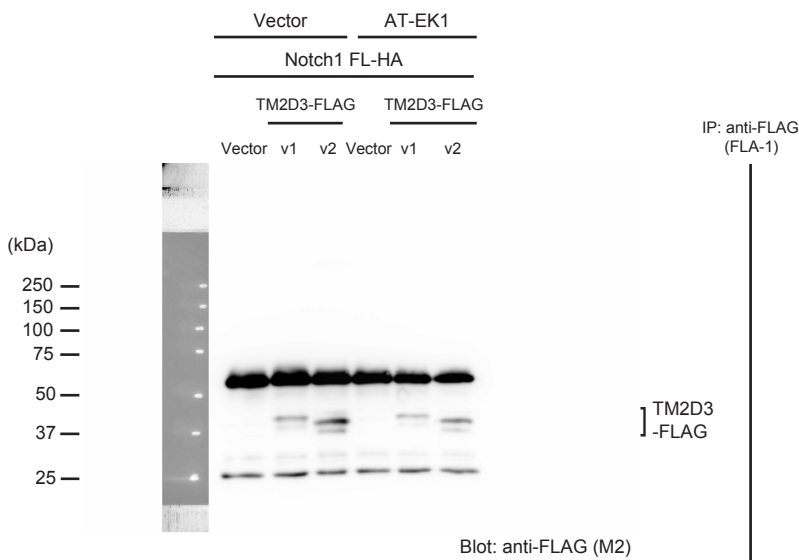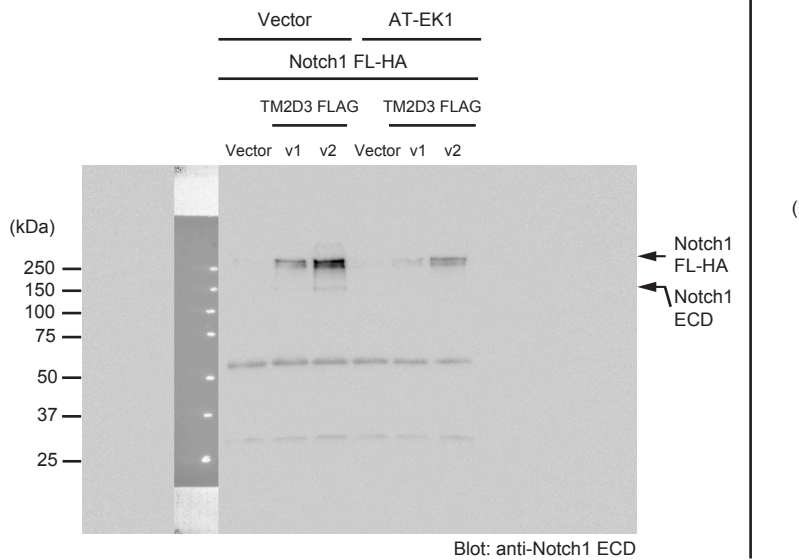

Figure 4b

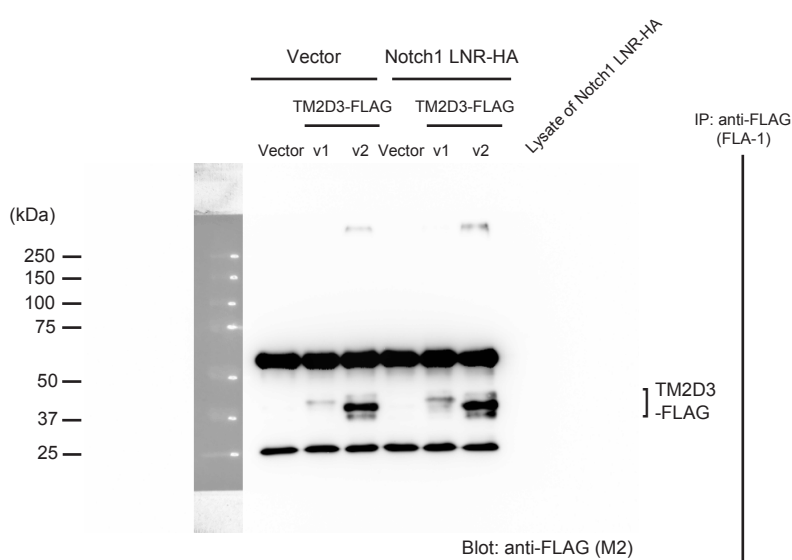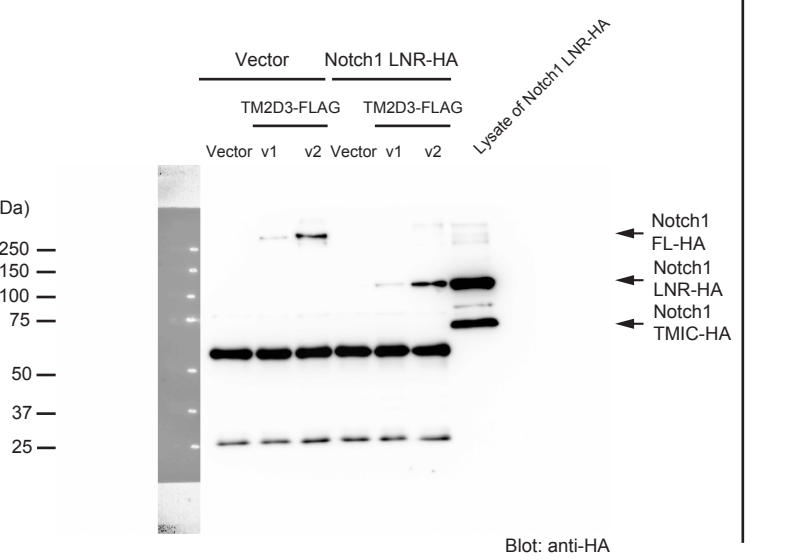

Supplement: Supplementary file 14 — Supplementary Information 14. [file 41598_2023_46866_MOESM14_ESM.pdf]

Supplementary Figure S8 (continued 1)

Figure 4c

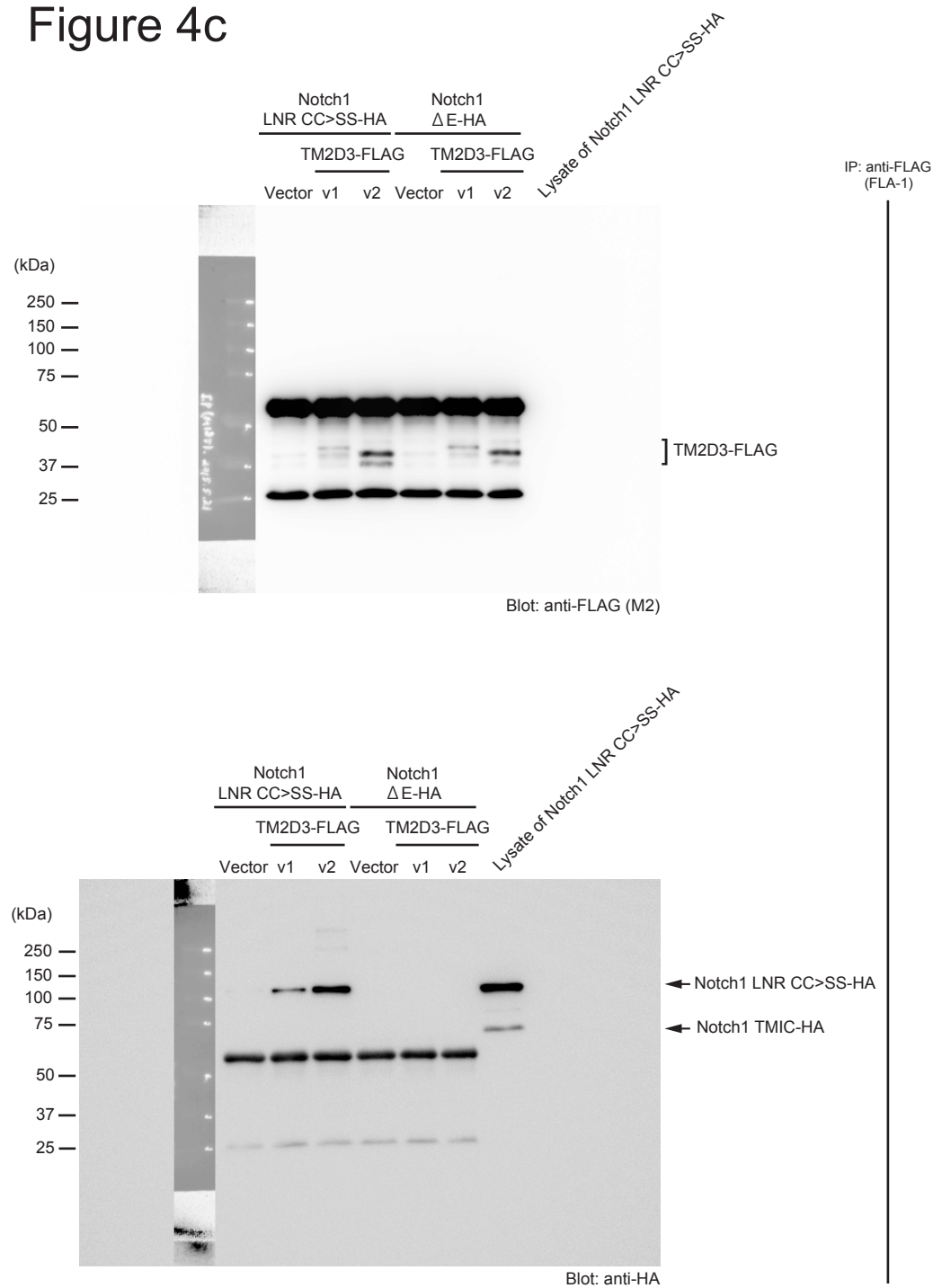

Supplement: Supplementary file 15 — Supplementary Information 15. [file 41598_2023_46866_MOESM15_ESM.pdf]

Supplementary Figure S8 (continued 2)

Figure 4d

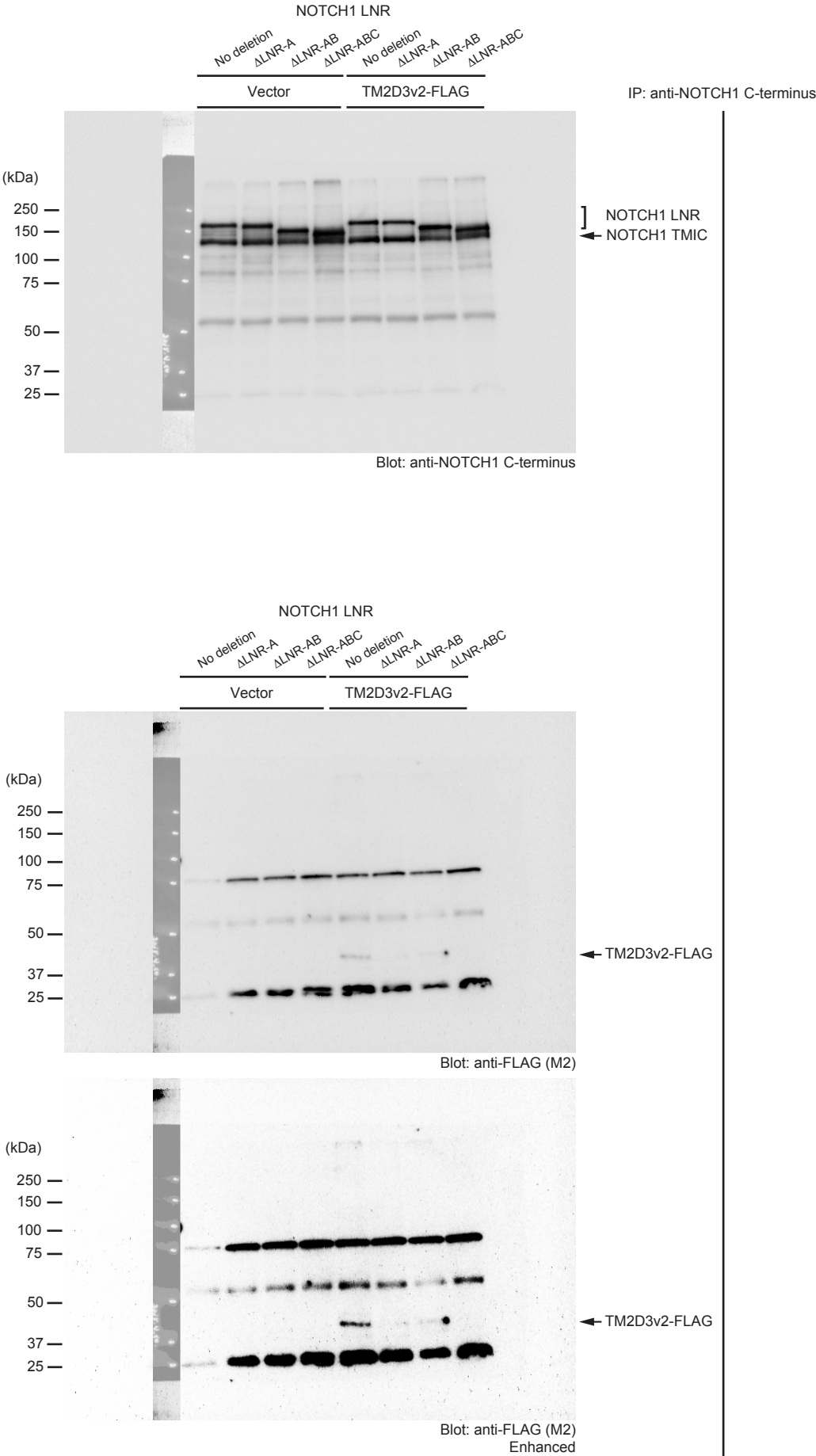

Supplement: Supplementary file 16 — Supplementary Information 16. [file 41598_2023_46866_MOESM16_ESM.pdf]

Supplementary Figure S8 (continued 3)

Figure 4e

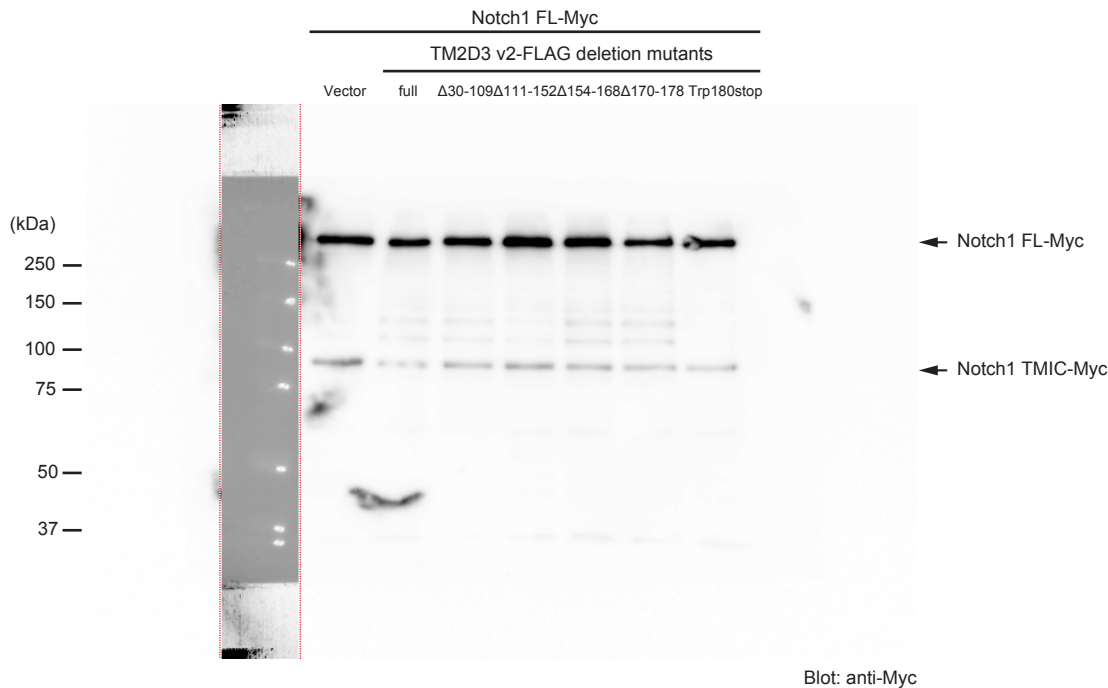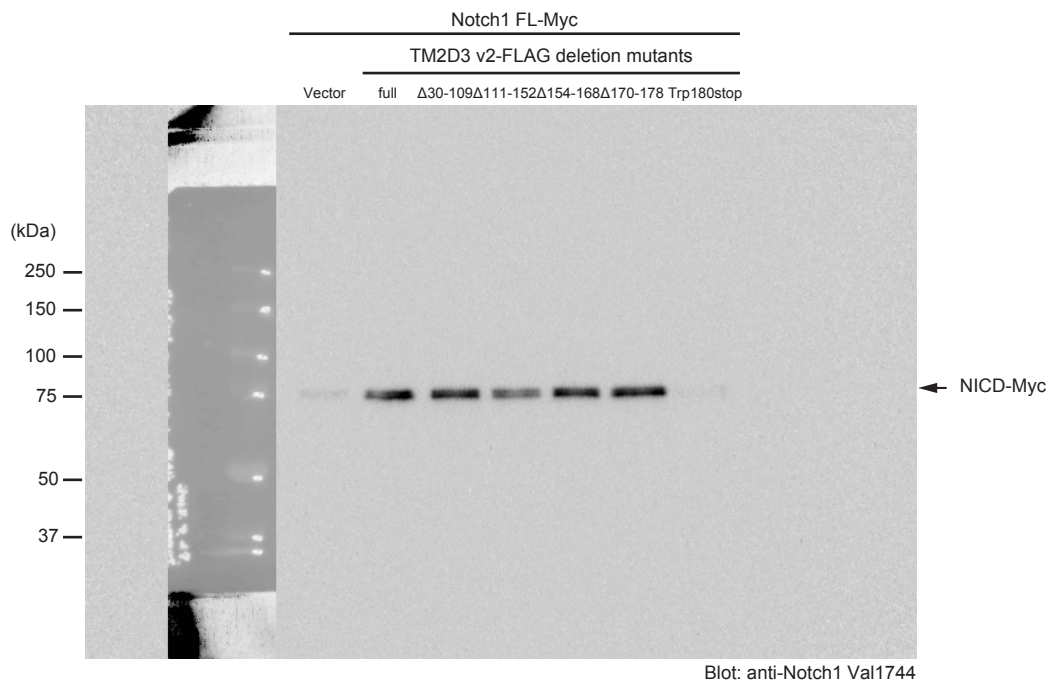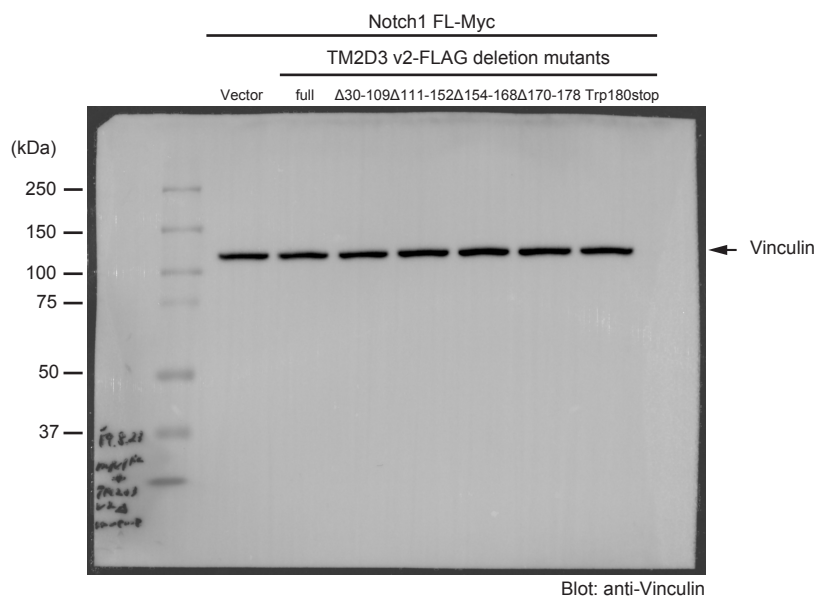

Supplement: Supplementary file 17 — Supplementary Information 17. [file 41598_2023_46866_MOESM17_ESM.pdf]

Supplementary Figure S8 (continued 4)

Figure 4f

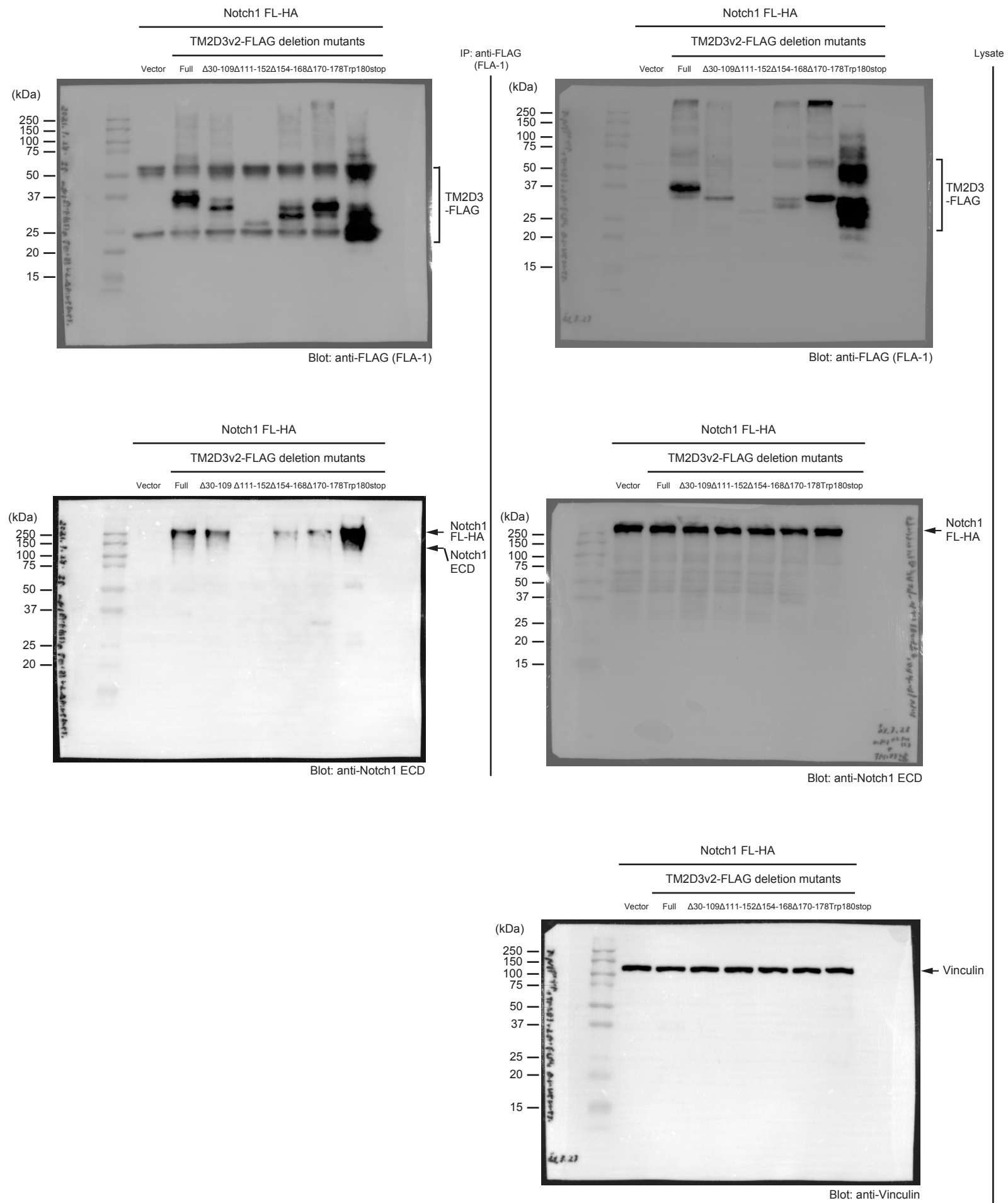

Supplement: Supplementary file 18 — Supplementary Information 18. [file 41598_2023_46866_MOESM18_ESM.pdf]

# Supplementary Figure S9

a

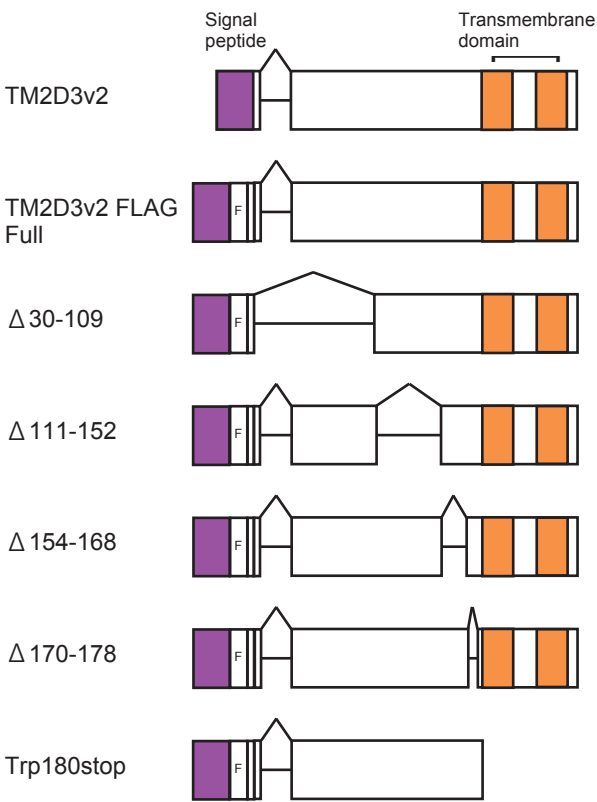

b

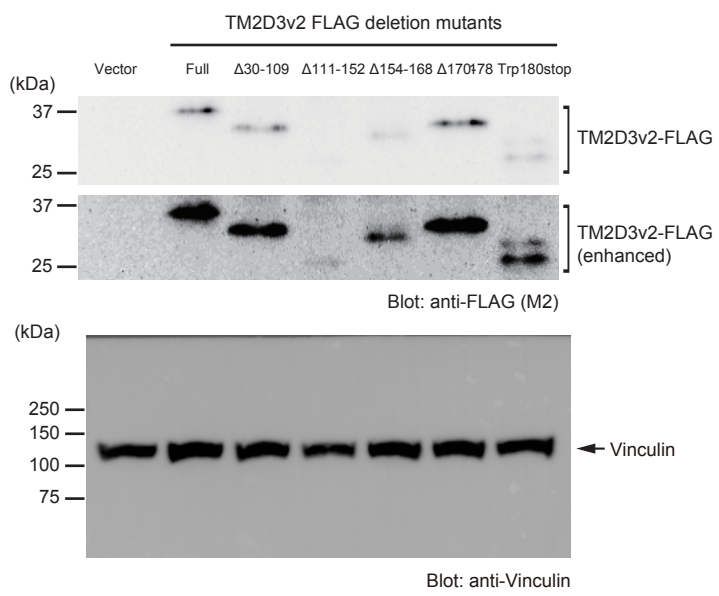

Supplement: Supplementary file 19 — Supplementary Information 19. [file 41598_2023_46866_MOESM19_ESM.pdf]

# Supplementary Figure S9 (continued)

C

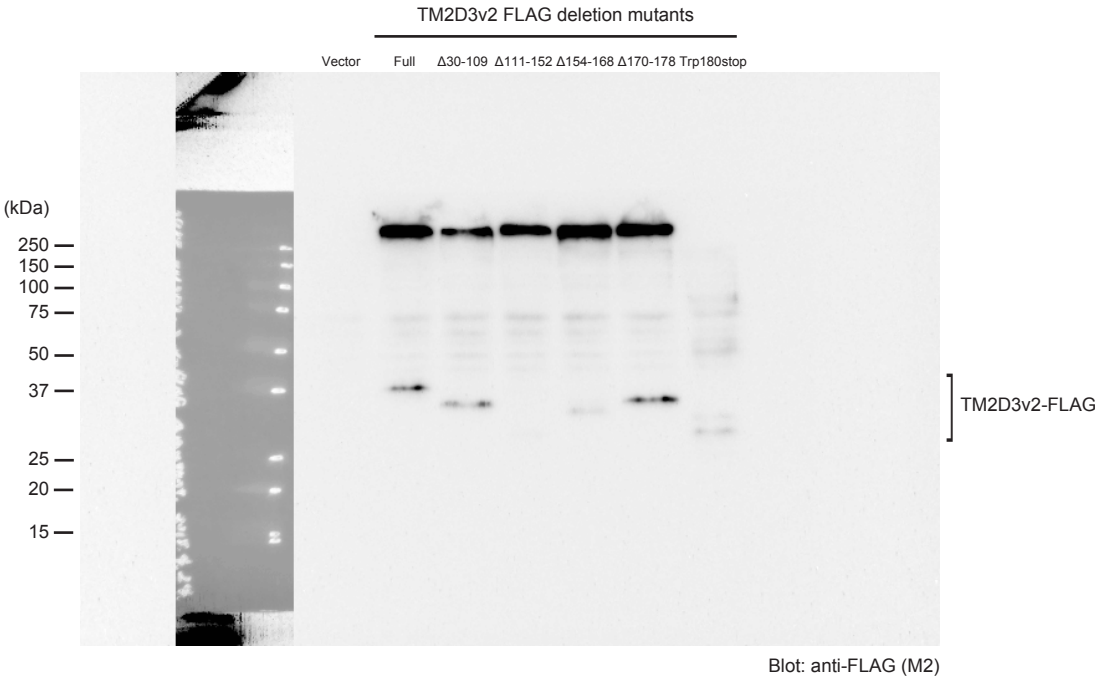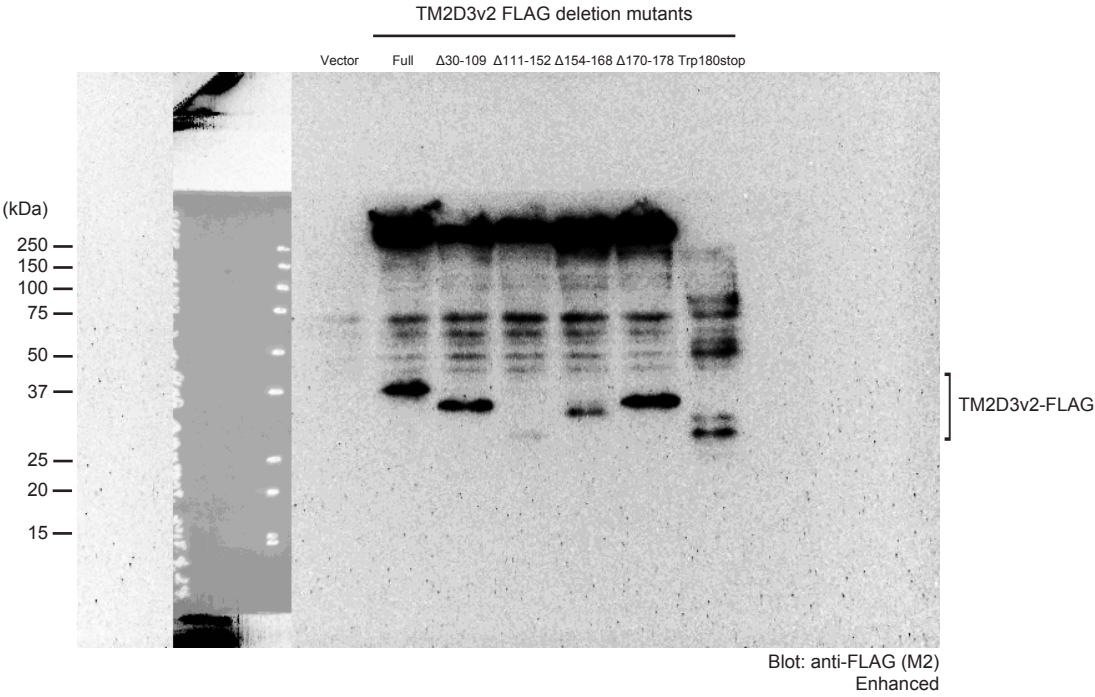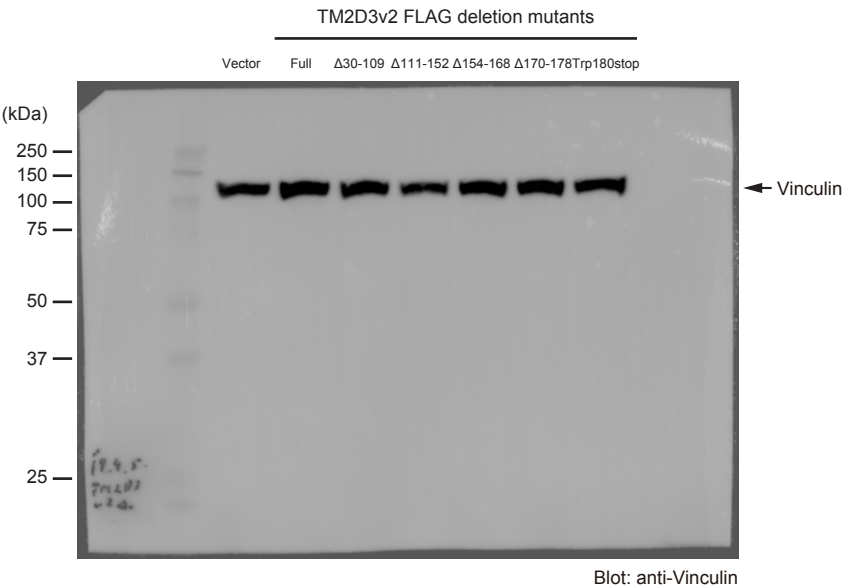

Supplement: Supplementary file 20 — Supplementary Information 20. [file 41598_2023_46866_MOESM20_ESM.pdf]

## Supplementary Figure S10

a

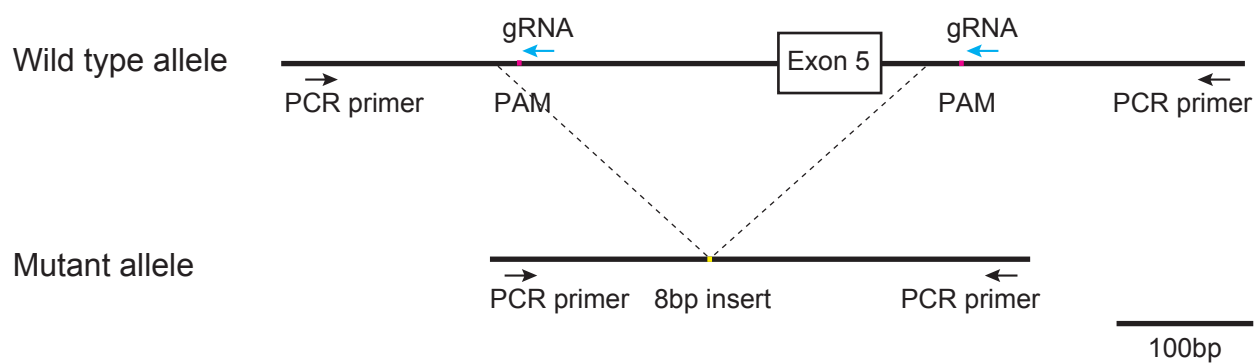

b

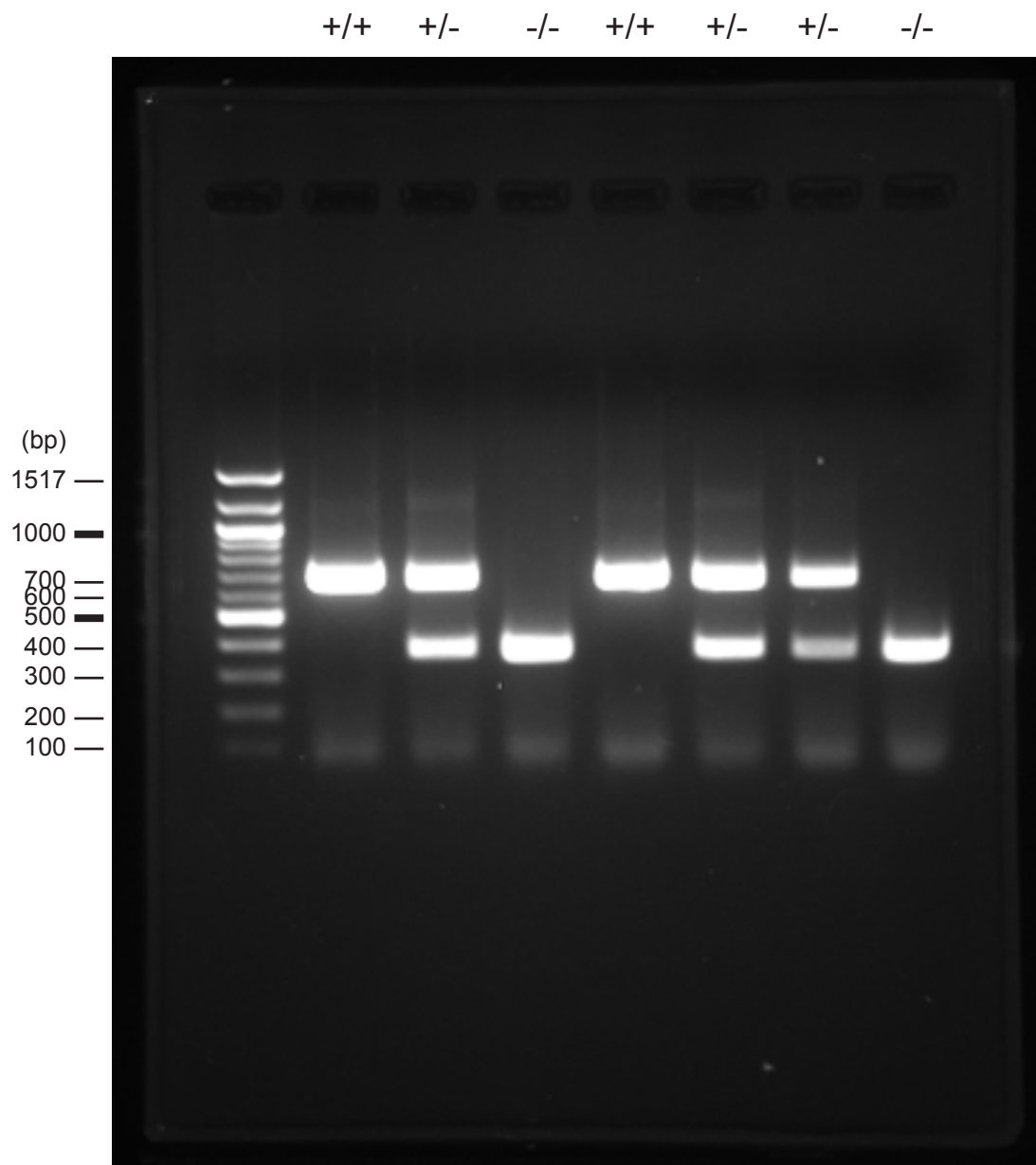

Supplement: Supplementary file 21 — Supplementary Information 21. [file 41598_2023_46866_MOESM21_ESM.pdf]

Supplementary Figure S11

Figure 5a

Whole cell lysates

Experiment #1

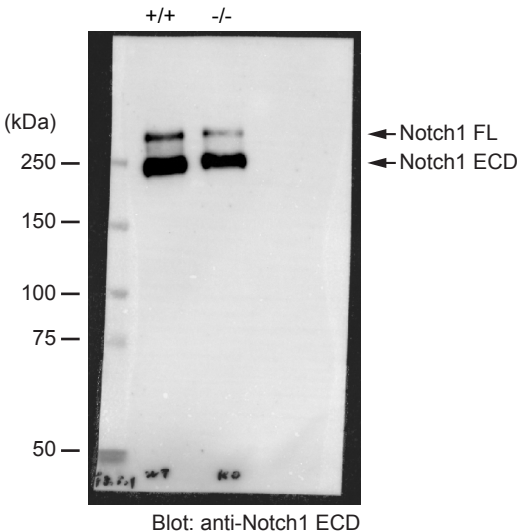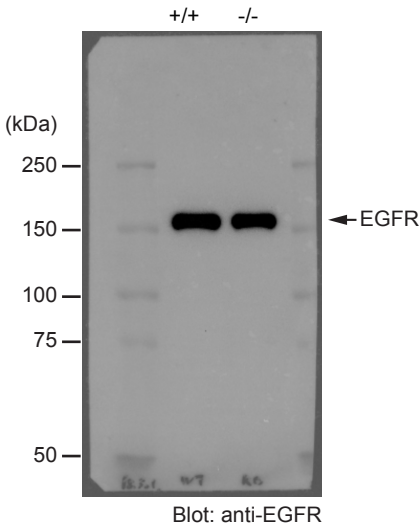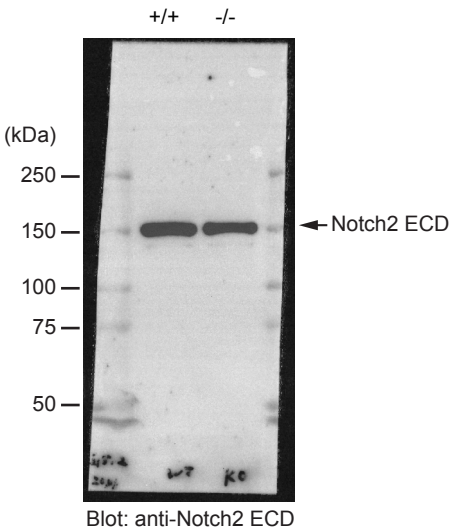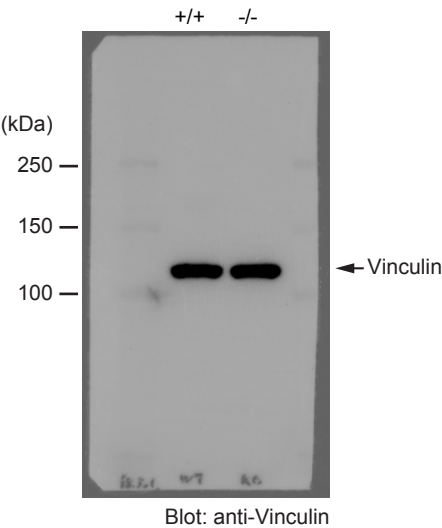

Supplement: Supplementary file 22 — Supplementary Information 22. [file 41598_2023_46866_MOESM22_ESM.pdf]

## Supplementary Figure S12

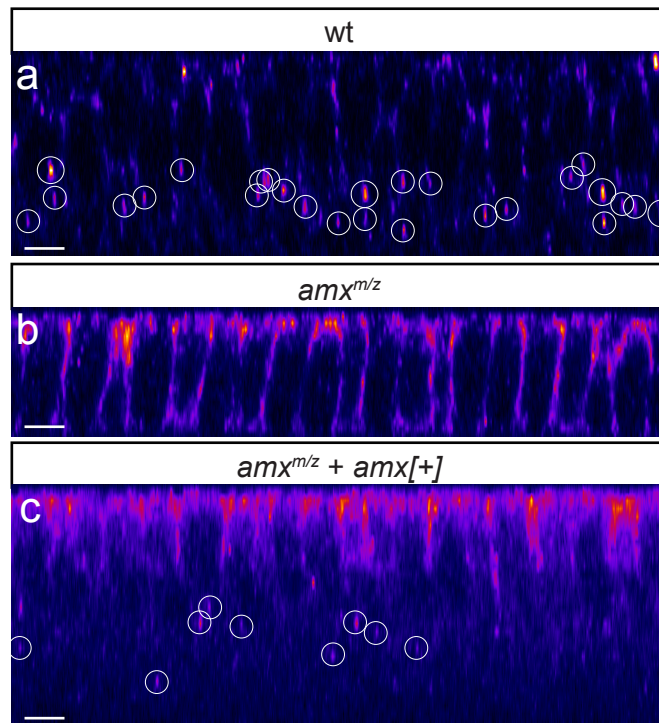

Stain: anti-NECD  
Bar = 5  $\mu$ m

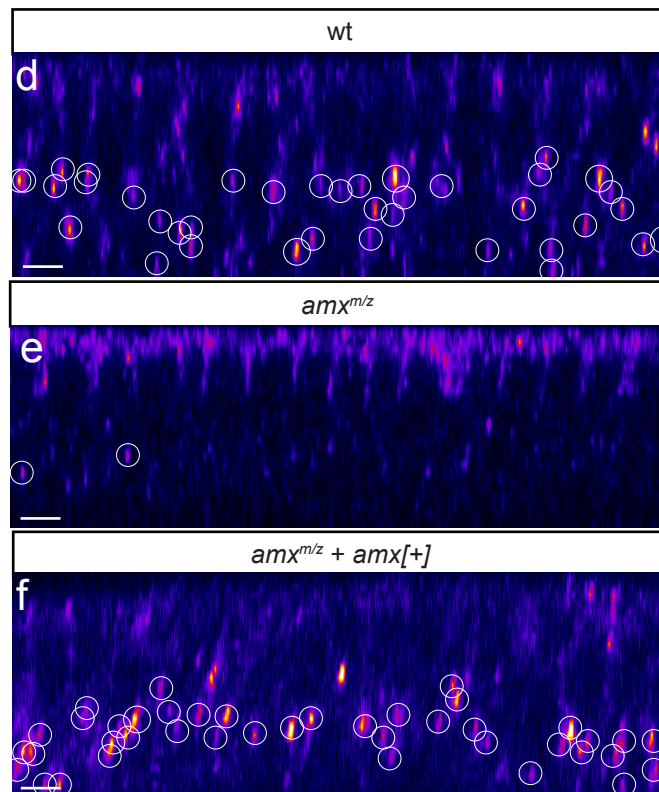

Stain: anti-DI  
Bar = 5  $\mu$ m

Supplement: Supplementary file 24 — Supplementary Information 24. [file 41598_2023_46866_MOESM24_ESM.pdf]

# Supplementary Figure S13

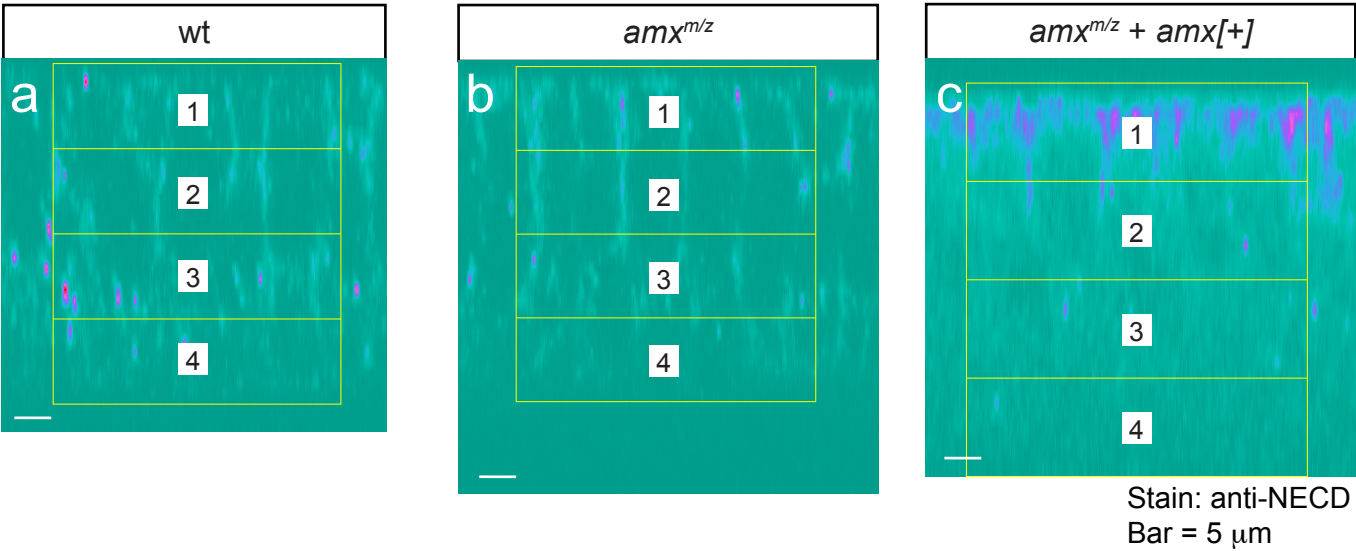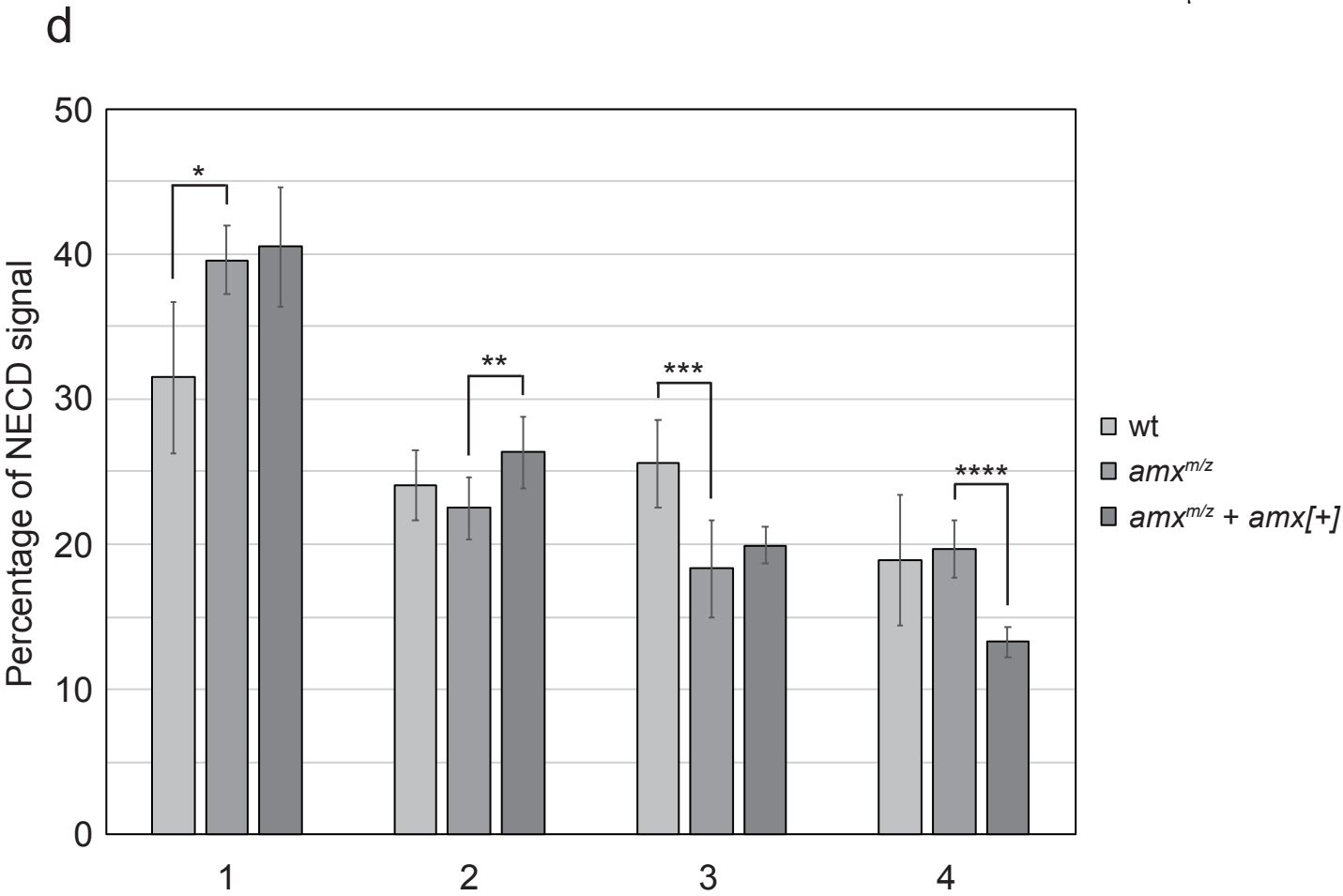

Supplement: Supplementary file 25 — Supplementary Information 25. [file 41598_2023_46866_MOESM25_ESM.pdf]

# Supplementary Figure S13 (continued 1)

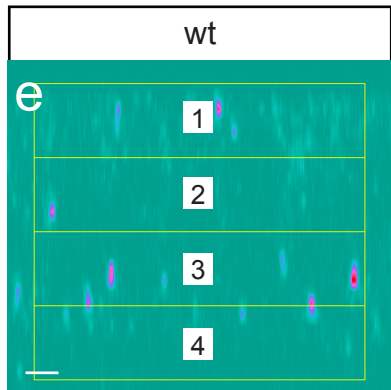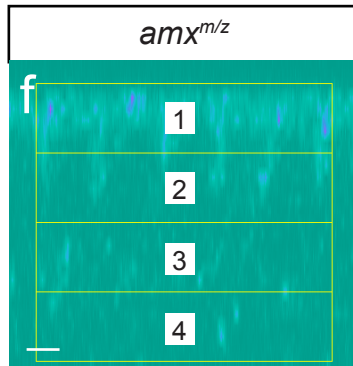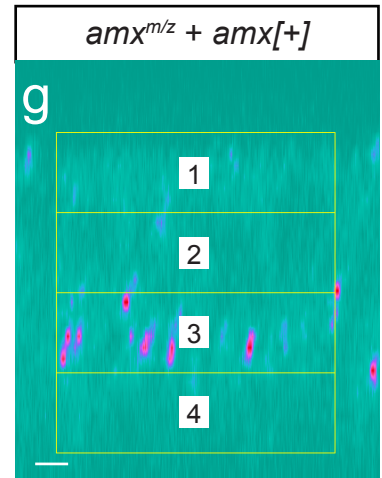

Stain: anti-DI  
Bar = 5  $\mu$ m

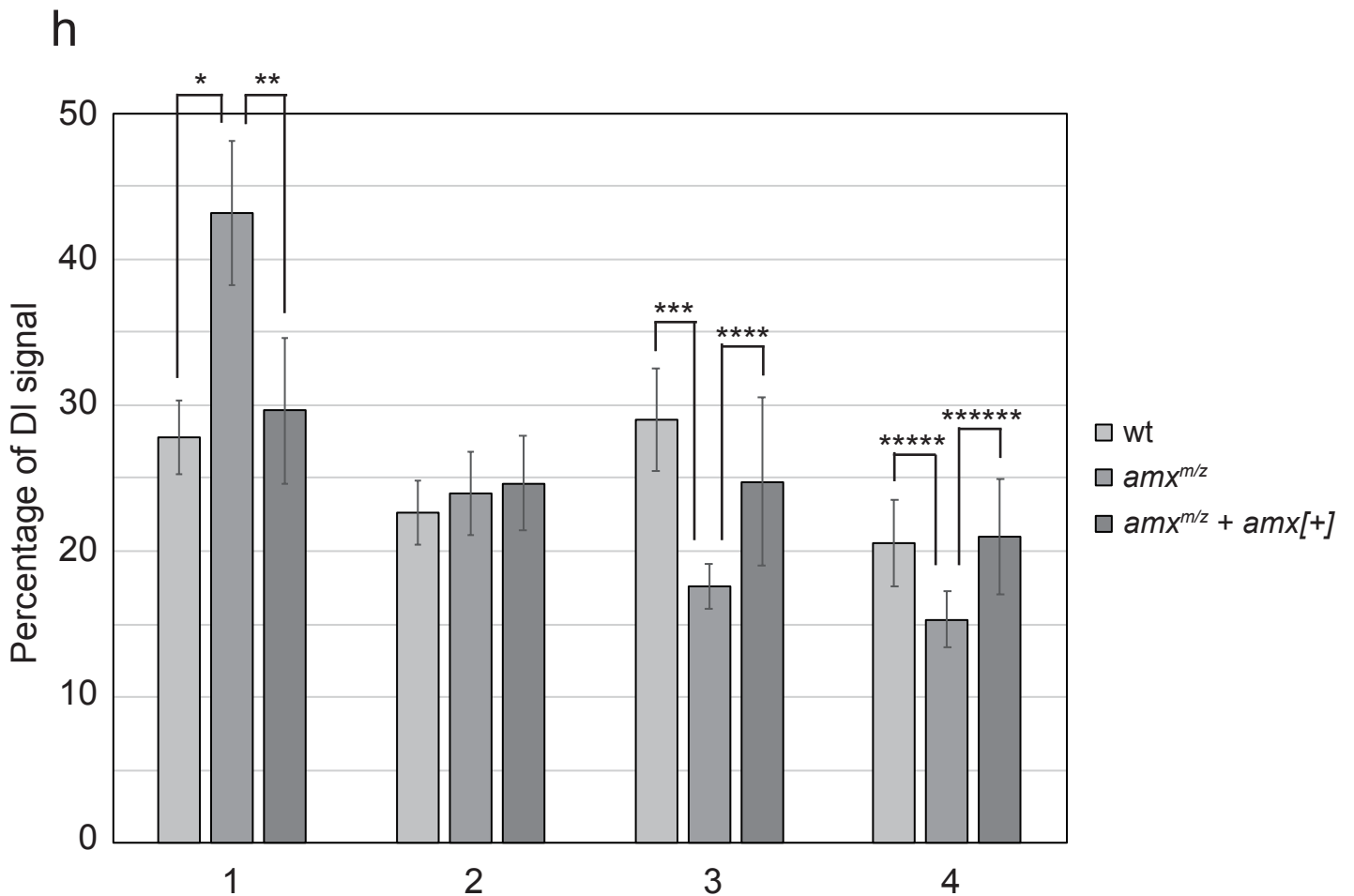

Supplement: Supplementary file 26 — Supplementary Information 26. [file 41598_2023_46866_MOESM26_ESM.pdf]

Supplementary Figure S13 (continued 2)

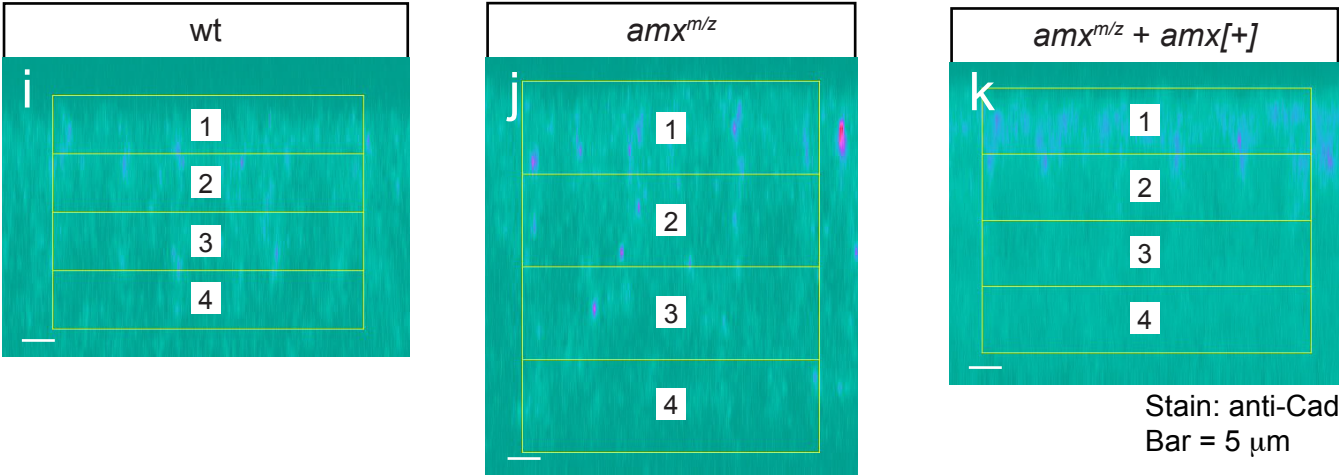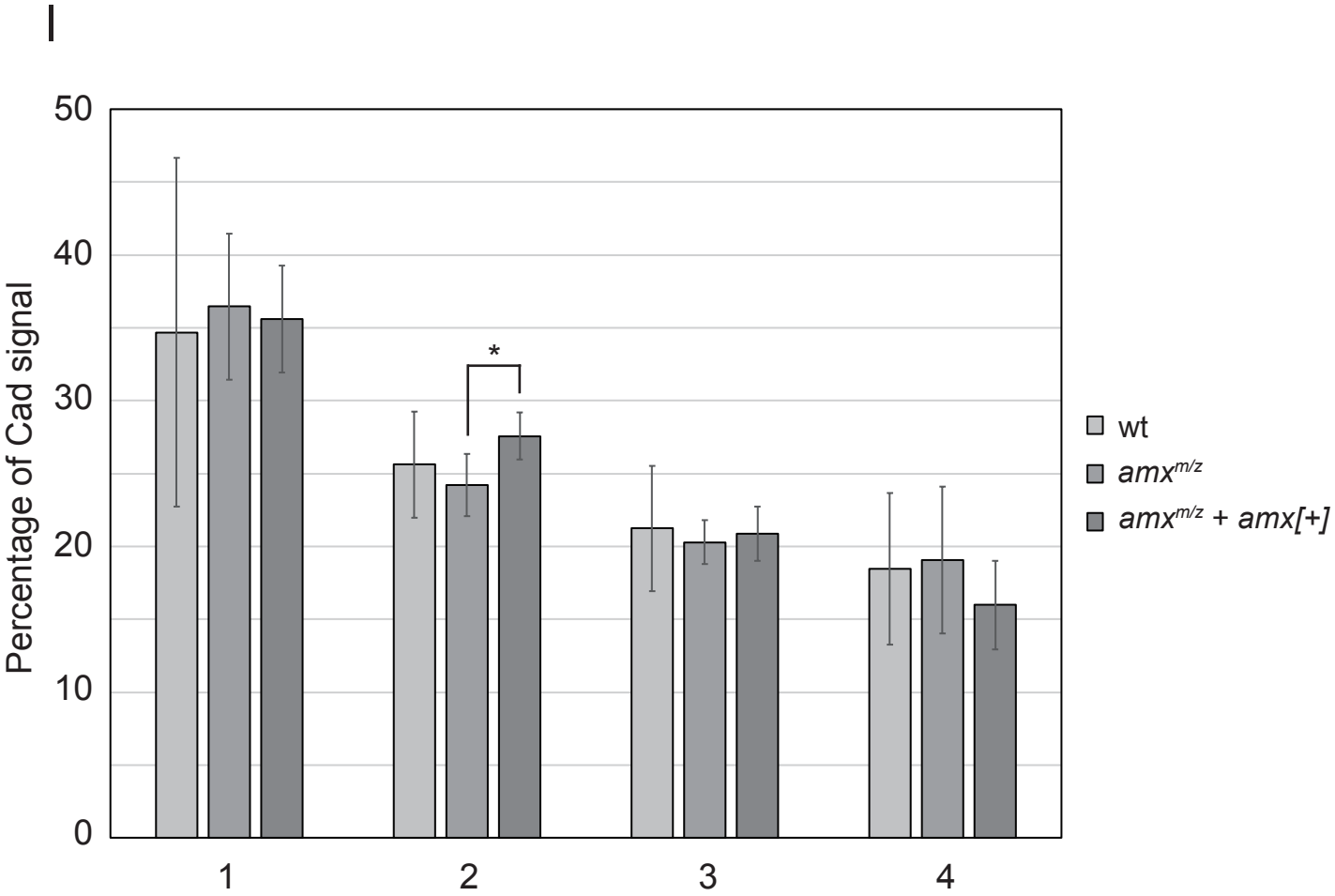

Supplement: Supplementary file 27 — Supplementary Information 27. [file 41598_2023_46866_MOESM27_ESM.pdf]
