# Supplementary material for: TM2D3, a mammalian homologue of Drosophila neurogenic gene product Almondex, regulates surface presentation of Notch receptors
Source: Sci Rep. 2023 Nov 27;13:20913. doi: 10.1038/s41598-023-46866-7 (PMC10684865; doi:10.1038/s41598-023-46866-7)
Supplement: Supplementary file 23 — Supplementary Information 23. [file 41598_2023_46866_MOESM23_ESM.pdf]

Supplementary Figure S11 (continued)

Figure 5a

Whole cell lysates

Experiment #2

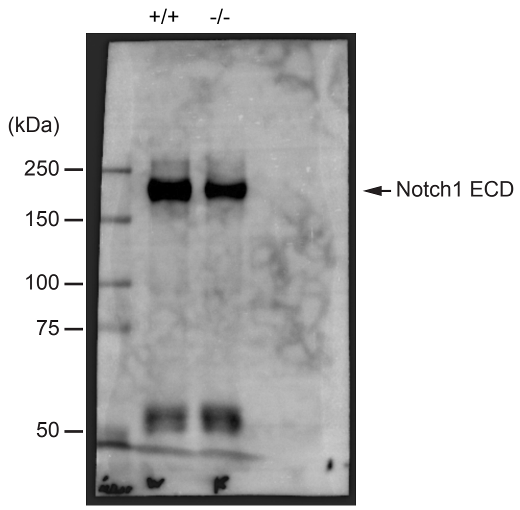

Blot: anti-Notch1 ECD

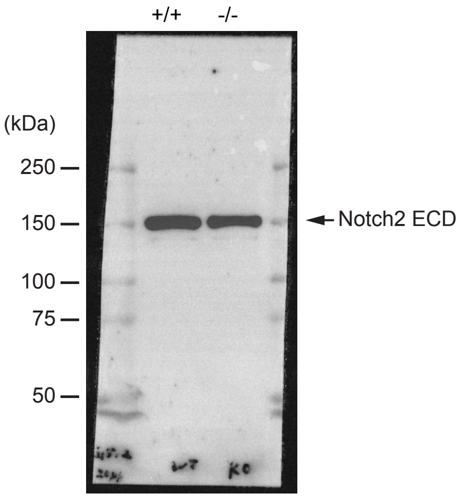

Blot: anti-Notch2 ECD

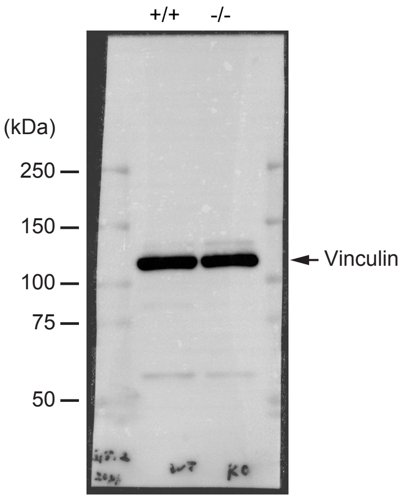

Blot: anti-Vinculin

Experiment #3

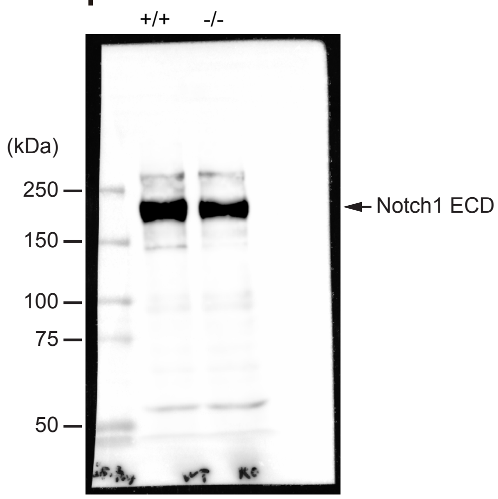

Blot: anti-Notch1 ECD

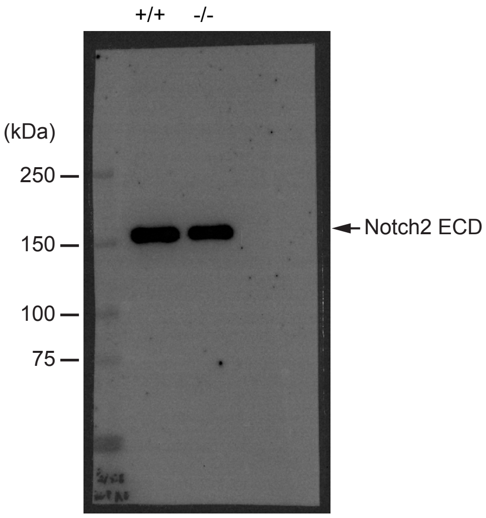

Blot: anti-Notch2 ECD

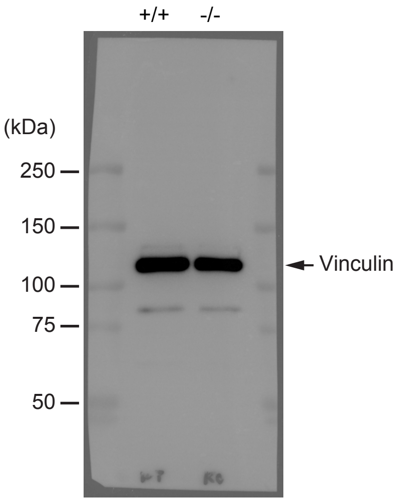

Blot: anti-Vinculin

Figure 5d

Surface biotinylated, avidin-affinity precipitated fractions

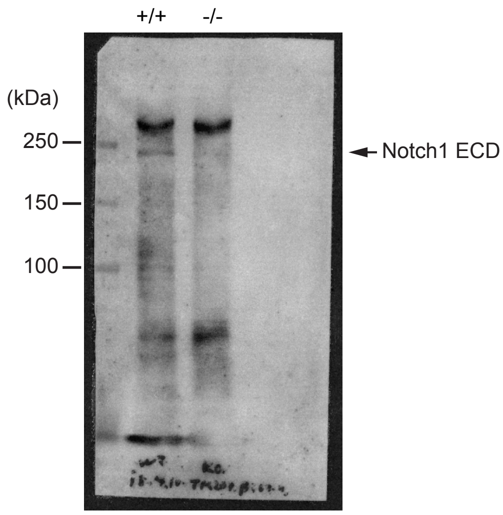

Blot: anti-Notch1 ECD

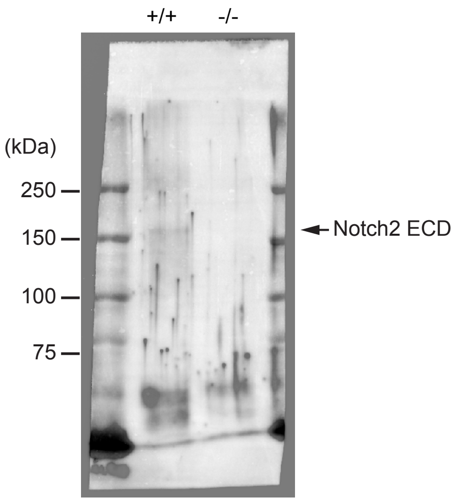

Blot: anti-Notch2 ECD

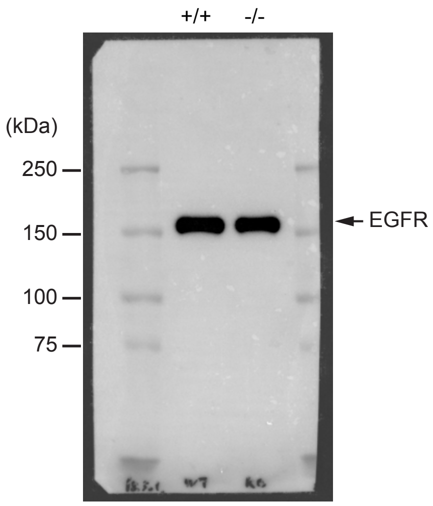

Blot: anti-EGFR
