# Supplementary material for: TM2D3, a mammalian homologue of Drosophila neurogenic gene product Almondex, regulates surface presentation of Notch receptors
Source: Sci Rep. 2023 Nov 27;13:20913. doi: 10.1038/s41598-023-46866-7 (PMC10684865; doi:10.1038/s41598-023-46866-7)
Supplement: Supplementary file 28 — Supplementary Information 28. [file 41598_2023_46866_MOESM28_ESM.docx]

**Supplementary Information**

**TM2D3, a mammalian homologue of *Drosophila* neurogenic gene product Almondex, regulates surface presentation of Notch receptors**

Wataru Masuda^1,2^, Tomoko Yamakawa^3^, Rieko Ajima^4^, Katsuya Miyake^5^, Toshifumi Umemiya^1,6^, Kazuhiko Azuma^1^, Jun-ichi Tamaru^2^, Makoto Kiso^4^, Puspa Das^3^, Yumiko Saga^4^, Kenji Matsuno^3^ & Motoo Kitagawa^1,7, 8^*

**Supplementary Figure S1 | Primary structure of TM2D3.**

(**a**) A comparison of primary sequences of TM2D3v1 and Almondex. Sequence alignment was performed using the DNA Strider program^1^. Signal peptides were predicted using the SignalP 4.1 algorithm^2^. Transmembrane domains (TM1 and TM2) were assigned as described^3^. The overall homology between the two sequences is 34.4%. Note that even in the less well-conserved extracellular domain, cysteine residues (marked in yellow) that may be important for the higher-order structure are well conserved. Asn^184^, which is the last natural residue of the protein encoded by *amx*^1^ allele, is marked in cyan.

(**b**) Primary sequences of TM2D3v1 and v2. *N*-glycosylation sites predicted by NetNGlyc 1.0 Server (http://www.cbs.dtu.dk/services/NetNGlyc/) are in green boxes. *O*-glycosylation sites predicted by NetOGlyc 4.0 Server (http://www.cbs.dtu.dk/services/NetOGlyc/) are marked in green.

**Supplementary Figure S2 | Original Western blots from Figure 1.**

The original, uncropped Western blots are shown. Molecular weight markers are included. Figure 1a, b, and c are included. For Fig. 1b and c, quantitation is included.

**Supplementary Figure S3 | Original Western blots from Figure 2a and their replicates.**

The original, uncropped Western blots (Experiment #1) are shown. Also shown are the blots from the replicate experiments to make up Fig. 2b (Experiments #2 and #3). Molecular weight markers are included.

**Supplementary Figure S4 | S1 site dependency of NOTCH1 activation by transient co-transfection of TM2D3.**

(**a**) Failed activation of a loopout (LO) NOTCH1 construct by co-expression of TM2D3 in U2OS cells. Cells were transiently transfected with vectors for the indicated proteins. Activation of the LO NOTCH1 receptor was assessed by a luciferase assay as in Fig. 2c.

(**b**) Dependence on S1 cleavage of Notch1 activation by TM2D3. Experiments were conducted as in Fig. 3a. 293T cells were transiently transfected with vectors for the indicated proteins or empty vector (Vector) as a control. Immunoblotting was performed with the indicated antibodies.

(**c**) Lack of apparent effect of AT-EK1 on the expression of TM2D3. 293T cells were transiently transfected with vectors for the indicated proteins. Immunoblotting was performed with the indicated antibodies.

**Supplementary Figure S5 | Schematic diagrams of Notch1 constructs.**

(**a**) A schematic diagram of Notch1 constructs used in Fig. 3, 4, and Supplementary Fig. S4b, S6. The EGF repeats 11 and 12 deleted in Notch1 FL-ΔEGF11/12-HA are colored in gray. A summary of experiments presented in Fig. 3a, 3b, 4b, and 4c is also shown.

(**b**) A schematic diagram of NOTCH1 constructs used in Fig. 4d.

**Supplementary Figure S6 | Effect of ligand stimulation on the activation of the Notch1 by transient co-transfection of TM2D3 and sensitivity of the activation against inhibitors of the canonical Notch signaling pathway.**

(**a**) Activation of the Notch1 construct with full-length ectodomain by co-expression of TM2D3 in 293T cells and effect of ligand stimulation on the activation. Cells were transfected with vectors for the indicated proteins or empty vector (-) as a control. They were then left untreated (-) or cocultured with NALM-6-based cells stably overexpressing JAGGED1 (JAGGED1) or those transfected with empty vector control (Vector). Immunoblotting was performed with the indicated antibodies.

(**b**) Sensitivity of the activation of the Notch1 construct against inhibitors of the canonical Notch signaling pathway. Cells were transfected with vectors for the indicated proteins or empty vector (Vector) as a control and were cocultured with the cell line stably expressing JAGGED1. Cells were also incubated with the indicated inhibitors or their vehicle as a control. Immunoblotting was performed with the indicated antibodies.

**Supplementary Figure S7 | Original Western blots from Figure 3.**

The original, uncropped Western blots are shown. Molecular weight markers are included. Figure 3a, b, c, d, e, and f are included.

**Supplementary Figure S8 | Original Western blots from Figure 4.**

The original, uncropped Western blots are shown. Molecular weight markers are included. Figure 4a, b, c, d, e, and f are included.

**Supplementary Figure S9 | Deletion mutants of TM2D3v2.**

(**a**) A schematic diagram of TM2D3v2 and its derivatives. F: FLAG tag.

(**b**) Expression of deletion mutants of TM2D3v2 FLAG. 293T cells were transiently transfected with vectors for the indicated proteins. Immunoblotting was conducted with the indicated antibody. To show the expression of the Δ111-152 mutant, an enhanced image is also presented.

(**c**) Original Western blots from Supplementary Fig. S9b. The original, uncropped Western blots are shown. Molecular weight markers are included.

**Supplementary Figure S10 | Targeted disruption of murine *Tm2d3* gene.**

(**a**) Schematics of the wild-type *Tm2d3* allele around exon 5 and the mutant allele. Positions of PAM sequence, 20-nucleotide guides (gRNA), and sequences of PCR primers are indicated.

(**b**) PCR analysis of DNA from wild-type (+/+), heterozygous (+/-), and homozygous (-/-) mice. PCR with wild-type or mutant alleles would yield a product of 681 base pairs (bp) or 381 bp, respectively.

**Supplementary Figure S11 | Original Western blots from Figure 5 and the replicates.**

The original, uncropped Western blots from Fig. 5a (Experiment #1) and 5e are shown. Also shown are the blots from the replicate experiments to make up Fig. 5b (Experiments #2 and #3). All the membrane filters were cut prior to blotting with antibodies.

**Supplementary Figure S12 | Pseudocolor (fire) images of ventral mesodermal cells used to count numbers of the NECD- and Dl-containing dots.**

(**a**-**c**) Pseudocolor (fire) images of ventral mesodermal cells used to count numbers of the NECD-containing dots shown in Fig. 6g. Upper: apical, bottom: basal. Width of all the pictures was standardized at 80 μm. Dots that were counted are indicated by circles.

(**d**-**f**) Pseudocolor (fire) images of ventral mesodermal cells used to count numbers of the Dl-containing dots shown in Fig. 6h. Analysis was conducted as in **a**-**c**.

**Supplementary Figure S13 | Decrease in the basal region and accumulation in the apical region of NECD and Dl in the ventral presumptive endodermal cells of *amx*-deficient early *Drosophila* embryos.**

(**a**-**c**) Pseudocolor (ice) images of ventral mesodermal cells used to quantitate the fluorescent signals of the NECD epitopes. Upper: apical, bottom: basal. Rectangle areas that served as the regions of interest (ROI) are indicated.

(**d**) Mean gray values of four ROIs (1-4) as exemplified in a-c were measured. For each category, three embryos were analyzed, and for each embryo, three pictures were analyzed. The error bars indicate the standard deviations (n = 9). 1. *F*_(2,24)_ = 13.4, *P* = 1.2 × 10^-4^; 2. *F*_(2,24)_ = 6.1, *P* = 7.3 × 10^-3^; 3. *F*_(2,24)_ = 18.0, *P* = 1.7 × 10^-5^; 4. *F*_(2,24)_ = 12.9, *P* = 1.6 × 10^-4^ using a one-way ANOVA. **P* = 5.7 × 10^-4^; ***P* = 3.8 × 10^-3^; ****P* = 1.4 × 10^-5^; *****P* = 2.0 × 10^-4^ by Dunnett’s multiple comparison test.

(**e**-**g**) Pseudocolor (ice) images of ventral mesodermal cells used to quantitate the fluorescent signals of the Dl epitopes. Upper: apical, bottom: basal. Rectangle areas that served as the regions of interest (ROI) are indicated.

(**h**) Mean gray values of four ROIs (1-4) as exemplified in e-g were measured. For each category, three embryos were analyzed, and for each embryo, three pictures were analyzed. The error bars indicate the standard deviations (n = 9). 1. *F*_(2,24)_ = 34.4, *P* = 8.8 × 10^-8^; 2. *F*_(2,24)_ = 1.2, *P* = 3.1 × 10^-1^; 3. *F*_(2,24)_ = 19.0, *P* = 1.1 × 10^-5^; 4. *F*_(2,24)_ = 9.5, *P* = 9.0 × 10^-4^ using a one-way ANOVA. **P* = 1.6 × 10^-7^; ***P* = 1.3 × 10^-6^; ****P* = 5.3 × 10^-6^; *****P* = 1.9 × 10^-2^; ******P* = 2.5 × 10^-3^; *******P* = 1.4 × 10^-2^ by Dunnett’s multiple comparison test.

(**i**-**k**) Pseudocolor (ice) images of ventral mesodermal cells used to quantitate the fluorescent signals of the Cad epitopes. Upper: apical, bottom: basal. Rectangle areas that served as the regions of interest (ROI) are indicated.

(**l**) Mean gray values of four ROIs (1-4) as exemplified in i-k were measured. For each category, three embryos were analyzed, and for each embryo, three pictures were analyzed. The error bars indicate the standard deviations (n = 9). 1. *F*_(2,24)_ = 1.1 × 10^-1^, *P* = 8.9 × 10^-1^; 2. *F*_(2,24)_ = 3.8, *P* = 3.8 × 10^-2^; 3. *F*_(2,24)_ = 2.6 × 10^-1^, *P* = 7.8 × 10^-1^; 4. *F*_(2,24)_ = 1.2, *P* = 3.2 × 10^-1^ using a one-way ANOVA. **P* = 2.1 × 10^-2^ by Dunnett’s multiple comparison test.

**Supplementary Table S1 | Numbers of mice/eggs used for the targeted disruption of murine *Tm2d3* gene.**

**Supplementary Methods**

**Quantitation of the signals of Western blots.** To quantitate the signals obtained with Odyssey CLx (LI-COR), the images were accessed with Image Studio software (LI-COR) accompanied by the system. Signals of the near infrared dye in the rectangles were normalized with those of REVERT Total Protein Stain (LI-COR) in the corresponding lane.

**Plasmids and an antibody.** The loopout variant (NOTCH1-GAL4-LO) of which the S1 cleavage loop had been deleted from NOTCH1-GAL4 was as described^4^. Human JAGGED1 cDNA^5^ was cloned in the pMX retrovirus vector^6^. An antibody against mammalian E-cadherin (NCH-38; M3612) was obtained from Dako.

**Cell culture and transfection.** Human pre-B acute lymphoblastic leukemia NALM-6 cells^7^ and their derivatives were maintained in Iscove's modified Dulbecco's medium containing FBS (10%). Recombinant retroviruses were produced by co-transfecting pMX vector control or pMX JAGGED1 with amphotropic helper virus DNA^8^ into 293T cells. NALM-6 cells were infected with either of the recombinant retrovirus by culturing in the conditioned medium in the presence of polybrene. For ligand stimulation of Notch1, 293T cells were cocultured with X-irradiated (6 Gy) NALM-6-pMX or NALM-6-pMX JAGGED1 cells 20 hours after transfection. Twenty-four hours after the launch of the coculture, the cells were washed by phosphate buffered saline solution (PBS) and cell extracts were prepared^9^. As NALM-6 cells are floating cells in nature, almost all NALM-6-derived cells were washed out with PBS. Where indicated, a γ-secretase inhibitor, DAPT (N-[(3,5-Difluorophenyl)acetyl]-L-alanyl-2-phenyl)glycine-1,1-dimethylethyl ester: γ-Secretase Inhibitor IX; Calbiochem**)** (10 μM) and/or an ADAM inhibitor Batimastat (BB94; Tocris) (10 μM) were added to the medium 20 hours after the transfection.

**Targeting of *Tm2d3* gene in the mouse genome** **and preparation of primary cultured embryonic fibroblasts.** Cas9-target sites were selected using Optimized CRISPR Design (http://crispr.mit.edu) to delete the exon 5 of *Tm2d3* gene (Supplementary Fig. S10a). Oligonucleotides with the target sequences (5’- TTAACTTAGGGCTAACCACA-3’ and 5’- TACTCAGACGGAGACGAGCC-3’) were synthesized and cloned individually in pX330-U6-Chimeric_BB-CBh-hSpCas9 (Addgene). To induce hyperovulation, female B6C3F1 mice (F1 hybrid of C57BL/6 and C3H strains) (CLEA Japan) were injected with pregnant mare's serum gonadotropin (5 units/mouse) (ASKA Animal Health). Forty-eight hours later, the mice were injected with human chorionic gonadotropin (5 units/mouse) (ASKA Animal Health) and mated with male B6C3F1 mice (CLEA Japan). On the following day, fertilized eggs were obtained from the female mice holding plugs and injected with a mixture of the two clones of pX330-U6-Chimeric_BB-CBh-hSpCas9 containing each of the target sequence. On the following day, the two-cell stage embryos were transplanted into the uteri of pseudopregnant foster mothers (MCH; CLEA Japan). After birth, genomic DNAs obtained from tails of the mice were assessed for the targeted allele via a PCR using Tm2d3-F (5’-CCCGTTATCATGGGATTTTATTGT-3’) and Tm2d3-R (5’-ATTTTAGTCATTCAGGTGGGCTTG-3’) as primers. Numbers of the mice used for these experiments are summarized in Supplementary Table S1. As a result of two courses of the experiments, one mouse was identified to produce a shorter PCR fragment. Deletion of the sequence including the exon 5 of *Tm2d3* gene was confirmed by sequencing of the amplified DNA (Supplementary Fig. S10b). The allele could be transmitted to the following generations (Supplementary Fig. S10b). The mice heterozygous for the targeted allele were apparently healthy and fertile and maintained by crossing to B6C3F1 mice. The phenotypes of the mice with the targeted allele will be described elsewhere.

Primary cultured embryonic fibroblasts were isolated from 13.5- or 14.5-day post-coitum embryos generated by crossings between the heterozygous mice. The cells were maintained in DMEM containing FBS (10%). Genotypes of the cells were identified by the PCR. Using the five pregnant heterozygous mice, seven pairs of wild type and homozygously targeted cells derived from a litter were obtained.

All mice were kept under specific pathogen-free conditions (free from *Citrobacter rodentium*, *Corynebacterium kutscheri*, *Mycoplasma pulmonis*, *Pasteurella pneumotropica*, *Psudomonas aeruginosa*, *Salmonella* species, *Clostridium piliforme*, ectromelia virus, lymphocytic choriomeningitis virus, mouse hepatitis virus, Sendai virus, ectoparasites, intestinal protozoa, pinworm, *Helicobacter bilis*, *Helicobacter hepaticus*, and *Pneumocystis murina* in the National Institute of Genetics, Japan; free from *Corynebacterium kutscheri*, *Mycoplasma pulmonis*, *Salmonella* genus, *Clostridium piliforme*, *Citrobacter rodentium*, *Rodentibacter pneumotropicus*, *Rodentibacter heylii,* Sendai virus, ectromelia virus, lymphocytic choriomeningitis virus, mouse hepatitis virus, *Giardia*, *Spironucleus muris*, *Myobia musculi*, *Myocoptes musculinus*, *Radfordia affinis*, *Radfordia ensifera*, *Syphacia muris*, *Syphacia obvelata*, and *Aspiculuris tetraptera* in International University of Health and Welfare). All these experiments were approved by the Animal Experiment Committee of the National Institute of Genetics, Japan or International University of Health and Welfare Narita Campus. The experiments were conducted by the Division for Development of Genetic-Engineered Mouse Resource of the National Institute of Genetics and the Department of Biochemistry, International University of Health and Welfare School of Medicine in compliance with regulations of the Institute or the University, guidelines of the Ministry of Education, Culture, Sports, Science and Technology of Japan, and the ARRIVE guidelines (https://arriveguidelines.org).

**Quantitation of the fluorescent signals of cells from *Drosophila* embryos.** z-stack images were accessed with ImageJ^10^. They were first converted to pseudocolor images with the ice lookup table. In each image, a rectangular area containing about five epithelial cells was selected. This area was then divided into four equal-sized parts, which served as the regions of interest (ROI), as shown in Supplementary Fig. S14a-c and e-g. Mean gray values of the ROIs were measured and the percentage of each value among the total of the four values was calculated.

**References**

1 Marck, C. 'DNA Strider': a 'C' program for the fast analysis of DNA and protein sequences on the Apple Macintosh family of computers. *Nucleic Acids Res* **16,** 1829-1836 (1988).

2 Petersen, T. N., Brunak, S., von Heijne, G. & Nielsen, H. SignalP 4.0: discriminating signal peptides from transmembrane regions. *Nat Methods* **8,** 785-786 (2011).

3 Kajkowski, E. M. *et al.* beta -Amyloid peptide-induced apoptosis regulated by a novel protein containing a g protein activation module. *J Biol Chem* **276,** 18748-18756 (2001).

4 Gordon, W. R. *et al.* Effects of S1 cleavage on the structure, surface export, and signaling activity of human Notch1 and Notch2. *PLoS One* **4,** e6613 (2009).

5 Gray, G. E. *et al.* Human ligands of the Notch receptor. *Am J Pathol* **154,** 785-794 (1999).

6 Kitamura, T. *et al.* Retrovirus-mediated gene transfer and expression cloning: powerful tools in functional genomics. *Exp Hematol* **31,** 1007-1014 (2003).

7 Hurwitz, R. *et al.* Characterization of a leukemic cell line of the pre-B phenotype. *Int J Cancer* **23,** 174-180 (1979).

8 Quelle, D. E., Zindy, F., Ashmun, R. A. & Sherr, C. J. Alternative reading frames of the INK4a tumor suppressor gene encode two unrelated proteins capable of inducing cell cycle arrest. *Cell* **83,** 993-1000 (1995).

9 Ishikawa, H. O. *et al.* Notch deficiency implicated in the pathogenesis of congenital disorder of glycosylation IIc. *Proc Natl Acad Sci U S A* **102,** 18532-18537 (2005).

10 Schneider, C. A., Rasband, W. S. & Eliceiri, K. W. NIH Image to ImageJ: 25 years of image analysis. *Nat Methods* **9,** 671-675 (2012).
